# Phenylselanyl Group Incorporation for “Glutathione Peroxidase-Like” Activity Modulation

**DOI:** 10.3390/molecules25153354

**Published:** 2020-07-24

**Authors:** Magdalena Obieziurska-Fabisiak, Agata J. Pacuła, Lucia Capoccia, Joanna Drogosz-Stachowicz, Anna Janecka, Claudio Santi, Jacek Ścianowski

**Affiliations:** 1 Department of Organic Chemistry, Faculty of Chemistry, Nicolaus Copernicus University, 7 Gagarin Street, 87-100 Torun, Poland; magdao@umk.pl (M.O.-F.); pacula@umk.pl (A.J.P.); 2 Dipartimento di Scienze Farmaceutiche, Universita di Perugia, Via del Liceo 1, 06134 Perugia, Italy; lucia.capoccia@outlook.it (L.C.); claudio.santi@unipg.it (C.S.); 3 Department of Biomolecular Chemistry, Faculty of Medicine, Medical University of Lodz, Mazowiecka 6/8, 92-215 Lodz, Poland; joanna.drogosz@stud.umed.lodz.pl (J.D.-S.); anna.janecka@umed.lodz.pl (A.J.)

**Keywords:** selenides, antioxidant activity, anticancer activity

## Abstract

The ability of organoselenium molecules to mimic the activity of the antioxidant selenoenzyme glutathione peroxidase (GPx) allows for their use as antioxidant or prooxidant modulators in several diseases associated with the disruption of the cell redox homeostasis. Current drug design in the field is partially based on specific modifications of the known Se-therapeutics aimed at achieving more selective bioactivity towards particular drug targets, accompanied by low toxicity as the therapeutic window for organoselenium compounds tends to be very narrow. Herein, we present a new group of Se-based antioxidants, structurally derived from the well-known group of GPx mimics—benzisoselenazol-3(2*H*)-ones. A series of *N*-substituted unsymmetrical phenylselenides with an *o*-amido function has been obtained by a newly developed procedure: a copper-catalyzed nucleophilic substitution by a Se-reagent formed in situ from diphenyl diselenide and sodium borohydride. All derivatives were tested as antioxidants and anticancer agents towards breast (MCF-7) and leukemia (HL-60) cancer cell lines. The highest H_2_O_2_-scavenging potential was observed for *N*-(3-methylbutyl)-2-(phenylselanyl)benzamide. The best antiproliferative activity was found for (−)-*N*-(1*S*,2*R*,4*R*)-menthyl-2-(phenylselanyl)benzamide (HL-60) and ((−)-*N-(1S,2R,3S,6R*)-(2-caranyl))benzamide (MCF-7). The structure–activity correlations, including the differences in reactivity of the obtained phenyl selenides and corresponding benzisoselenazol-3(2*H*)-ones, were performed.

## 1. Introduction

Drug design is a multi-step process, focused on the obtainment of the most specific ligand–receptor interaction correlated to a suitable structural core that is able to equip the molecule with a potential biological activity. The first discovered lead compound is subsequently variously functionalized in order to increase (and maximize) the desired therapeutic activity over the toxicity. The pharmacophore modeling often includes installation of aromatic or heteroaromatic rings, which are easy to introduce, can be further manipulated and are often responsible of the activity [1,2].

**Scheme 1:**
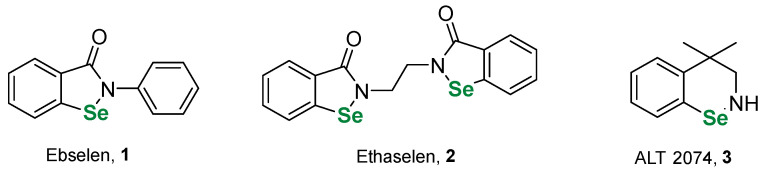
Bioactive organoselenium compounds **1**–**3** that possess aromatic rings in their structure.

In the field of organoselenium chemistry, the design of Se-based therapeutics is often connected with the ability of selenium pharmacophores to mimic the activity of glutathione peroxidase (GPx). Over the years, the role of organoselenium compounds as redox-modulators was well-established with numerous examples of biologically active molecules [3,4,5,6], including the antioxidant agent *N*-phenylbenzisoselenazol-3(2*H*)-one (named as Ebselen) **1**, currently in phase II clinical trial for noise-induced hearing loss [7]. Similarly, to ebselen **1**, a significant number of proven bioactive Se-molecules possess aromatic or heteroaromatic rings as the core of the molecule [3]. Examples are presented in Scheme 1 and also include other Se-therapeutics currently in clinical trial: ethaselen **2**, Trx reductase inhibitor, antitumor agent [8] and 4,4-dimethyl-benziso-2*H*-selenazine **3**, anti-inflammatory therapeutic tested in chronic plaque psoriasis [9].

**Scheme 2:**
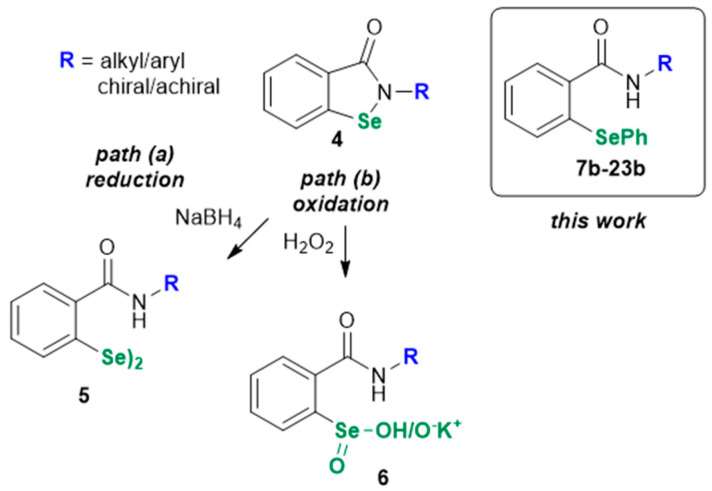
Possible structural modification of *N*-substituted benzisozelenazol-3(2*H*)-ones **4**.

Many research groups continue the study of new strategies and structural modifications to obtain new Se-antioxidants that have high and selective activity. In our previous work, we explored the possibility to improve the GPx-like activity of ebselen with the introduction of specific functionalities that would enable new highly efficient biocatalyst [10,11,12]. Various *N*-aromatic and *N*-aliphatic derivatives **4** were obtained and easily transformed into the corresponding diselenides **5** (path **a**) [13,14,15] and seleninic acids, as well as to their potassium salts **6** (path **b**) [16]. Determination of the antioxidant and antiproliferative potential of all obtained molecules revealed a particular structure–activity relationship. Besides the observed influence of the *N*-substituent on their biological potential, it was also recently highlighted by Santi and co-workers [17], that the form of the Se-moiety is crucial for the specific catalytic activity of the designed GPx-mimics. To further differentiate the structures and to broaden the scope of the tested molecules, we introduced a phenylselanyl group as a new benzisoselenazolone core modification (**7b**–**23b**, Scheme 2).

This modification allowed us to obtain a large group of GPx mimetics **7b**–**23b** and to determine whether the introduction of an additional aromatic ring and the exchange of Se-N for Se-C_ar_ bond is justified in order to obtain higher therapeutic potential of the *N*-substituted ebselen-like antioxidants.

## 2. Results and Discussion

**Scheme 3:**
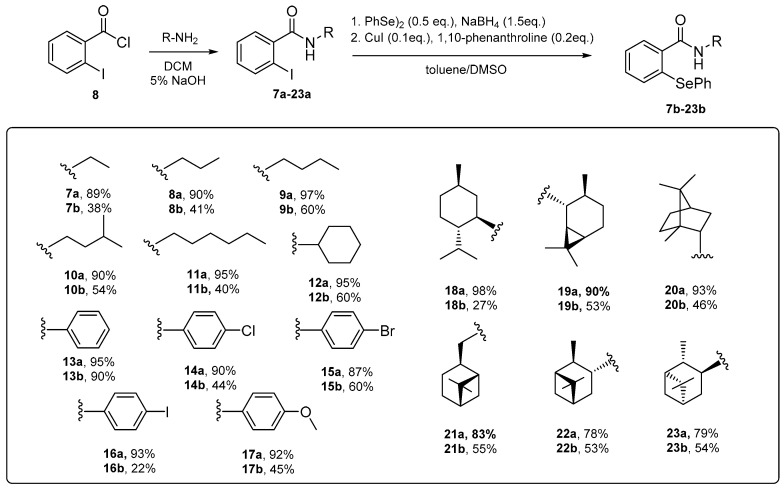
Synthesis of *N*-substituted phenylselenides **7b**–**23b**.

The first step of the research involved the synthesis of *N*-substituted *o*-iodobenzamides **7a**–**23a**. The compounds were obtained through the reaction of the corresponding amines with *o*-iodobenzoic acid chloride **8**. Benzamides **7a**–**23a** were further transformed to the final *N*-aliphatic **7b**–**12b**, *N*-aromatic **13b**–**17b** and chiral *N*-terpenyl [18] phenylselenides **18b**–**23b** by a copper-catalyzed nucleophilic aromatic substitution. The selenium nucleophile was prepared in situ from diphenyl diselenide and sodium borohydride. All derivatives were obtained in moderate to good yields (Scheme 3).

**Scheme 4:**
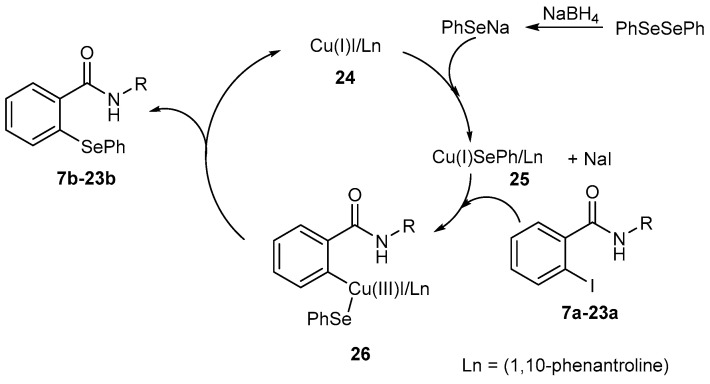
Putative mechanism for the Cu-catalyzed introduction of phenylselenyl group.

Considering the mechanism of the reaction, we assume that the first step includes the base-promoted formation of (1,10-ph)Cu-SePh complex **25**. The oxidative addition to the C_ar_-I bond of the amide leads to the copper(III) complex **26** in which both the arene and Se-nucleophile are ligated to the metal. Next, through the reductive elimination pathway, the final selenide is formed with the regeneration of the (1,10-ph)Cu(I)I catalyst **24** (Scheme 4).

**Scheme 5:**
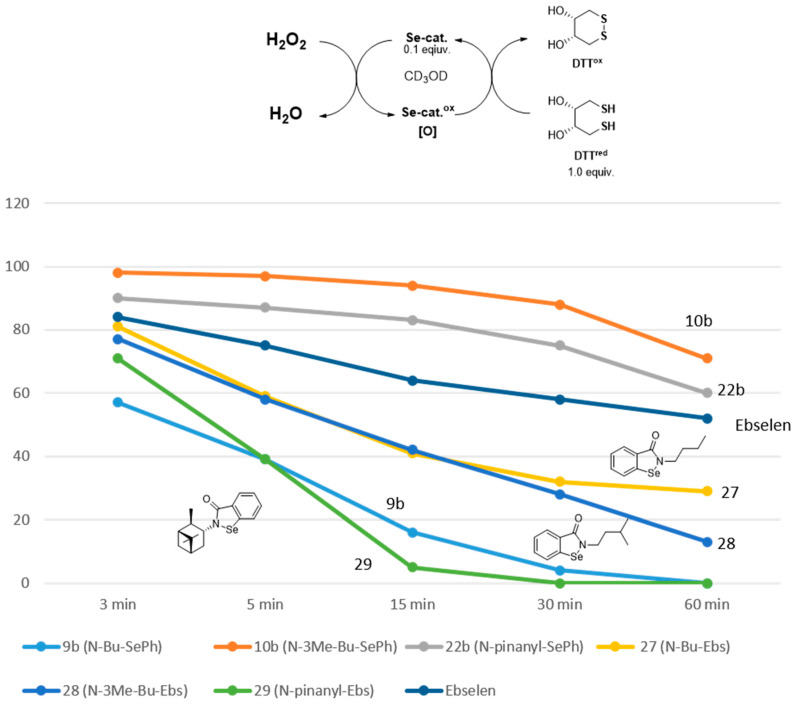
Results of the antioxidant activity measurements.

The final goal of the research was to evaluate the obtained phenylselenides **7b**–**23b** as possible GPx-mimics and anticancer agents. The antioxidant capacity was tested by a conventionally used NMR-activity assay proposed first by Iwaoka and co-workers [19]. The rate of H_2_O_2_ reduction by the Se-catalyst was indirectly evaluated by the oxidation of dithiothreitol as a mimic of the reducing thiol cofactor. The conversion of the dithiol DTT^red^ to the disulphide DTT^ox^ was observed in ^1^HNMR spectra in the specific time intervals. The results for the most active derivatives are presented in Scheme 5. The results obtained for all compounds are reported in Supporting Information.

The highest antioxidant potential was observed for *N*-butyl **9b**, *N*-3-methylbutyl **10b** and *N*-pinanyl phenyl selenide **22b**. The results of the three selected Se-catalysts were compared to corresponding benzisoselenazol-3(2*H*)-ones **27–29**. It could be noticed that the bulkiness of the substituent enhances the H_2_O_2_ scavenging activity of benzisoselenazolones (reactivity: **29** > **28** > **27**) but decreased it for the corresponding phenylselenides (reactivity: **10b** > **22b** > **9b**). For compounds **27**–**29**, the hindrance of the *N*-substituent facilitated the cleavage of the Se-N bond that accelerated the Se-moiety oxidation by hydrogen peroxide. On the contrary, the reaction of -SePh group with H_2_O_2_ proceeded more efficiently when the alkyl chain did not hinder the selenium atom.

**Scheme 6:**
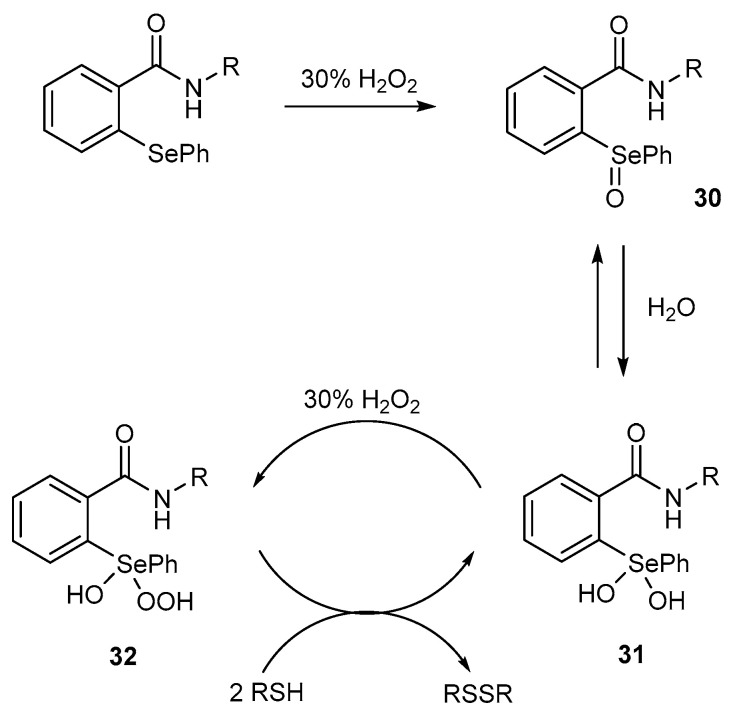
Plausible mechanism for the H_2_O_2_ reduction by the Se-catalysts in the presence of thiols.

To investigate the mechanism of the antioxidant activity, we have performed an additional ^77^Se NMR experiment of the H_2_O_2_-oxidation product of the most reactive *N*-butyl phenylselenide **9b** (the sample was stored for 12 h before the NMR recording). A signal at 853 ppm indicated the formation of corresponding selenooxide. Based on these observations, supported by previous literature reports [20,21], we assume that the possible GPx-like catalytic cycle of the tested phenyl selenides involves the formation of the selenoxide **30**, which is further hydrated to the corresponding hydrated oxide **31**. The final H_2_O_2_ reduction and thiol oxidation proceeds through the reversible formation of the peroxy-hydrated oxide **32** (Scheme 6).

Next, all phenylselenides **7b**–**23b** were evaluated as antiproliferative agents by the MTT (3-(4,5-dimethyldiazol-2-yl)-2,5 diphenyl tetrazolium bromide) assay against breast (MCF-7) and leukemia (HL-60) cell lines. The best results were obtained for *N*-terpene derivatives **18b**, **19b** and compared with the data for the corresponding benzisoselenazol-3(2*H*)-ones **33** and **34** (Table 1). For the rest of the tested samples **7b**–**17b**, **20b**–**23b**, the IC_50_ values were above 50 µM.

Additionally, it was observed that the antiproliferative potential of analogs increased when the phenylselanyl moiety was introduced into the structure, showing that an additional aromatic ring can be beneficial for the compound’s cytotoxicity.

**Scheme 7:**
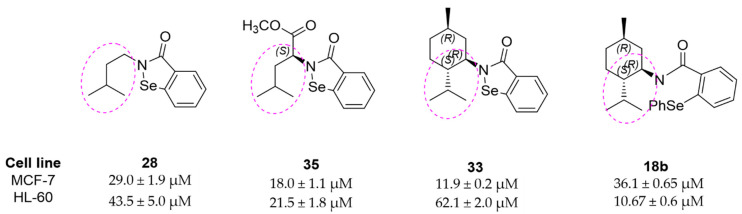
Repetitive carbon chain in the structure of bio-active derivatives **18b**, **28**, **33** and **35**.

We have previously noticed that the internal 2-methylbutyl carbon chain is a repetitive element in the structure of the active benzisoselenazol-3(2*H*)-ones **28**, **33** and **35**, which indicates its potential role as a pharmacophore. Additional carbon chains or functional groups attached to the 2-methylbutyl substituent influenced the inhibitory potential. The antiproliferative activity was the highest for compounds with the carbon chain expanded to the cyclic menthyl functionality, benzisoselenazol-3(2*H*)-one **30** and phenylselenide **18b** with IC_50_ values 11.9 ± 0.2 µM (MCF-7) and 10.7 ± 0.6 µM (HL-60), respectively (Scheme 7).

## 3. Materials and Methods

### 3.1. General

NMR spectra were recorded on Bruker Avance III/400 or Bruker Avance III/700 (Karlsruhe, Germany) for ^1^H and 176.1 MHz or 100.6 MHz for ^13^C (see Supplementary Material). Chemical shifts were recorded relative to SiMe_4_ (δ0.00) or solvent resonance (CDCl_3_ δ7.26, CD_3_OD δ3.31). Multiplicities were given as: s (singlet), d (doublet), dd (double doublet), ddd (double double doublet), t (triplet), dt (double triplet), and m (multiplet). The ^77^Se NMR spectra were recorded on Bruker Avance III/400 or Bruker Avance III/700 with diphenyl diselenide as an external standard. NMR spectra were carried out using ACD/NMR Processor Academic Edition. Melting points were measured with a Büchi Tottoli SPM-20 heating unit (Büchi Labortechnik AG, Flawil, Switzerland) and were uncorrected. Elemental analyses were performed on a Vario MACRO CHN analyzer. Optical rotations were measured in 10-mm cells with a polAAr 3000 polarimeter. Column chromatography was performed using Merck 40-63D 60Å silica gel (Merck, Darmstadt, Germany). Commercially available solvents DMF, DCM, and MeOH (Aldrich, St. Louis, MO, USA) and chemicals were used without further purification.

### 3.2. Procedures and Analysis Data

#### 3.2.1. Synthesis of *N*-substituted *o*-iodobenzamides **7a**–**23a**

2% NaOH (4.4 mL) was added to a solution of an amine (1.0 mmol) in DCM (2 mL). The mixture was cooled to 0 °C and *o*-iodobenzoic acid chloride (1.1 mmol) dissolved in DCM (3 mL) was added dropwise. The reaction mixture was stirred at room temperature for 20 h and the product was extracted with DCM. Combined organic layers were washed with saturated NaHCO_3_ and dried over magnesium sulfate. The solvent was removed under reduced pressure and the product was obtained as white solid.

*((−)-N-(1R,2S,5R)-menthyl)-o-iodobenzamide***18a** Yield: 98%, mp 146–148 °C; [α${]}_{D}^{20}$ = −38.93 (c = 5.73, CHCl_3_); ^1^H NMR (700 MHz, CDCl_3_) *δ* = 0.88 (d, *J* = 7.0 Hz, 3H, CH_3_), 0.92 (s, 3H, CH_3_), 0.93 (s, 3H, CH_3_), 0.98–1.03 (m, 1H), 1.11–1.18 (m, 2H), 1.51–1.57 (m, 2H), 1.69–1.75 (m, 2H), 2.08–2.12 (m, 1H), 2.17–2.20 (m, 1H), 3.95–4.00 (m, 1H), 5.41 (d, *J* = 9.1 Hz, 1H, NH), 7.06–7.09 (m, 1H_ar_), 7.35–7.38 (m, 2H_ar_), 7.84 (dd, *J*_1_ = 0.7, *J*_2_ = 7.7 Hz, 1H_ar_); ^13^C NMR (100.6 MHz, CDCl_3_) *δ* = 16.20 (CH_3_), 21.20 (CH_3_), 22.15 (CH_3_), 23.78 (CH_2_), 26.91 (CH), 31.89 (CH), 34.52 (CH_2_), 42.86 (CH_2_), 48.13 (CH), 50.75 (CH), 92.32 (C_ar_), 128.08 (CH_ar_), 128.12 (CH_ar_), 130.86 (CH_ar_), 139.88 (CH_ar_), 142.93 (C_ar_), 168.61 (C=O); IR: 3230, 2951, 2916, 2867, 1636, 1584, 1540, 1462, 1430, 1385, 1367, 1341, 1325, 1307, 1261, 1161, 1147, 1116, 1107, 1059, 1043, 1014 cm^−1^; Elemental Anal. Calcd for C_17_H_24_INO (385.09): C, 53.00; H, 6.28; N, 3.64 Found: C, 53.18; H, 6.34; N, 3.76.

*((−)-N-(1S,2R,3S,6R)-(2-caranyl))-o-iodobenzamide***19a** Yield: 90%; mp 145–147 °C; [α${]}_{D}^{20}$ = −21.47 (c = 4.77, CHCl_3_); ^1^H NMR (700 MHz, CDCl_3_) 0.64–0.68 (m, 1H), 0.94–1.01 (m, 1H), 1.02 (d, *J* = 7.7 Hz, 3H, CH_3_), 1.05 (s, 3H, CH_3_), 1.11 (s, 3H, CH_3_), 1.23–1.28 (m, 1H), 1.55–1.60 (m, 2H), 1.71–1.74 (m, 1H), 1.77–1.82 (m, 1H), 3.54–3.58 (m, 1H), 5.72 (d, *J* = 8.4 Hz, 1H, NH), 7.09 (dt, *J*_1_ = 2.1, *J*_2_ = 8.4 Hz, 1H_ar_), 7.38 (dt, *J*_1_ = 0.7, *J*_2_ = 7.7 Hz, 1H_ar_), 7.43 (dd, *J*_1_ = 2.1, *J*_2_ = 7.7 Hz, 1H_ar_), 7.87 (dd, *J*_1_ = 0.7, *J*_2_ = 7.7 Hz, 1H_ar_); ^13^C NMR (100.6 MHz, CDCl_3_) *δ* = 15.55 (2 × CH_3_), 17.59 (C), 19.03 (CH_2_), 20.28 (CH), 28.71 (CH), 29.25 (CH_3_), 30.83 (CH_2_), 34.92 (CH), 50.41 (CH), 92.38 (C_ar_), 128.14 (CH_ar_), 128.35 (CH_ar_), 130.90 (CH_ar_), 139.96 (CH_ar_), 142.81 (C), 168.50 (C=O); IR: 3249, 2915, 2862, 1656, 1630, 1585, 1546, 1459, 1430, 1375, 1330, 1257, 1115, 1015 cm^−1^; Elemental Anal. Calcd for C_17_H_22_INO (383.27): C, 53.27; H, 5.79; N, 3.65 Found: C, 53.05; H, 5.71; N, 3.53.

*N-bornyl-o-iodobenzamide***20a** Yield: 93%; mp 122–123 °C (lit. [14] mp 119–121 °C); [α${]}_{D}^{20}$ = +11.01 (c = 5.54, CHCl_3_); ^1^H NMR (700 MHz, CDCl_3_) 0.90 (s, 3H, CH_3_), 0.91–0.99 (m, 1H), 0.96 (s, 3H, CH_3_), 0.99 (s, 3H, CH_3_), 1.17–1.21 (m, 1H), 1.41–1.46 (m, 1H), 1.56–1.61 (m, 1H), 1.70 (t, *J* = 9.1 Hz, 1H), 1.76–1.81 (m, 1H), 2.42–2.47 (m, 1H), 4.41–4.45 (m, 1H), 5.78 (d, *J* = 8.4 Hz, 1H, NH), 7.07–7.10 (m, 1H_ar_), 7.35–7.40 (m, 2H_ar_), 7.85 (dd, *J*_1_ = 0.7, *J*_2_ = 7.7 Hz, 1H_ar_); ^13^C NMR (100.6 MHz, CDCl_3_) *δ* = 13.96 (CH_3_), 18.72 (CH_3_), 19.81 (CH_3_), 28.20 (CH_2_), 28.37 (CH_2_), 37.44 (CH_2_), 44.92 (CH), 48.38 (C), 49.70 (C), 54.64 (CH), 92.40 (C_ar_), 128.20 (CH_ar_), 128.42 (CH_ar_), 130.96 (CH_ar_), 139.83 (CH_ar_), 142.84 (C_ar_), 169.45 (C=O); IR: 3319, 2981, 2950, 2877, 1642, 1584, 1561, 1510, 1479, 1459, 1429, 1388, 1374, 1361, 1310, 1290, 1262, 1228, 1205, 1172, 1154, 1115, 1063, 1046, 1012 cm^−1^; Elemental Anal. Calcd for C_17_H_22_INO (383.27): C, 53.27; H, 5.79; N, 3.65 Found: C, 53.11; H, 5.65; N, 3.47.

*(−)-N-(1S,2R,5S)-myrtanyl-o-iodobenzamide***21a** Yield: 83%; mp 142–144 °C; [α${]}_{D}^{20}$ = −8.23 (c = 5.33, CHCl_3_); ^1^H NMR (400 MHz, CDCl_3_) 0.96 (d, *J* = 9.6 Hz, 1H), 1.11 (s, 3H, CH_3_), 1.24 (s, 3H, CH_3_), 1.55–1.64 (m, 1H), 1.88–2.10 (m, 5H), 2.35–2.44 (m, 2H), 3.47–3.51 (m, 2H), 5.79 (bs, 1H, NH), 7.09–7.13 (m, 1H_ar_), 7.37–7.42 (m, 2H_ar_), 7.87 (dd, *J*_1_ = 0.8, *J*_2_ = 8.4 Hz, 1H_ar_); ^13^C NMR (100.6 MHz, CDCl_3_) *δ* = 17.56 (CH_2_), 21.56 (CH_3_), 26.00 (CH_2_), 27.99 (CH_3_), 33.13 (CH_2_), 38.74 (C), 41.22 (CH), 41.36 (CH), 43.92 (CH), 45.75 (CH_2_), 92.41 (C_ar_), 128.16 (CH_ar_), 128.31 (CH_ar_), 130.98 (CH_ar_), 139.84 (CH_ar_), 142.67 (C_ar_), 169.33 (C=O); IR: 3239, 2935, 2903, 2889, 2859, 1636, 1584, 1542, 1462, 1430, 1382, 1364, 1316, 1292, 1260, 1218, 1156, 1113, 1054, 1014 cm^−1^; Elemental Anal. Calcd for C_17_H_22_INO (383.27): C, 53.27; H, 5.79; N, 3.65 Found: C, 53.55; H, 5.84; N, 3.74.

*(−)-N-(1R,2R,3R,5S)-isopinocamphyl-o-iodobenzamide***22a** Yield: 78%; mp 130–132 °C; [α${]}_{D}^{20}$ = −18.67 (c = 4.88, CHCl_3_); ^1^H NMR (400 MHz, CDCl_3_) 0.91 (d, *J* = 10.0 Hz, 1H), 1.12 (s, 3H, CH_3_), 1.26 (s, 3H, CH_3_), 1.28 (s, 3H, CH_3_), 1.73–1.78 (m, 1H), 1.88–1.91 (m, 1H), 1.93–1.97 (m, 1H), 2.01–2.05 (m, 1H), 2.44–2.50 (m, 1H), 2.73–2.80 (m, 1H), 4.48–4.56 (m, 1H), 5.67 (bs, 1H, NH), 7.09–7.14 (m, 1H_ar_), 7.38–7.45 (m, 2H_ar_), 7.88 (dd, *J*_1_ = 0.8, *J*_2_ = 8.0 Hz, 1H_ar_); ^13^C NMR (100.6 MHz, CDCl_3_) *δ* = 20.98 (CH_3_), 23.39 (CH_3_), 28.00 (CH_3_), 35.31 (CH_2_), 36.91 (CH_2_), 38.49 (C), 41.60 (CH), 46.20 (CH), 47.85 (CH), 48.58 (CH), 92.47 (C_ar_), 128.22 (CH_ar_), 128.37 (CH_ar_), 130.94 (CH_ar_), 139.78 (CH_ar_), 142.68 (C_ar_), 168.78 (C=O); IR: 3242, 2980, 2969, 2900, 2867, 1632, 1584, 1556, 1534, 1458, 1428, 1384, 1372, 1348, 1336, 1319, 1301, 1259, 1227, 1160, 1056, 1016 cm^−1^; Elemental Anal. Calcd for C_17_H_22_INO (383.27): C, 53.27; H, 5.79; N, 3.65 Found: C, 53.49; H, 5.87; N, 3.71.

*(+)-N-(1R,2R,3R,5S)-isopinocamphyl-o-iodobenzamide***23a** Yield: 79%; mp 142–144 °C; [α${]}_{D}^{20}$ = +17.17 (c = 6.00, CHCl_3_) ^1^H NMR (400 MHz, CDCl_3_) 0.90 (d, *J* = 10.0 Hz, 1H), 1.11 (s, 3H, CH_3_), 1.25 (s, 3H, CH_3_), 1.27 (s, 3H, CH_3_), 1.72–1.77 (m, 1H), 1.88–1.90 (m, 1H), 1.94–1.96 (m, 1H), 2.02–2.03 (m, 1H), 2.43–2.49 (m, 1H), 2.72–2.79 (m, 1H), 4.49–4.55 (m, 1H), 5.65 (bs, 1H, NH), 7.09–7.13 (m, 1H, 1H_ar_), 7.40–7.44 (m, 2H, 2H_ar_), 7.87 (d, *J* = 7.2 Hz, 1H, 1H_ar_); ^3^C NMR (100.6 MHz, CDCl_3_) *δ* = 20.99 (CH_3_), 23.40 (CH_3_), 28.00 (CH_3_), 35.33 (CH_2_), 36.94 (CH_2_), 38.50 (C), 41.60 (CH), 46.22 (CH), 47.83 (CH), 48.58 (CH), 92.49 (C_ar_), 128.26 (CH_ar_), 128.41 (CH_ar_), 130.97 (CH_ar_), 139.80 (CH_ar_), 142.68 (C_ar_), 168.80 (C=O); IR: 3305, 2962, 2922, 2891, 2863, 1632, 1582, 1526, 1450, 1428, 1378, 1347, 1334, 1313, 1294, 1274, 1257, 1229, 1220, 1163, 1015 cm^−1^; Elemental Anal. Calcd for C_17_H_22_INO (383.27): C, 53.27; H, 5.79; N, 3.65 Found: C, 53.59; H, 5.70; N, 3.78.

#### 3.2.2. Synthesis of *N*-substituted phenylselenides **10**–**15**

To a solution of a diphenyl diselenide (0.5 mmol) in dry toluene (5 mL), sodium borohydride (1.5 mmol) was added and stirred at room temperature. Next, DMSO was added dropwise until the solution discolored. Then, respectively, CuI (0.1 mmol), 1,10-phenanthroline (0.2 mmol) and an amide (1.0 mmol) were added. The mixture was stirred under reflux for 18 h. The solution was cooled to room temperature and brine (5 mL) was added. The product was extracted with chloroform (2 × 10 mL), and the combined organic layers were washed with water (2 × 10 mL), brine (2 × 10 mL) and dried over magnesium sulphate. The solvent was removed under reduced pressure and the obtained crude product was isolated by column chromatography (silica gel, DCM).

*N-ethyl-2-(phenylselanyl)benzamide***7b** Yield: 38%; mp 89–91 °C (lit. [20] mp 84–86 °C); ^1^H NMR (400 MHz, CDCl_3_) δ = 1.27 (t, *J* = 7.2 Hz, 3H, CH_3_), 3.49–3.56 (m, 2H, N-CH_2_), 6.08 (bs, 1H, NH), 7.11–7.13 (m, 1H_ar_), 7.17–7.22 (m, 2H_ar_), 7.35–7.41 (m, 3H_ar_), 7.51–7.54 (m, 1H_ar_), 7.63–7.65 (m, 2H_ar_); ^13^C NMR (100.6 MHz, CDCl_3_) δ = 14.81 (CH_3_), 35.03 (CH_2_), 125.80 (CH_ar_), 127.36 (CH_ar_), 128.48 (CH_ar_), 129.59 (2 × CH_ar_), 130.01 (C_ar_), 130.90 (CH_ar_), 131.34 (CH_ar_), 134.79 (C_ar_), 134.82 (C_ar_), 135.95 (2 × CH_ar_), 168.19 (C=O); ^77^Se NMR (76 MHz, CDCl_3_) δ = 435.56 ppm; IR: 3267, 3069, 2971, 2927, 2869, 1621, 1585, 1553, 1462, 1448, 1437, 1377, 1359, 1311, 1286, 1261, 1167, 1145, 1121, 1093, 1063, 1033, 1018 cm^−1^; Elemental Anal. Calcd for C_15_H_15_NOSe (305.04): C, 59.22; H, 4.97; N, 4.60 Found: C, 59.15; H, 4.89; N, 4.53.

*N-propyl-2-(phenylselanyl)benzamide***8b** Yield: 41%; mp 74–76 °C (lit. [21] mp 78–79 °C); ^1^H NMR (400 MHz, CDCl_3_) δ = 1.01 (t, *J* = 7.6 Hz, 3H, CH_3_), 1.63–1.69 (m, 2H, CH_2_), 3.19–3.46 (m, 2H, N-CH_2_), 6.22 (bs, 1H, NH), 7.09–7.11 (m, 1H, 1H_ar_), 7.16–7.20 (m, 2H, 2H_ar_), 7.34–7.40 (m, 3H, 3H_ar_), 7.51–7.54 (m, 1H, 1H_ar_), 7.62–7.65 (m, 2H, 2H_ar_); ^13^C NMR (100.6 MHz, CDCl_3_) δ = 11.51 (CH_3_), 22.88 (CH_2_), 41.87 (CH_2_), 125.76 (CH_ar_), 127.39 (CH_ar_), 128.51 (CH_ar_), 129.59 (2 × CH_ar_), 129.99 (C_ar_), 130.89 (CH_ar_), 131.21 (CH_ar_), 134.79 (C_ar_), 134.89 (C_ar_), 136.03 (2 × CH_ar_), 168.35 (C=O); ^77^Se NMR (76 MHz, CDCl_3_) δ = 436.03 ppm; IR: 3277, 3054, 2960, 2922, 2870, 1618, 1585, 1549, 1458, 1436, 1380, 1359, 1313, 1288, 1259, 1145, 1100, 1066, 1033, 1017 cm^−1^; Elemental Anal. Calcd for C_16_H_17_NOSe (319.05): C, 60.38; H, 5.38; N, 4.40 Found: C, 60.55; H, 5.42; N, 4.45.

*N-butyl-2-(phenylselanyl)benzamide***9b** Yield: 60%; mp 121–125 °C; ^1^H NMR (700 MHz, CDCl_3_) δ = 0.96 (t, *J* = 7.0 Hz, 3H, CH_3_), 1.40–1.44 (m, 2H, CH_2_), 1.58–1.61 (m, 2H, CH_2_), 3.45–3.48 (m, 2H, N-CH_2_), 6.09 (bs, 1H, NH), 7.07–7.09 (m, 1H_ar_), 7.16–7.19 (m, 2H_ar_), 7.34–7.38 (m, 3H_ar_), 7.49–7.50 (m, 1H_ar_), 7.61–7.63 (m, 2H_ar_); ^13^C NMR (100.6 MHz, CDCl_3_) δ = 13.39 (CH_3_), 19.79 (CH_2_), 31.24 (CH_2_), 39.49 (CH_2_), 125.38 (CH_ar_), 126.97 (CH_ar_), 128.09 (CH_ar_), 129.18 (2 × CH_ar_), 129.59 (C_ar_), 130.48 (CH_ar_), 130.87 (CH_ar_), 134.40 (C_ar_), 134.45 (C_ar_), 135.57 (2 × CH_ar_), 167.87 (C=O); ^77^Se NMR (76 MHz, CDCl_3_) δ = 435.10 ppm; IR: 3277, 3054, 2960, 2922, 2870, 1618, 1585, 1549, 1458, 1436, 1380, 1359, 1313, 1288, 1259, 1145, 1100, 1066, 1033, 1017 cm^−1^; Elemental Anal. Calcd for C_17_H_19_NOSe (333.06): C, 61.45; H, 5.76; N, 4.22 Found: C, 61.29; H, 5.69; N, 4.16.

*N-hexyl-2-(phenylselanyl)benzamide***11b** Yield: 40%; mp 87–89 °C; ^1^H NMR (400 MHz, CDCl_3_) δ = 0.92(t, *J* = 7.2 Hz, 3H, CH_3_), 1.31–1.42 (m, 6H, 3 × CH_2_), 1.59–1.66 (m, 2H, CH_2_), 3.43–3.48 (m, 2H, N-CH_2_), 6.27 (bs, 1H, NH), 7.08–7.12 (m, 1H_ar_), 7.16–7.19 (m, 2H_ar_), 7.34–7.42 (m, 3H_ar_), 7.52–7.54 (m, 1H_ar_), 7.62–7.65 (m, 2H_ar_); ^13^C NMR (100.6 MHz, CDCl_3_) δ = 14.05 (CH_3_), 22.58 (CH_2_), 26.71 (CH_2_), 29.56 (CH_2_), 31.52 (CH_2_), 40.22 (CH_2_), 125.76 (CH_ar_), 127.46 (CH_ar_), 128.49 (CH_ar_), 129.58 (2 × CH_ar_), 130.05 (C_ar_), 130.85 (CH_ar_), 131.20 (CH_ar_), 134.84 (C_ar_), 134.85 (C_ar_), 136.00 (2 × CH_ar_), 168.33 (C=O); ^77^Se NMR (76 MHz, CDCl_3_) δ = 435.14 ppm; IR: 3317, 2957, 2918, 2927, 2871, 2852, 1617, 1585, 1543, 1462, 1434, 1376, 1331, 1306, 1265, 1200, 1189, 1154, 1032, 1020 cm^−1^; Elemental Anal. Calcd for C_19_H_23_NOSe (361.09): C, 63.33; H, 6.43; N, 3.89 Found: C, 63.19; H, 6.34; N, 3.70.

*N-(3-methylbutyl)-2-(phenylselanyl)benzamide***10b** Yield: 54%; mp 76–78 °C; ^1^H NMR (700 MHz, CDCl_3_) δ = 0.95 (d, *J* = 7.0 Hz, 6H, 2 × CH_3_), 1.49–1.52 (m, 2H, CH_2_), 1.67–1.72 (m, 1H, CH), 3.46–3.49 (m, 2H, N-CH_2_), 6.05 (bs, 1H, NH), 7.08–7.09 (m, 1H, 1H_ar_), 7.16–7.19 (m, 2H_ar_), 7.34–7.38 (m, 3H_ar_), 7.48–7.50 (m, 1H_ar_), 7.61–7.62 (m, 2H_ar_); ^13^C NMR (100.6 MHz, CDCl_3_) δ = 22.49 (CH_3_), 25.99 (CH), 38.43 (CH_2_), 38.49 (CH_2_), 125.83 (CH_ar_), 127.38 (CH_ar_), 128.47 (CH_ar_), 129.59 (2 × CH_ar_), 130.04 (C_ar_), 130.88 (CH_ar_), 131.39 (CH_ar_), 134.68 (C_ar_), 134.96 (C_ar_), 135.90 (2 × CH_ar_), 168.23 (C=O); ^77^Se NMR (76 MHz, CDCl_3_) δ = 435.83 ppm; IR: 3315, 2955, 2927, 2870, 1624, 1585, 1564, 1564, 1536, 1462, 1434, 1384, 1364, 1342, 1304, 1284, 1268, 1255, 1226, 1158, 1023 cm^−1^; Elemental Anal. Calcd for C_18_H_21_NOSe (347.08): C, 62.43; H, 6.11; N, 4.04 Found: C, 62.61; H, 6.19; N, 4.13.

*N-cyclohexyl-2-(phenylselanyl)benzamide***12b** Yield: 60%; mp 183–185 °C (lit. [22] mp 179–181 °C); ^1^H NMR (400 MHz, CDCl_3_) δ = 1.21–1.31 (m, 3H), 1.40–1.50 (m, 2H, CH_2_), 1.65–1.69 (m, 1H), 1.74–1.80 (m, 2H, CH_2_), 2.05–2.09 (m, 2H, CH_2_), 3.97–4.07 (m, 1H, N-CH), 5.32 (bs, 1H, NH), 7.09–7.11 (m, 1H_ar_), 7.17–7.23 (m, 2H_ar_), 7.35–7.41 (m, 3H_ar_), 7.50–7.54 (m, 1H_ar_), 7.60–7.65 (m, 2H_ar_); ^13^C NMR (100.6 MHz, CDCl_3_) δ = 24.83 (2 × CH_2_), 25.55 (CH_2_), 33.07 (2 × CH_2_), 48.87 (CH), 125.76 (CH_ar_), 127.33 (CH_ar_), 128.42 (CH_ar_), 129.55 (2 × CH_ar_), 130.01 (C_ar_), 130.80 (CH_ar_), 131.26 (CH_ar_), 134.60 (C_ar_), 135.04 (C_ar_), 135.88 (2 × CH_ar_), 167.38 (C=O); ^77^Se NMR (76 MHz, CDCl_3_) δ = 434.17 ppm; IR: 3251, 3053, 2924, 2849, 1618, 1583, 1541, 1459, 1448, 1436, 1377, 1337, 1299, 1283, 1256, 1240, 1191, 1149, 1120, 1081, 1066, 1030, 1018 cm^−1^; Elemental Anal. Calcd for C_19_H_21_NOSe (359.08): C, 63.68; H, 5.91; N, 3.91 Found: C, 63.52; H, 5.86; N, 3.83.

*N-phenyl-2-(phenylselanyl)benzamide***13b** Yield: 90%; mp 139–140 °C (lit. [23] mp 139–141 °C); ^1^H NMR (700 MHz, CDCl_3_) 7.14–7.18 (m, 2H_ar_), 7.22–7.26 (m, 2H_ar_), 7.33–7.39 (m, 5H_ar_), 7.59–7.61 (m, 4H_ar_), 7.65–7.67 (m, 1H_ar_), 7.87 (bs, 1H, NH); ^13^C NMR (75.5 MHz, CDCl_3_) *δ* = 120.18 (2 × CH_ar_), 124.67 (CH_ar_), 126.16 (CH_ar_), 127.76 (CH_ar_), 128.57 (CH_ar_), 129.05 (2 × CH_ar_), 129.65 (2 × CH_ar_), 129.76 (C_ar_), 131.33 (CH_ar_), 131.86 (CH_ar_), 134.86 (C_ar_), 135.05 (C_ar_), 135.79 (2 × CH_ar_), 137.71 (C_ar_), 166.33 (C=O); ^77^Se NMR (76 MHz, CDCl_3_) δ = 434.60 ppm; IR: 3328, 3046, 1641, 1596, 1581, 1519, 1494, 1435, 1319, 1290, 1271, 1251, 1177, 1152, 1139, 1104, 1074, 1063, 1045, 1025 cm^−1^; Elemental Anal. Calcd for C_19_H_15_NOSe (353.03): C, 64.78; H, 4.29; N, 3.98 Found: C, 64.92; H, 4.34; N, 4.09.

*N-(p-chlorophenyl)-2-(phenylselanyl)benzamide***14b** Yield: 44%; mp 167–169 °C; ^1^H NMR (700 MHz, CDCl_3_) 7.20–7.21 (m, 1H_ar_), 7.24–7.28 (m, 2H_ar_), 7.32–7.38 (m, 5H_ar_), 7.53–7.55 (m, 2H_ar_), 7.58–7.59 (m, 2H_ar_), 7.66 (dd, *J*_1_ = 1.4, *J*_2_ = 7.0 Hz, 1H_ar_), 7.84 (bs, 1H, NH); ^13^C NMR (75.5 MHz, CDCl_3_) *δ* = 121.32 (2 × CH_ar_), 126.32 (CH_ar_), 127.83 (CH_ar_), 128.61 (CH_ar_), 129.08 (2 × CH_ar_), 129.65 (2 × C_ar_), 129.69 (2 × CH_ar_), 131.52 (CH_ar_), 132.11 (CH_ar_), 134.68 (C_ar_), 135.60 (2 × CH_ar_), 136.24 (2 × C_ar_), 166.21 (C=O); ^77^Se NMR (76 MHz, CDCl_3_) δ = 433.36 ppm; IR: 3349, 3053, 1656, 1592, 1579, 1509, 1491, 1457, 1433, 1394, 1354, 1311, 1289, 1235, 1180, 1140, 1118, 1093, 1074, 1046, 1030, 1012, 1000 cm^−1^; Elemental Anal. Calcd for C_19_H_14_ClNOSe (386.99): C, 59.01; H, 3.65; N, 3.62 Found: C, 59.32; H, 3.55; N, 3.76.

*N-(p-bromophenyl)-2-(phenylselanyl)benzamide***15b** Yield: 60%; mp 176–178 °C; ^1^H NMR (400 MHz, CDCl_3_) 7.22–7.24 (m, 1H_ar_), 7.27–7.31 (m, 2H_ar_), 7.35–7.41 (m, 3H_ar_), 7.48–7.53 (m, 4H_ar_), 7.60–7.62 (m, 2H_ar_), 7.68–7.70 (m, 1H_ar_), 7.86 (bs, 1H, NH); ^13^C NMR (75.5 MHz, CDCl_3_) 121.62 (2 × CH_ar_), 126.37 (CH_ar_), 127.86 (CH_ar_), 128.58 (CH_ar_), 129.68 (2 × CH_ar_), 129.98 (2 × C_ar_), 131.51 (CH_ar_), 132.02 (2 × CH_ar_), 132.22 (CH_ar_), 134.53 (C_ar_), 134.88 (C_ar_), 135.51 (2 × CH_ar_), 136.76 (C_ar_), 166.18 (C=O); ^77^Se NMR (76 MHz, CDCl_3_) δ = 433.40 ppm; IR: 3277, 1644, 1589, 1512, 1487, 1457, 1436, 1428, 1390, 1312, 1286, 1250, 1237, 1179, 1164, 1139, 1071, 1030, 1021, 1006 cm^−1^; Elemental Anal. Calcd for C_19_H_14_BrNOSe (430.94): C, 52.93; H, 3.27; N, 3.25 Found: C, 53.11; H, 3.35; N, 3.40.

*N-(p-iodophenyl)-2-(phenylselanyl)benzamide***16b** Yield: 22%; mp 179–181 °C; ^1^H NMR (700 MHz, CDCl_3_) 7.18–7.20 (m, 1H_ar_), 7.23–7.25 (m, 2H_ar_), 7.33–7.35 (m, 2H_ar_), 7.36–7.38 (m, 3H_ar_), 7.57–7.59 (m, 2H_ar_), 7.62–7.65 (m, 2H_ar_), 7.94 (bs, 1H, NH); ^13^C NMR (75.5 MHz, CDCl_3_) 121.87 (2 × CH_ar_), 126.35 (CH_ar_), 127.85 (CH_ar_), 128.60 (CH_ar_), 129.68 (2 × CH_ar_), 129.98 (2 × C_ar_), 131.53 (CH_ar_), 132.19 (CH_ar_), 134.60 (C_ar_), 134.53 (C_ar_), 135.54 (2 × CH_ar_), 137.47 (C_ar_), 137.98 (2 × CH_ar_), 166.18 (C=O); ^77^Se NMR (76 MHz, CDCl_3_) δ = 433.36 ppm; IR: 3348, 3049, 2922, 2852, 1651, 1585, 1506, 1486, 1456, 1436, 1429, 1387, 1352, 1313, 1288, 1233, 1187, 1166, 1139, 1120, 1063, 1045, 1029, 1019, 1002 cm^−1^; Elemental Anal. Calcd for C_19_H_14_INOSe (478.93): C, 47.72; H, 2.95; N, 2.93 Found: C, 47.59; H, 3.01; N, 3.10.

*N-(p-metoxyphenyl)-2-(phenylselanyl)benzamide***17b** Yield: 45%; mp 131–132 °C; ^1^H NMR (700 MHz, CDCl_3_) 3.83 (s, 3H, CH_3_), 6.71 (ddd, *J*_1_ = 0.7, *J*_2_ = 2.8 Hz, *J*_2_ = 8.4 Hz, 1H_ar_), 7.05 (dd, *J*_1_ = 1.4, *J*_2_ = 8.4 Hz, 1H_ar_), 7.18 (dd, *J*_1_ = 0.7, *J*_2_ = 7.7 Hz, 1H_ar_), 7.23–7.28 (m, 3H_ar_), 7.33–7.39 (m, 4H_ar_), 7.60–7.61 (m, 2H_ar_), 7.65 (dd, *J*_1_ = 1.4, *J*_2_ = 7.7 Hz, 1H_ar_), 7.80 (bs, 1H, NH); ^13^C NMR (75.5 MHz, CDCl_3_) 55.37 (CH_3_), 105.74 (CH_ar_), 110.66 (CH_ar_), 112.22 (CH_ar_), 126.22 (CH_ar_), 127.77 (CH_ar_), 128.54 (CH_ar_), 129.64 (2 × CH_ar_), 129.71 (CH_ar_), 131.34 (CH_ar_), 131.96 (CH_ar_), 134.64 (C_ar_), 135.18 (C_ar_), 136.13 (2 × CH_ar_), 138.91 (2 × C_ar_), 160.22 (C_ar_), 166.26 (C=O); ^77^Se NMR (76 MHz, CDCl_3_) δ = 433.96 ppm; IR: 3300, 2922, 1640, 1597, 1581, 1564, 1521, 1489, 1464, 1453, 1427, 1306, 1287, 1273, 1253, 1201, 1172, 1156, 1140, 1038, 1022 cm^−1^; Elemental Anal. Calcd for C_20_H_17_NO_2_Se (383.04): C, 62.83; H, 4.48; N, 3.66 Found: C, 62.69; H, 4.39; N, 3.50.

*(−)-N-(1R,2S,5R)-menthyl-2-(phenylselanyl)benzamide***18b** Yield: 27%, mp 153–155 °C; [α${]}_{D}^{20}$ = −31.11 (c = 2.61, CHCl_3_); ^1^H NMR (400 MHz, CDCl_3_) *δ* = 0.88 (d, *J* = 6.8 Hz, 3H, CH_3_), 0.93 (d, *J* = 6.4 Hz, 3H, CH_3_), 0.94 (d, *J* = 6.8 Hz, 3H, CH_3_), 1.16–1.20 (m, 2H), 1.51–1.59 (m, 3H), 1.71–1.81 (m, 2H), 2.01–2.09 (m, 1H), 2.12–2.18 (m, 1H), 3.95–4.04 (m, 1H), 5.78 (d, *J* = 8.8 Hz, 1H, NH), 7.10–7.12 (m, 1H_ar_), 7.17–7.23 (m, 2H_ar_), 7.34–7.40 (m, 3H_ar_), 7.50–7.52 (m, 1H_ar_), 7.62–7.64 (m, 2H_ar_); ^13^C NMR (100.6 MHz, CDCl_3_) *δ* = 16.26 (CH_3_), 21.25 (CH_3_), 22.17 (CH_3_), 23.85 (CH_2_), 26.99 (CH), 31.93 (CH), 34.56 (CH_2_), 43.02 (CH_2_), 48.25 (CH), 50.63 (CH), 125.79 (CH_ar_), 127.26 (CH_ar_), 128.41 (CH_ar_), 129.57 (2 × CH_ar_), 130.12 (C_ar_), 130.76 (CH_ar_), 131.33 (CH_ar_), 134.51 (C_ar_), 135.42 (C_ar_), 135.86 (2 × CH_ar_), 167.60 (C=O); ^77^Se NMR (76 MHz, CDCl_3_) δ = 434.56 ppm; IR: 3364, 2960, 2936, 2863, 1637, 1582, 1522, 1458, 1435, 1338, 1305, 1255, 1157, 1031, 1020 cm^−1^; Elemental Anal. Calcd for C_23_H_29_NOSe (415.14): C, 66.66; H, 7.05; N, 3.38 Found: C, 66.78; H, 7.13; N, 3.23.

*(−)-N-(1S,2R,3S,6R)-(2-caranyl)-2-(phenylselanyl)benzamide***19b** Yield: 53%; mp 115–116 °C; [α${]}_{D}^{20}$ = −15.11 (c = 6.15, CHCl_3_); ^1^H NMR (400 MHz, CDCl_3_) 0.59–0.62 (m, 1H), 0.65–0.69 (m, 1H), 1.00 (d, *J* = 6.4 Hz, 3H, CH_3_), 1.05 (s, 3H, CH_3_), 1.13 (s, 3H, CH_3_), 1.24–1.32 (m, 1H), 1.55–1.62 (m, 1H), 1.72–1.88 (m, 3H), 3.59–3.65 (m, 1H), 6.15 (d, *J* = 8.8 Hz, 1H, NH), 7.10–7.13 (m, 1H_ar_), 7.17–7.24 (m, 2H_ar_), 7.35–7.42 (m, 3H_ar_), 7.57–7.59 (m, 1H_ar_), 7.64–7.67 (m, 2H_ar_); ^13^C NMR (100.6 MHz, CDCl_3_) *δ* = 15.55 (CH_3_), 17.58 (C), 18.88 (CH_3_), 19.09 (CH_2_), 20.25 (CH), 28.88 (CH), 29.30 (CH_3_), 30.80 (CH_2_), 35.32 (CH), 50.21 (CH), 125.77 (CH_ar_), 127.38 (CH_ar_), 128.43 (CH_ar_), 129.58 (2 × CH_ar_), 130.10 (C_ar_), 130.79 (CH_ar_), 131.32 (CH_ar_), 134.72 (C_ar_), 135.25 (C_ar_), 135.94 (2 × CH_ar_), 167.41 (C=O); ^77^Se NMR (76 MHz, CDCl_3_) δ = 435.56 ppm; IR: 3233, 2920, 2863, 1623, 1582, 1540, 1477, 1459, 1437, 1375, 1331, 1284, 1258, 1243, 1161, 1114, 1086, 1055, 1021 cm^−1^; Elemental Anal. Calcd for C_23_H_27_NOSe (413.13): C, 66.98; H, 6.60; N, 3.40 Found: C, 67.12; H, 6.69; N, 3.51.

*N-borynyl-2-(phenylselanyl)benzamide***20b** Yield: 46%; mp 118–120 °C; [α${]}_{D}^{20}$ = −32.10 (c = 4.71, CHCl_3_); ^1^H NMR (400 MHz, CDCl_3_) 0.93 (s, 3H, CH_3_), 0.94 (s, 3H, CH_3_), 1.02 (s, 3H, CH_3_), 1.17–1.24 (m, 1H), 1.42–1.51 (m, 1H), 1.55–1.62 (m, 2H), 1.72–1.74 (m, 1H), 1.76–1.86 (m, 1H), 2.44–2.52 (m, 1H), 4.46–4.52 (m, 1H), 5.32 (d, *J* = 8.8 Hz, 1H, NH), 7.12–7.14 (m, 1H_ar_), 7.19–7.26 (m, 2H_ar_), 7.34–7.40 (m, 3H_ar_), 7.56–7.58 (m, 1H_ar_), 7.61–7.63 (m, 2H_ar_); ^13^C NMR (100.6 MHz, CDCl_3_) *δ* = 13.81 (CH_3_), 18.71 (CH_3_), 19.84 (CH_3_), 28.27 (CH_2_), 28.43 (CH_2_), 37.72 (CH_2_), 44.97 (CH), 48.26 (C), 49.72 (C), 54.48 (CH), 126.00 (CH_ar_), 127.51 (CH_ar_), 128.39 (CH_ar_), 129.60 (2 × CH_ar_), 130.09 (C_ar_), 130.87 (CH_ar_), 131.64 (CH_ar_), 134.09 (C_ar_), 135.57 (C_ar_), 135.62 (2 × CH_ar_), 168.36 (C=O); ^77^Se NMR (76 MHz, CDCl_3_) δ = 432.53 ppm; IR: 3359, 2950, 2885, 1629, 1582, 1561, 1516, 1476, 1456, 1435, 1389, 1374, 1363, 1309, 1277, 1256, 1223, 1204, 1173, 1152, 1109, 1065, 1030, 1021 cm^−1^; Elemental Anal. Calcd for C_23_H_27_NOSe (413.13): C, 66.98; H, 6.60; N, 3.40 Found: C, 66.79; H, 6.54; N, 3.32.

*(−)-N-(1S,2R,5S)-myrtanyl-2-(phenylselanyl)benzamide***21b** Yield: 55%; mp 115–117 °C; [α${]}_{D}^{20}$ = −8.00 (c = 2.70, CHCl_3_); ^1^H NMR (400 MHz, CDCl_3_) 0.94 (d, *J* = 9.6 Hz, 1H), 1.11 (s, 3H, CH_3_), 1.23 (s, 3H, CH_3_), 1.53–1.63 (m, 2H), 1.87–2.06 (m, 4H), 2.31–2.43 (m, 2H), 3.47–3.51 (m, 2H), 6.13 (bs, 1H, NH), 7.10–7.13 (m, 1H_ar_), 7.17–7.23 (m, 2H_ar_), 7.35–7.42 (m, 3H_ar_), 7.51–7.53 (m, 1H_ar_), 7.62–7.64 (m, 2H_ar_); ^13^C NMR (100.6 MHz, CDCl_3_) *δ* = 19.94 (CH_2_), 23.25 (CH_3_), 26.02 (CH_2_), 27.99 (CH_3_), 33.24 (CH_2_), 38.75 (C), 41.39 (CH), 41.41 (CH), 43.91 (CH), 45.73 (CH_2_), 125.86 (CH_ar_), 127.41 (CH_ar_), 128.43 (CH_ar_), 129.58 (2 × CH_ar_), 130.06 (C_ar_), 130.86 (CH_ar_), 131.44 (CH_ar_), 134.52 (C_ar_), 135.16 (C_ar_), 135.83 (2 × CH_ar_), 168.27 (C=O); ^77^Se NMR (76 MHz, CDCl_3_) δ = 434.53 ppm; IR: 3333, 2980, 2905, 1631, 1584, 1560, 1530, 1462, 1432, 1383, 1364, 1314, 1284, 1254, 1219, 1154, 1058, 1032, 1019, 1001 cm^−1^; Elemental Anal. Calcd for C_23_H_27_NOSe (413.13): C, 66.98; H, 6.60; N, 3.40 Found: C, 67.12; H, 6.69; N, 3.50.

*(−)-N-(1R,2R,3R,5S)-isopinocamphyl-2-(phenylselanyl)benzamide***22b** Yield: 53%; mp 117–119 °C; [α${]}_{D}^{20}$ = −14.75 (c = 4.73, CHCl_3_); ^1^H NMR (400 MHz, CDCl_3_) 0.88 (d, *J* = 10.0 Hz, 1H), 1.11 (s, 3H, CH_3_), 1.22 (d, *J* = 7.2 Hz, 3H, CH_3_), 1.27 (s, 3H, CH_3_), 1.63–1.69 (m, 1H), 1.87–1.89 (m, 2H), 2.00–2.03 (m, 1H), 2.43–2.49 (m, 1H), 2.72–2.79 (m, 1H), 4.48–4.56 (m, 1H), 6.01 (d, *J* = 7.2 Hz, 1H, NH), 7.12–7.14 (m, 1H_ar_), 7.18–7.25 (m, 2H_ar_), 7.35–7.40 (m, 3H_ar_), 7.54–7.56 (m, 1H_ar_), 7.61–7.64 (m, 2H_ar_); ^13^C NMR (100.6 MHz, CDCl_3_) *δ* = 20.88 (CH_3_), 23.38 (CH_3_), 28.01 (CH_3_), 35.34 (CH_2_), 37.21 (CH_2_), 38.45 (C), 41.62 (CH), 46.49 (CH), 47.85 (CH), 48.53 (CH), 125.97 (CH_ar_), 127.51 (CH_ar_), 128.39 (CH_ar_), 129.61 (2 × CH_ar_), 130.08 (C_ar_), 130.84 (CH_ar_), 131.55 (CH_ar_), 134.10 (C_ar_), 135.44 (C_ar_), 135.63 (2 × CH_ar_), 167.72 (C=O); ^77^Se NMR (76 MHz, CDCl_3_) δ = 431.80 ppm; IR: 3303, 2953, 2903, 2868, 1617, 1582, 1559, 1528, 1475, 1461, 1434, 1375, 1337, 1318, 1300, 1286, 1255, 1226, 1160, 1062, 1031, 1021 cm^−1^; Elemental Anal. Calcd for C_23_H_27_NOSe (413.13): C, 66.98; H, 6.60; N, 3.40 Found: C, 66.78; H, 6.52; N, 3.34.

*(+)-N-(1R,2R,3R,5S)-isopinocamphyl-2-(phenylselanyl)benzamide***23b** Yield: 54%; mp 128–130 °C; [α${]}_{D}^{20}$ = +14.35 (c = 4.43, CHCl_3_); ^1^H NMR (400 MHz, CDCl_3_) 0.89 (d, *J* = 10.0 Hz, 1H), 1.11 (s, 3H, CH_3_), 1.21 (d, *J* = 7.2 Hz, 3H, CH_3_), 1.26 (s, 3H, CH_3_), 1.63–1.69 (m, 1H), 1.86–1.93 (m, 2H), 1.99–2.06 (m, 1H), 2.42–2.48 (m, 1H), 2.71–2.78 (m, 1H), 4.48–4.56 (m, 1H), 6.09 (d, *J* = 7.2 Hz, 1H, NH), 7.09–7.15 (m, 1H_ar_), 7.16–7.23 (m, 2H_ar_), 7.34–7.41 (m 3H_ar_), 7.53–7.57 (m, 1H_ar_), 7.59–7.64 (m, 2H_ar_); ^13^C NMR (75.5 MHz, CDCl_3_) *δ* = 20.88 (CH_3_), 23.38 (CH_3_), 28.02 (CH_3_), 35.25 (CH_2_), 37.08 (CH_2_), 38.45 (C), 41.59 (CH), 46.21 (CH), 47.81 (CH), 48.48 (CH), 125.82 (CH_ar_), 127.54 (CH_ar_), 128.40 (CH_ar_), 129.58 (2 × CH_ar_), 130.08 (C_ar_), 130.77 (CH_ar_), 131.27 (CH_ar_), 134.33 (C_ar_), 135.19 (C_ar_), 135.73 (2 × CH_ar_), 167.74 (C=O); ^77^Se NMR (76 MHz, CDCl_3_) δ = 431.86 ppm; IR: 3311, 3055, 2958, 2908, 2869, 1623, 1584, 1563, 1531, 1476, 1454, 1434, 1376, 1351, 1335, 1311, 1298, 1285, 1258, 1226, 1163, 1092, 1064, 1031, 1021, 1000 cm^−1^; Elemental Anal. Calcd for C_23_H_27_NOSe (413.13): C, 66.98; H, 6.60; N, 3.40 Found: C, 66.68; H, 6.70; N, 3.51.

### 3.3. Antioxidant Activity Assay

To a solution of compounds **7b**–**23b** (0.015 mmol) and dithiothreitol DTT^red^ (0.15 mmol) in 1.1 mL of CD_3_OD, 30% H_2_O_2_ (0.15 mmol) was added. ^1^H NMR spectra were measured right after the addition of hydrogen peroxide, and then in specific time intervals. The concentration of the substrate was determined according to the changes in the integration on the ^1^H NMR spectra [19].

### 3.4. MTT Viability Assay

The MTT (3-(4,5-dimethyldiazol-2-yl)-2,5 diphenyl tetrazolium bromide) assay, which measures activity of cellular dehydrogenases, was based on the method of Mosmann [24]. Briefly, cells were seeded into 96-well plates (about 1.5 × 10^4^ cells per well, in 100 µL) and then left to adhere and grow for 24 h. Subsequently, 100 µL of the tested compounds in the medium was added to a final concentration of 0–250 μM, for 24 h, followed by the addition of 100 µL MTT, 3 mg/mL in PBS, for the next 3 h. After the incubation, the medium was removed. Remaining insoluble formazan crystals were dissolved in 100 µL DMSO. The absorbance of the blue formazan product was measured at 570 nm in the plate reader spectrophotometer Infinite M200 (Tecan, Grödig, Austria) and compared with the control (untreated cells). All experiments were performed three times in triplicate. The concentration of tested compounds required to inhibit cell viability by 50% (IC_50_) was calculated using Microsoft Excel software for semi-log curve fitting with linear regression analysis.

## 4. Conclusions

Herein, we have presented the synthesis of a new group of GPx-mimics, unsymmetrical phenyl selenides, functionalized on one of the phenyl rings with a *N*-substituted *o*-amido group. The obtained compounds were diversified on the nitrogen atom with aromatic and aliphatic groups, including chiral terpene scaffolds. The molecules were designed as *N*-substituted benzisoselenazol-3(2*H*)-ones (“ebselen-like” therapeutics), whereas the Se-N bond was cleaved and a -SePh group was installed with a secondary amide moiety -C(O)NHR functionalization. The compounds were further tested as potential antioxidants and anticancer agents. The highest peroxide scavenging activity, significantly higher than for ebselen, was found for *N*-(3-methylbutyl)-2-(phenylselanyl)benzamide. The significant cytotoxicity was observed for derivatives with terpenyl carane and *p*-menthane skeletons. The performed in vitro studies revealed that, however, the antioxidant potential was not improved in most cases in comparison to the results obtained for corresponding benzisoselenazol-3(2*H*)-ones; the modification was beneficial for a higher antiproliferative effect towards MCF-7 and HL-60 cancer cells. It can therefore be concluded that an additional aromatic ring attached to the selenium atom may have a positive influence on the enhanced cytotoxity of the selected phenylselenides.

## Figures and Tables

**Table 1:** Cytotoxic activity of *N*-terpene derivatives and the corresponding benzisoselenazol-3(2*H*)-ones.

Structure	MCF-7 IC_50_ [µM]	HL-60 IC_50_ [µM]	Structure	MCF-7 IC_50_ [µM]	HL-60 IC_50_ [µM]
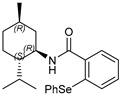 **18b**	36.1 ± 0.6	10.7 ± 0.6	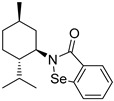 **33**	11.9 ± 0.2	62 ± 2.0
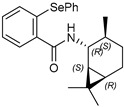 **19b**	16.3 ± 0.3	16.3 ± 0.2	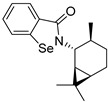 **34**	24.3 ± 2.4	203 ± 2.0

## Supplementary Materials

The following are available online, results of the antioxidant activity evaluation; results of the antiproliferative activity evaluation, ^1^H- and ^13^C-NMR spectra of compounds **18a**–**23a**, and ^1^H, ^13^C and ^77^Se spectra of compounds **7b**–**23b**.
