# Supplementary material for: Phenylselanyl Group Incorporation for “Glutathione Peroxidase-Like” Activity Modulation
Source: Molecules. 2020 Jul 24;25(15):3354. doi: 10.3390/molecules25153354 (PMC7435675; doi:10.3390/molecules25153354)
Supplement: Supplementary file 1 [file molecules-25-03354-s001.pdf]

Supporting Information

Phenylselanyl group incorporation for "glutathione peroxidase-like" activity modulation

Magdalena Obieziurska-Fabisiak[a], Agata J. Pacuła[a], Lucia Capoccia[b], Joanna Drogosz-Stachowicz[c], Anna Janecka[c], Claudio Santi[b] and Jacek Ścianowski[a*]

[a]Department of Organic Chemistry, Faculty of Chemistry, Nicolaus Copernicus University, 7 Gagarin Street, 87-100 Torun, Poland

[b]Dipartimento di Scienze Farmaceutiche, Universita di Perugia, Via del Liceo 1, 06134, Perugia, Italy

[c]Department of Biomolecular Chemistry, Faculty of Medicine, Medical University of Lodz, Mazowiecka 6/8, 92-215 Lodz, Poland

*E-mail: jsch@umk.pl

Index of content

I. Results of the antioxidant activity evaluation
II. Results of the antiproliferative activity evaluation
III. NMR spectra
IV. References

I. Results of the antioxidant activity evaluation

To a solution of compounds **7b**–**23b** (0.015mmol) and dithiothreitol DTT^{red} (0.15mmol) in 1.1 mL of CD_3OD, 30% H_2O_2 (0.15 mmol) was added. ^1H NMR spectra were measured right after addition of hydrogen peroxide and then in specific time intervals. The concentration of the substrate was determined according to the changes in the integration on the ^1H NMR spectra [1].

	**Remaining DTT^{red} [%]**				
**Catalyst [0.1 equiv.]**	**3 min**	**5 min**	**15 min**	**30 min**	**60 min**
**7b**	99	98	98	97	93
**8b**	97	96	94	91	80
**9b**	**57**	**39**	**16**	**4**	**0**
**10b**	**98**	**97**	**94**	**88**	**71**
**11b**	94	92	91	89	87
**12b**	99	98	97	95	92
**13b**	97	96	95	94	92
**14b**	96	95	93	92	89
**15b**	94	93	92	91	89
**16b**	96	95	94	93	92
**17b**	97	96	95	94	92
**18b**	96	95	93	92	90
**19b**	94	93	91	90	87
**20b**	95	94	92	91	87
**22b**	**90**	**87**	**83**	**75**	**60**
**Ebselen**	84	75	64	58	52

II. Results of the antiproliferative activity evaluation

The MTT (3-(4,5-dimethyldiazol-2-yl)-2,5 diphenyl tetrazolium bromide) assay, which measures activity of cellular dehydrogenases, was based on the method of Mosmann [2]. Briefly, cells were seeded into 96-well plates (about 1.5 × 104 cells per well, in 100 µL) and then left to adhere and grow for 24 h. Subsequently, 100 µL of the tested compounds in the medium were added to a final concentrationof0–250µM,andkeptfor48h,followedbytheadditionof100µLMTT,3mg/mLinPBS, for the next 3h.After the incubation, the medium was removed. Remaining in soluble formazan crystals were dissolved in 100µL DMSO. The absorbance of the blue formazan product was measured at 570nm in the plate reader spectrophotometer Infinite M200 (Tecan, Grödig, Austria) and compared with control (untreated cells). All experiments were performed three times in triplicate. The concentration of tested compounds required to inhibit cell viability by 50% (IC50) was calculated using Microsoft Excel software for semi-log curve fitting with linear regression analysis.

	**IC50 [uM]± SEM**			
	**HL-60**		**MCF-7**	
**7b**	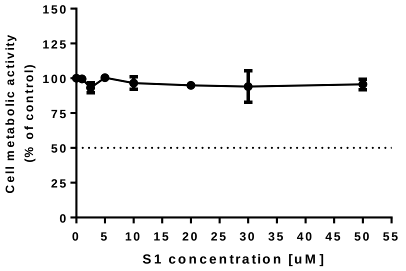	>50	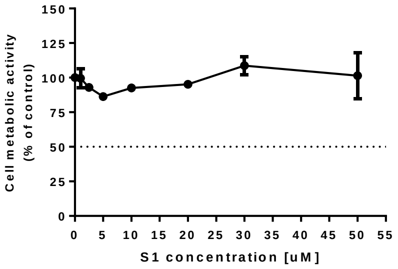	>50
**8b**	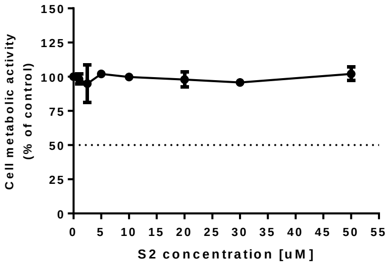	>50	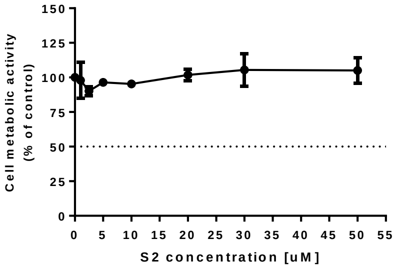	>50
**9b**	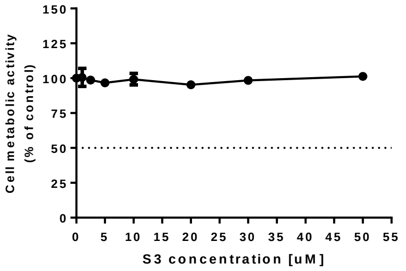	>50	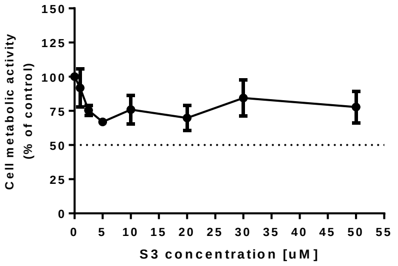	>50
**10b**	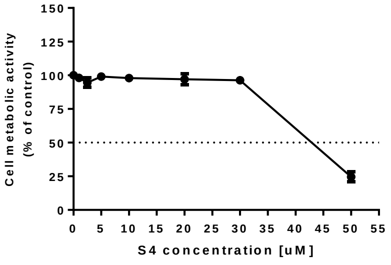	**42.85 ±0.20**	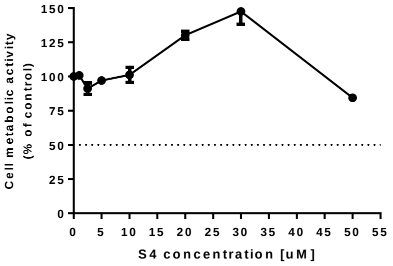	>50

**11b**	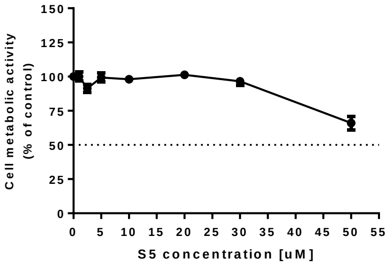	>50	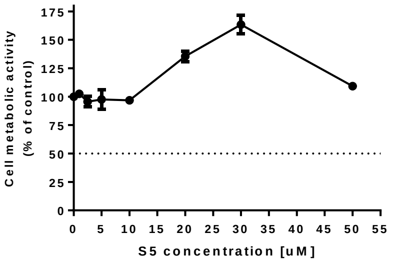	>50
**12b**	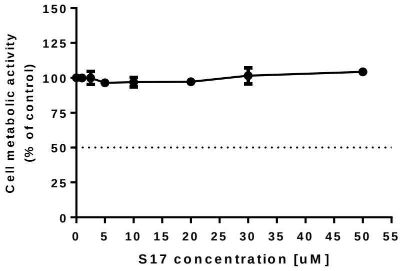	>50	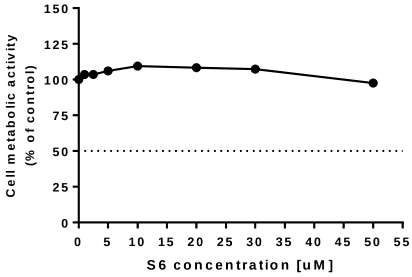	>50
**13b**	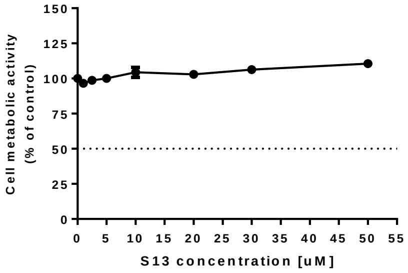	>50	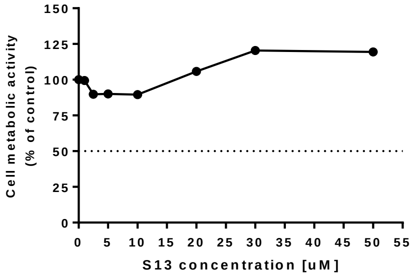	>50
**14b**	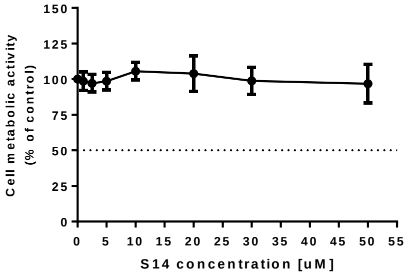	>50	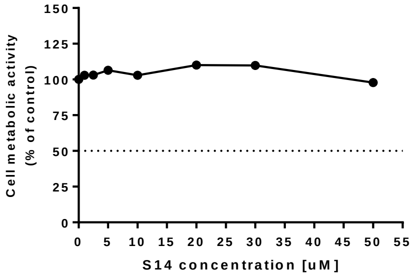	>50
**15b**	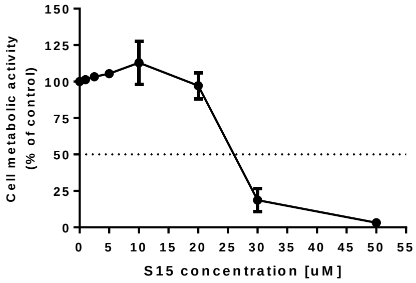	**26.00 ±0.57**	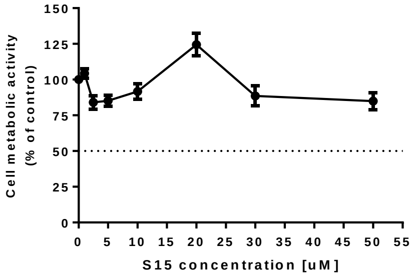	>50

**16b**	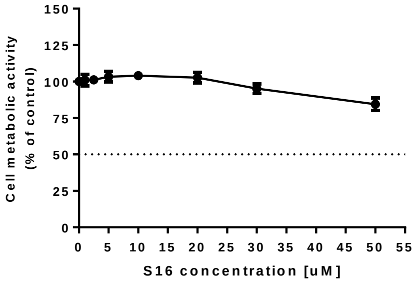	>50	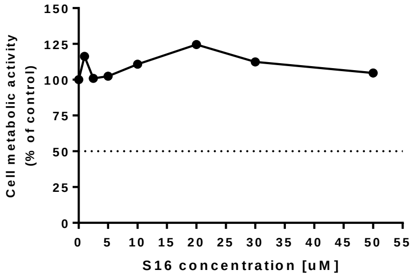	>50
**17b**	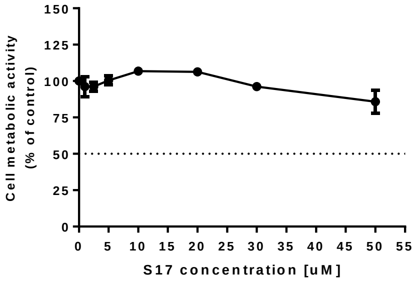	>50	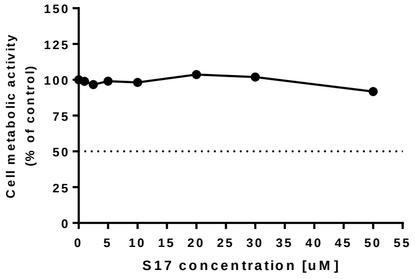	>50
**18b**	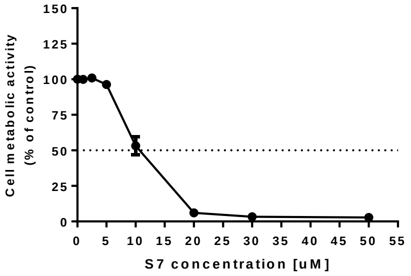	**10.67 ±0.60**	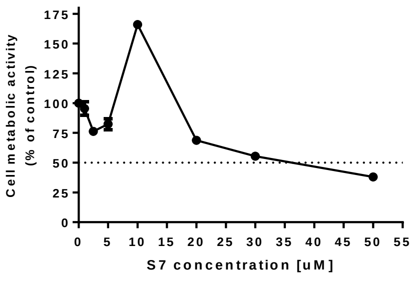	**36.10 ±0.65**
**19b**	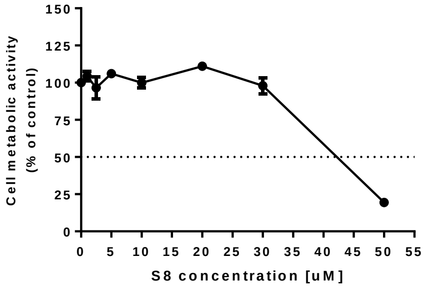	**42.10 ±0.33**	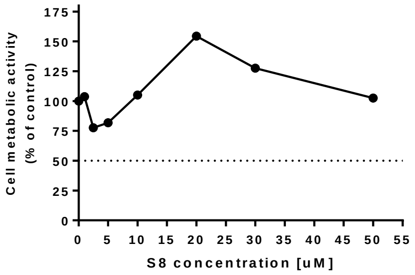	>50
**20b**	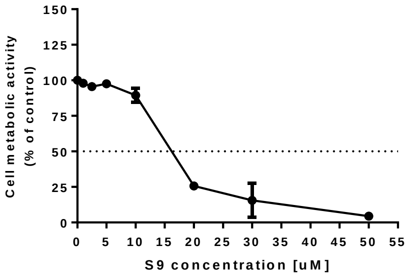	**16.30 ±0.16**	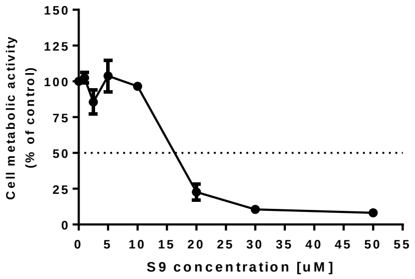	**16.35 ±0.29**

**21b**	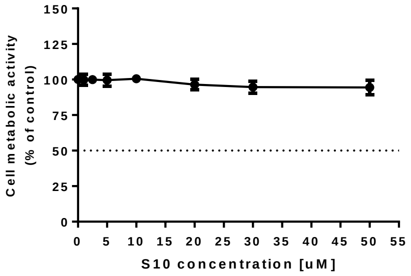	>50	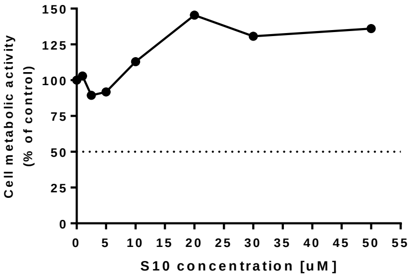	>50
**22b**	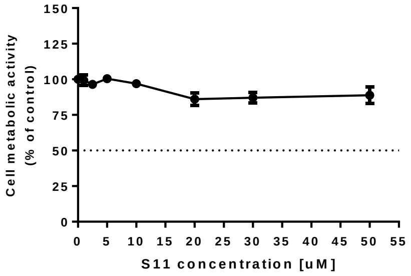	>50	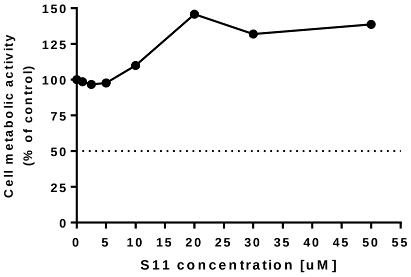	>50
**23b**	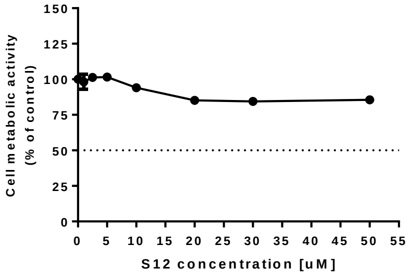	>50	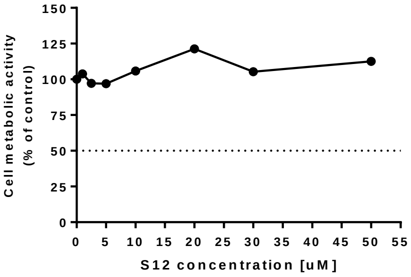	>50

III. NMR spectra

((−)-*N*-(1*R*,2*S*,5*R*)-menthyl)-*o*-iodobenzamide **18a**

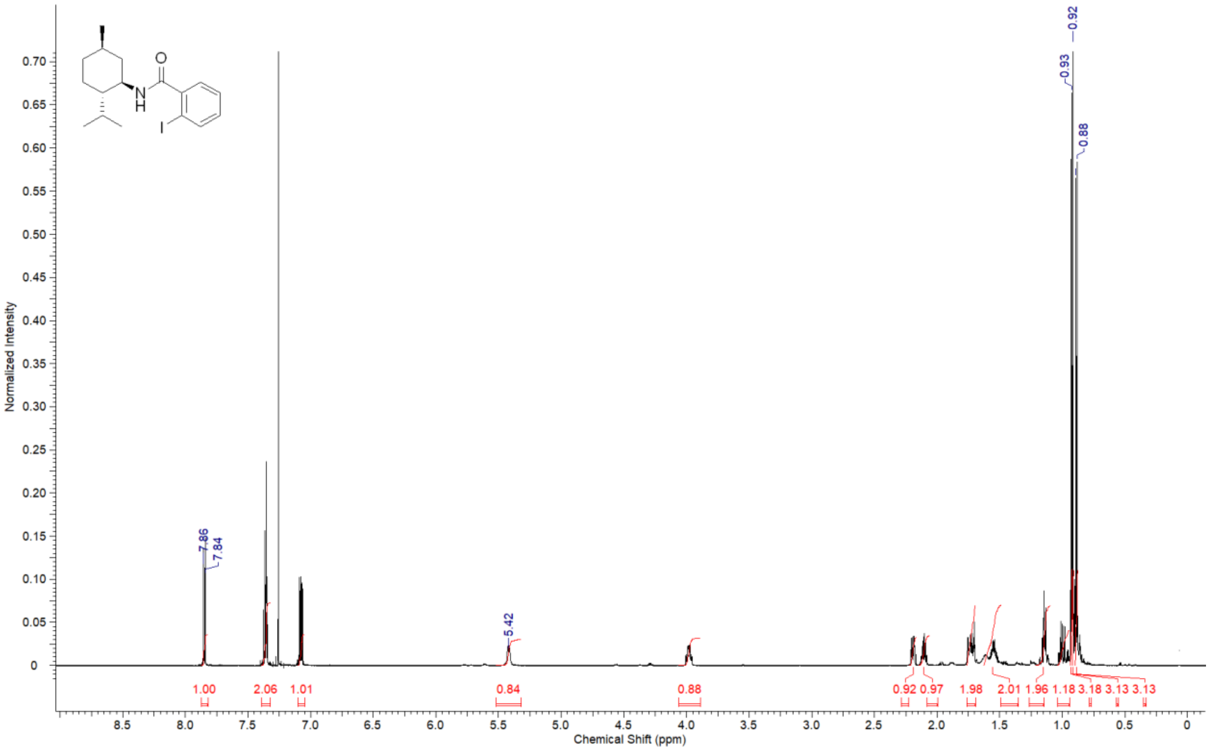

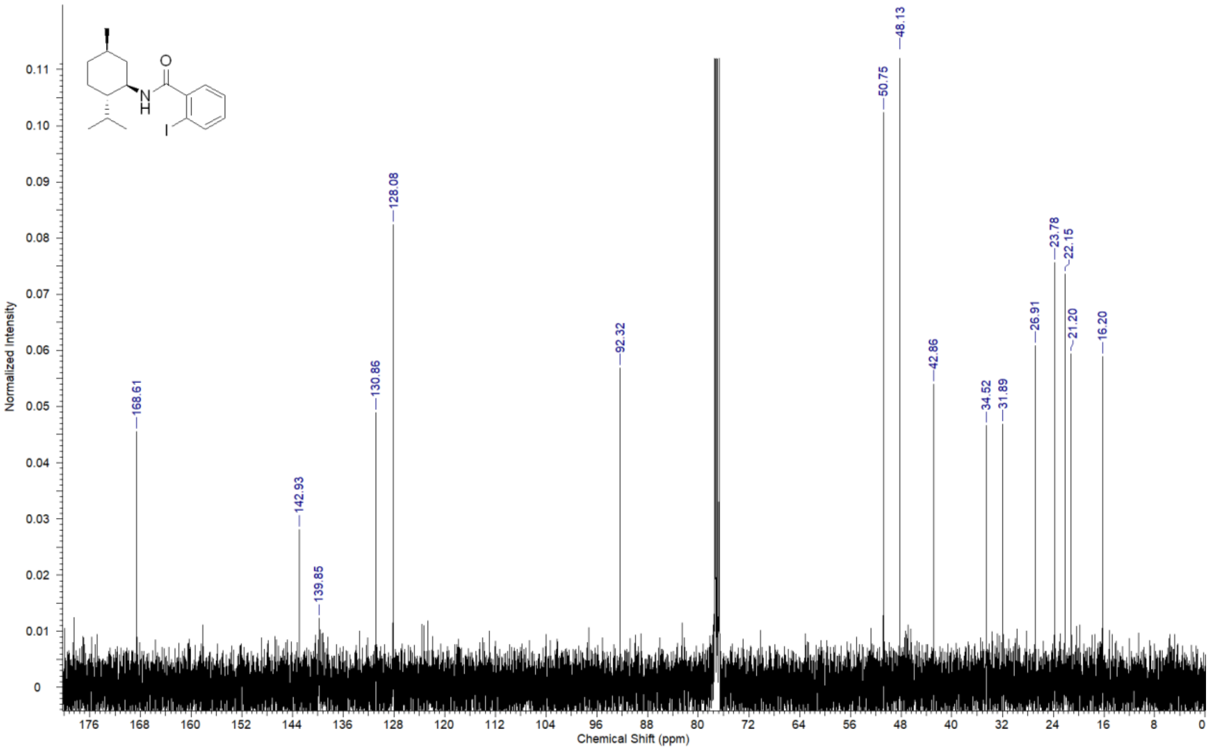

((-)-*N*-(1*S*,2*R*,3*S*,6*R*)-(2-caranyl))-*o*-iodobenzamide **19a**

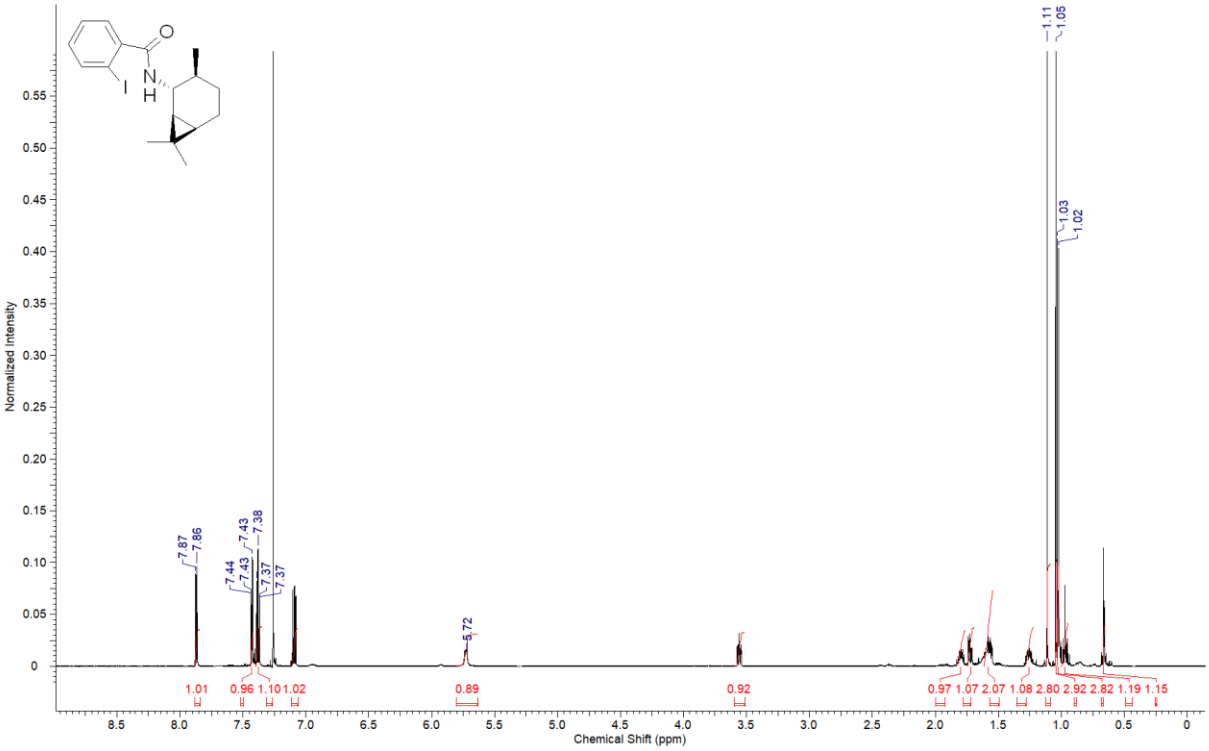

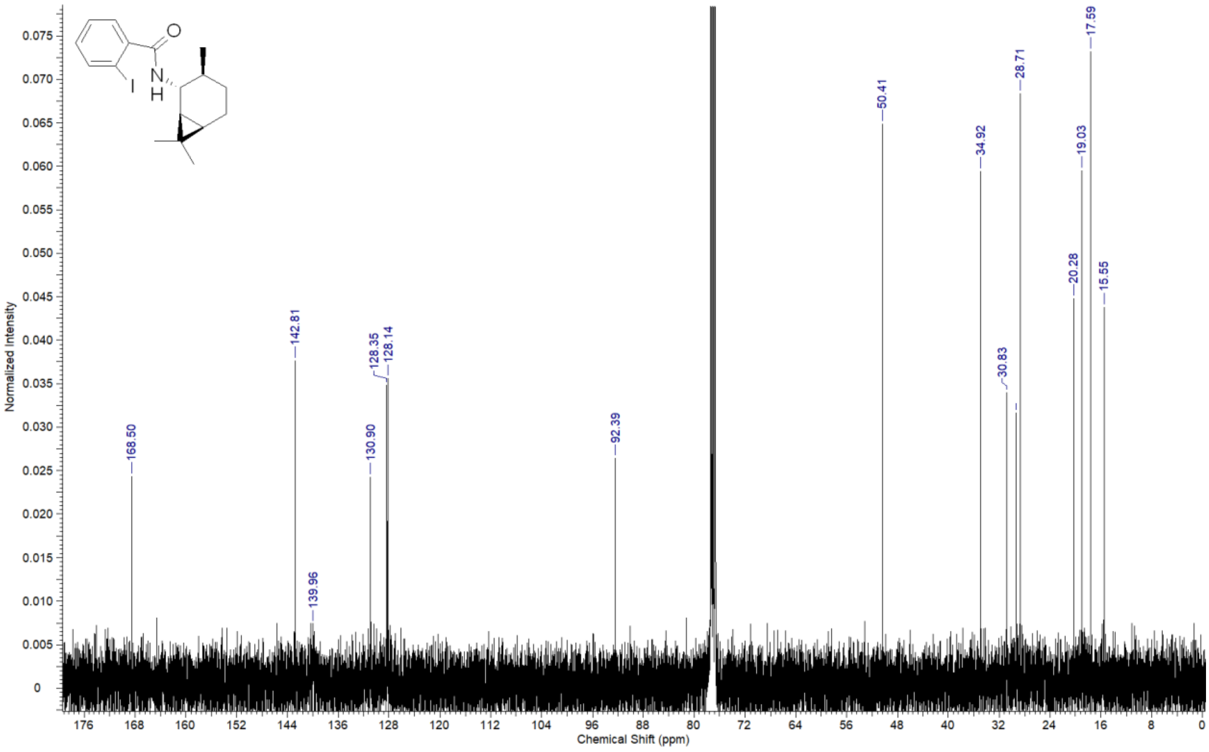

N-bornyl-*o*-iodobenzamide **20a**

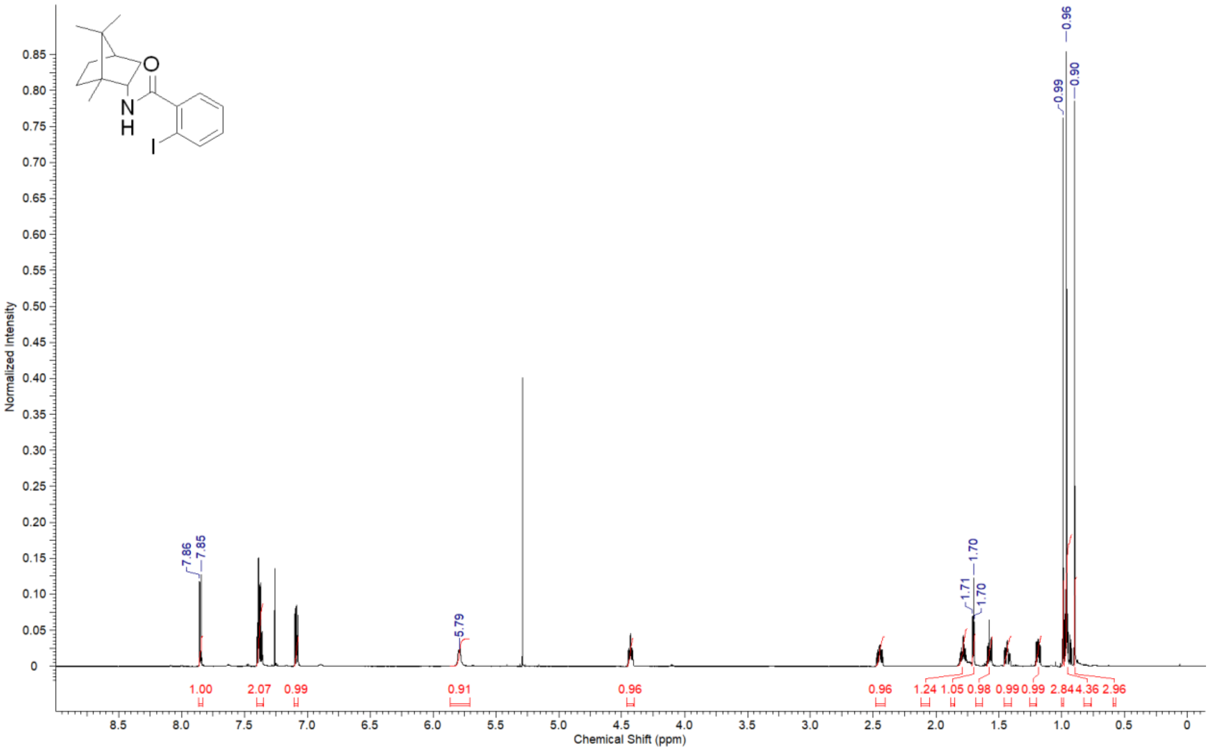

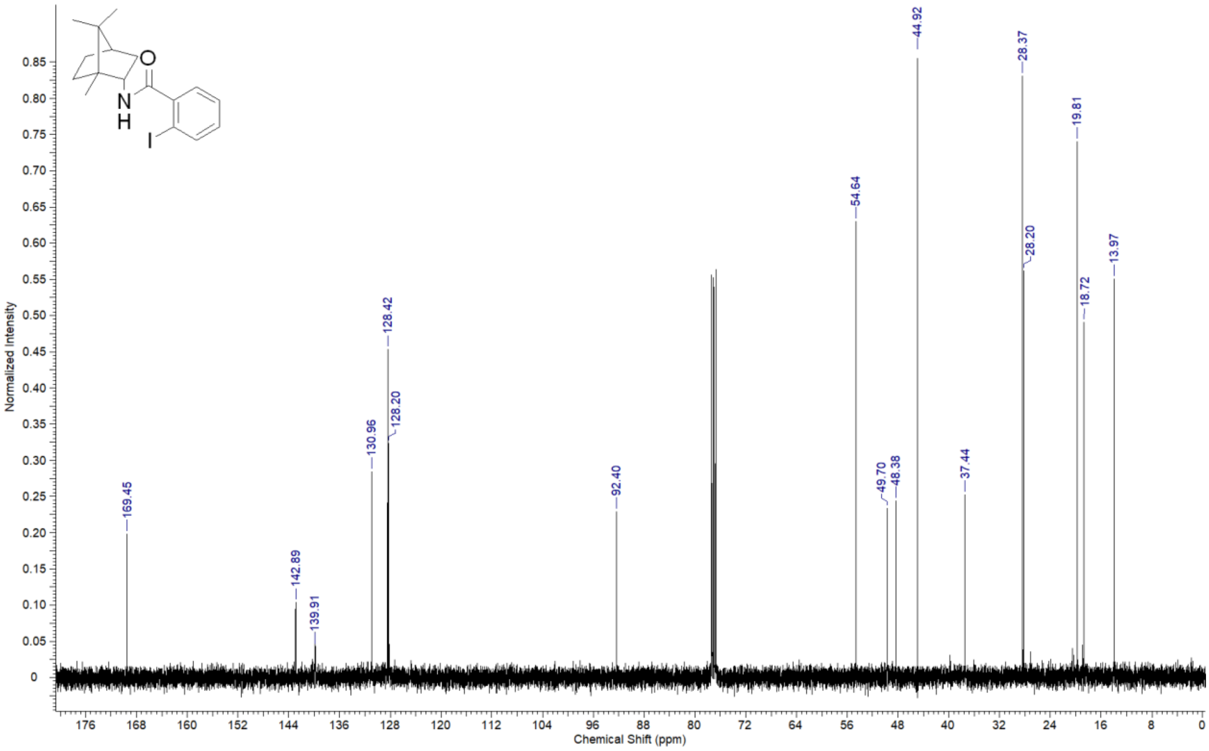

(-)-*N*-(1*S*,2*R*,5*S*)-myrtanyl-*o*-iodobenzamide **21a**

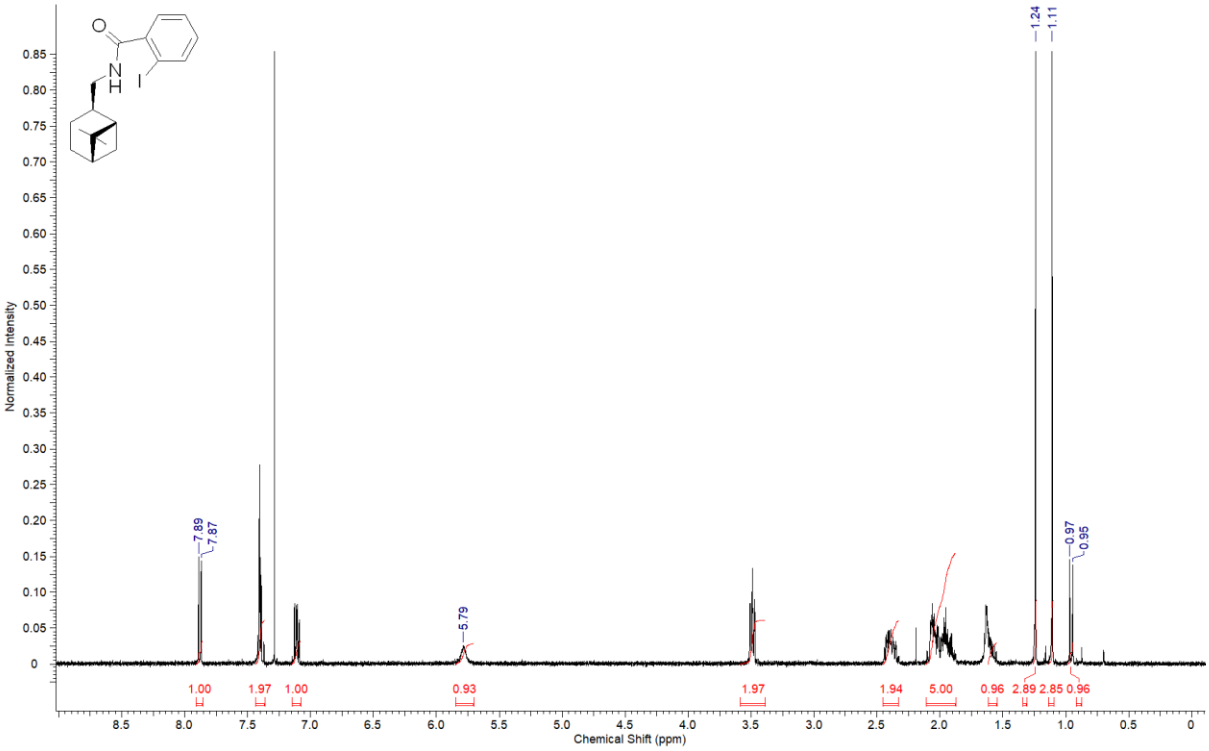

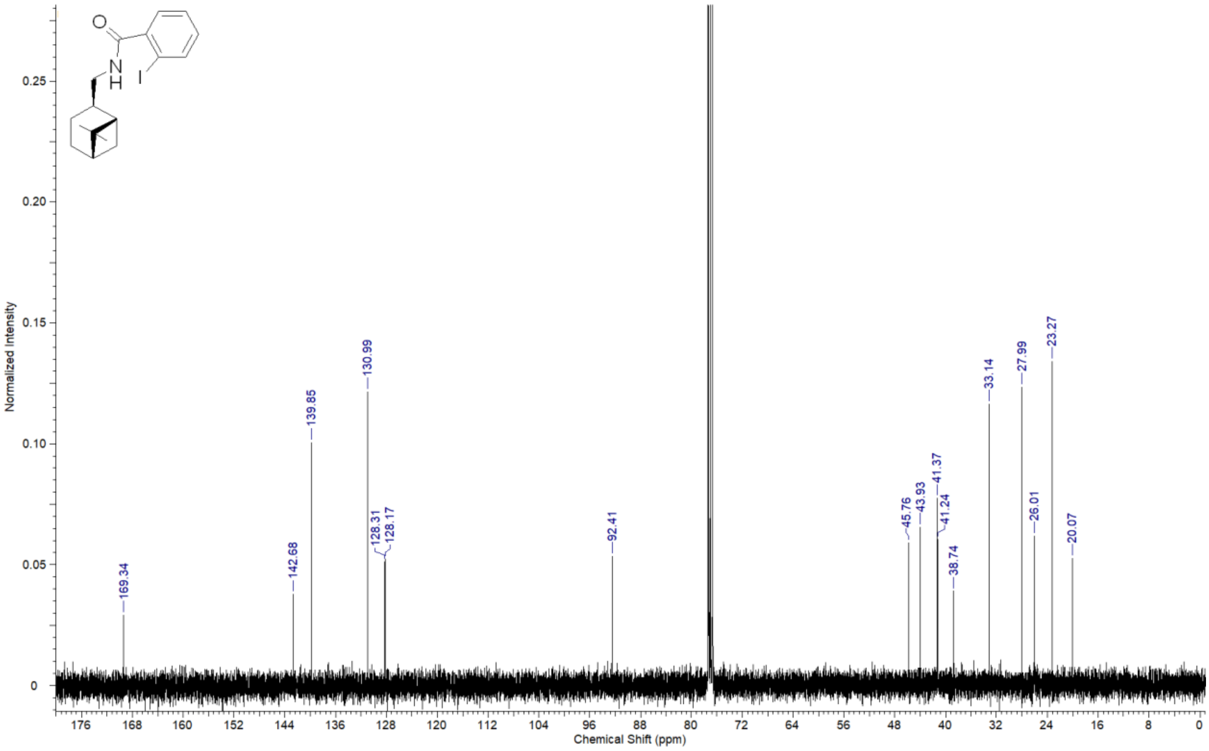

(-)-*N*-(1*R*,2*R*,3*R*,5*S*)-isopinocamphyl-*o*-iodobenzamide **22a**

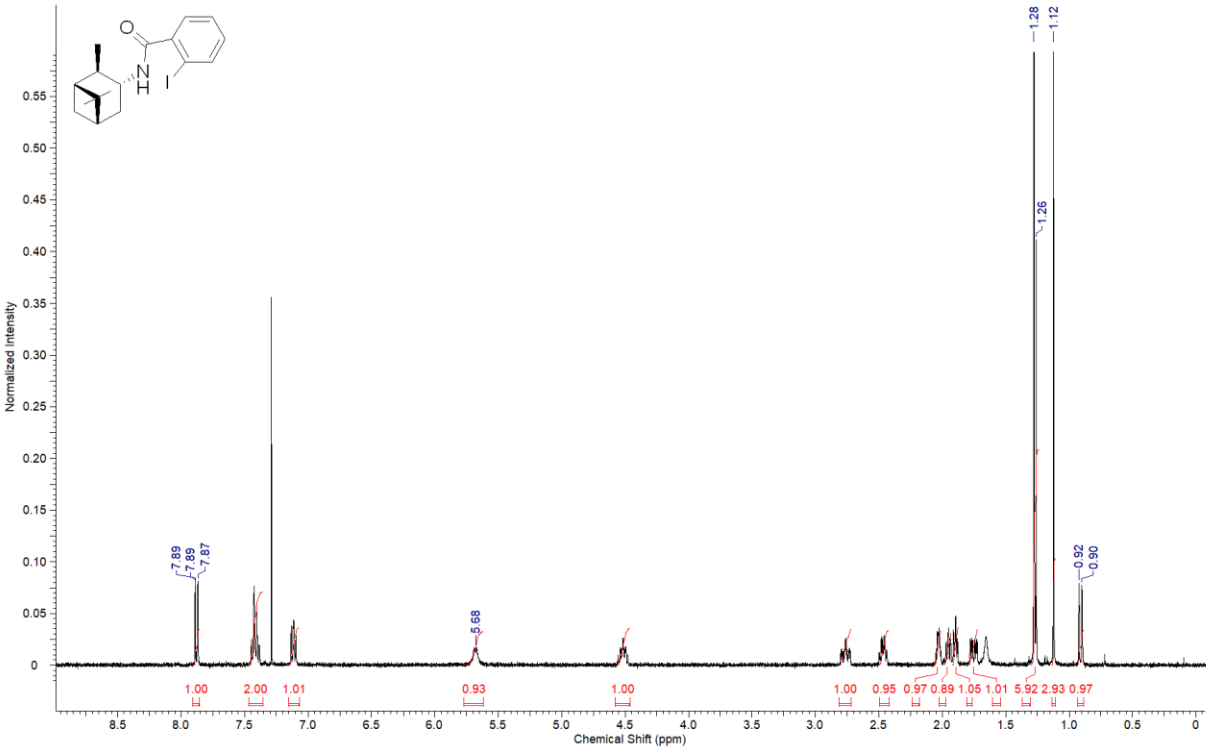

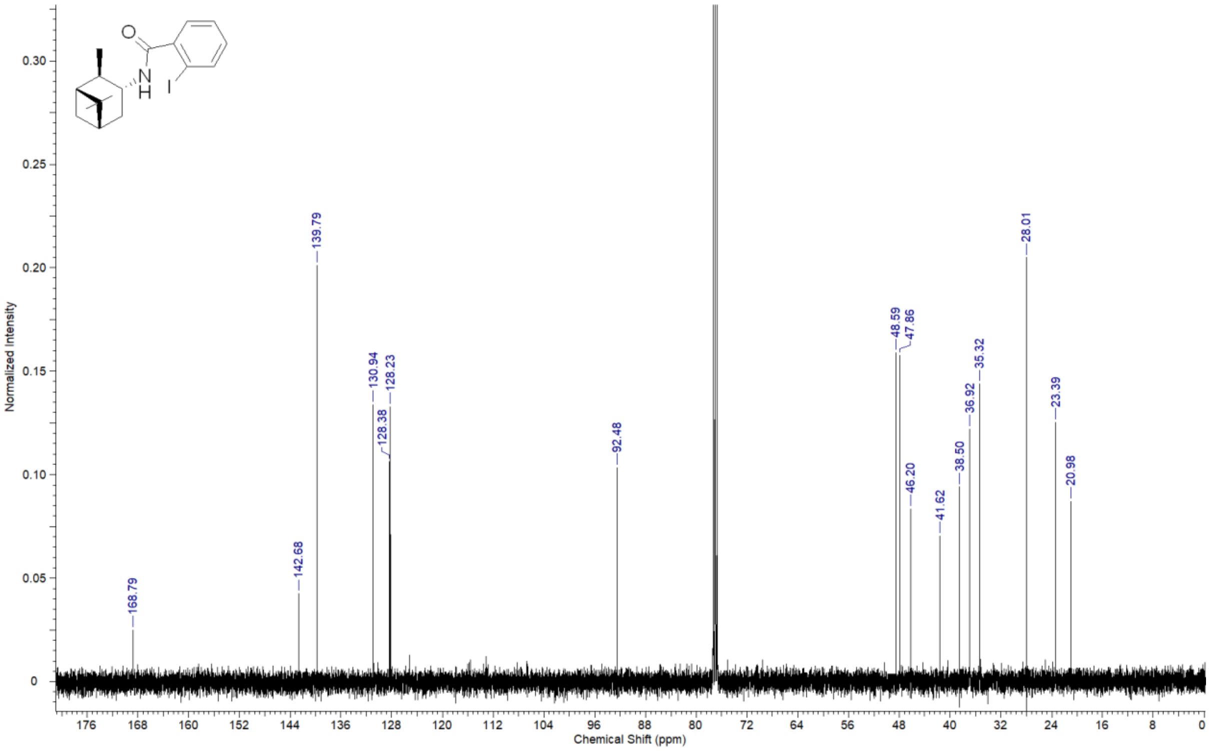

(+)-*N*-(1*R*,2*R*,3*R*,5*S*)-isopinocamphyl-*o*-iodobenzamide **23a**

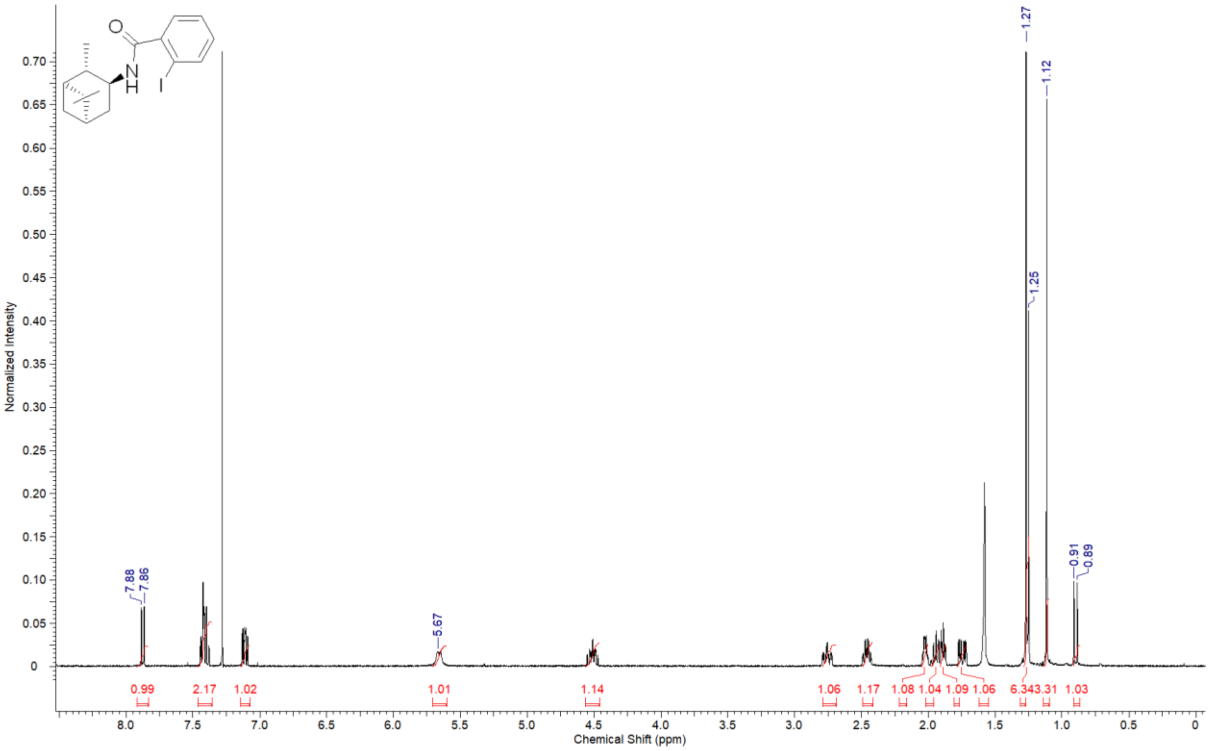

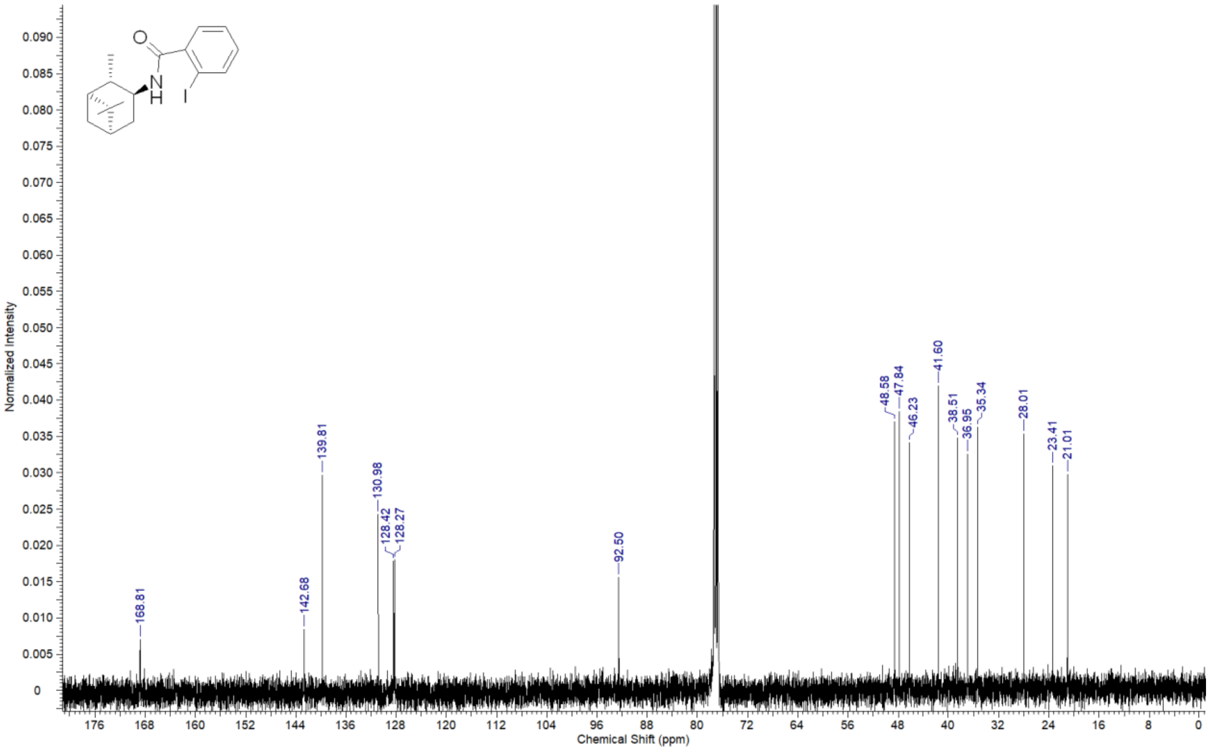

N-ethyl-2-(phenylselanyl)benzamide **7b**

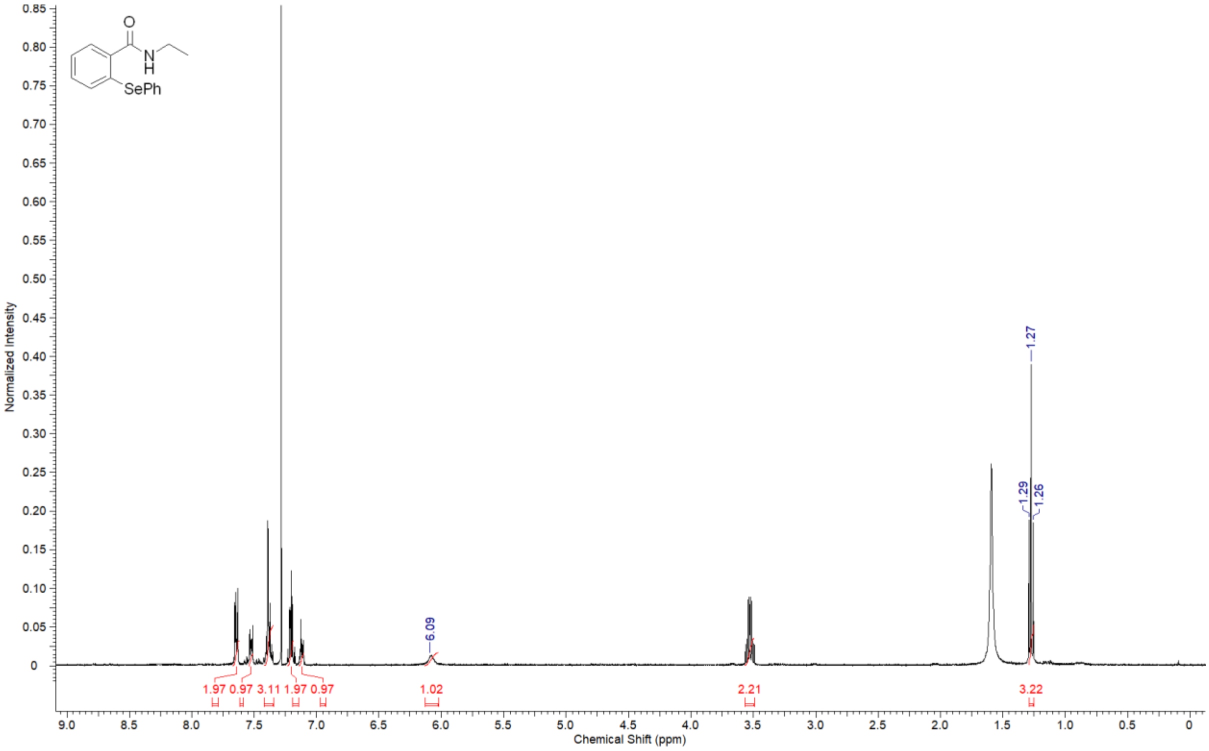

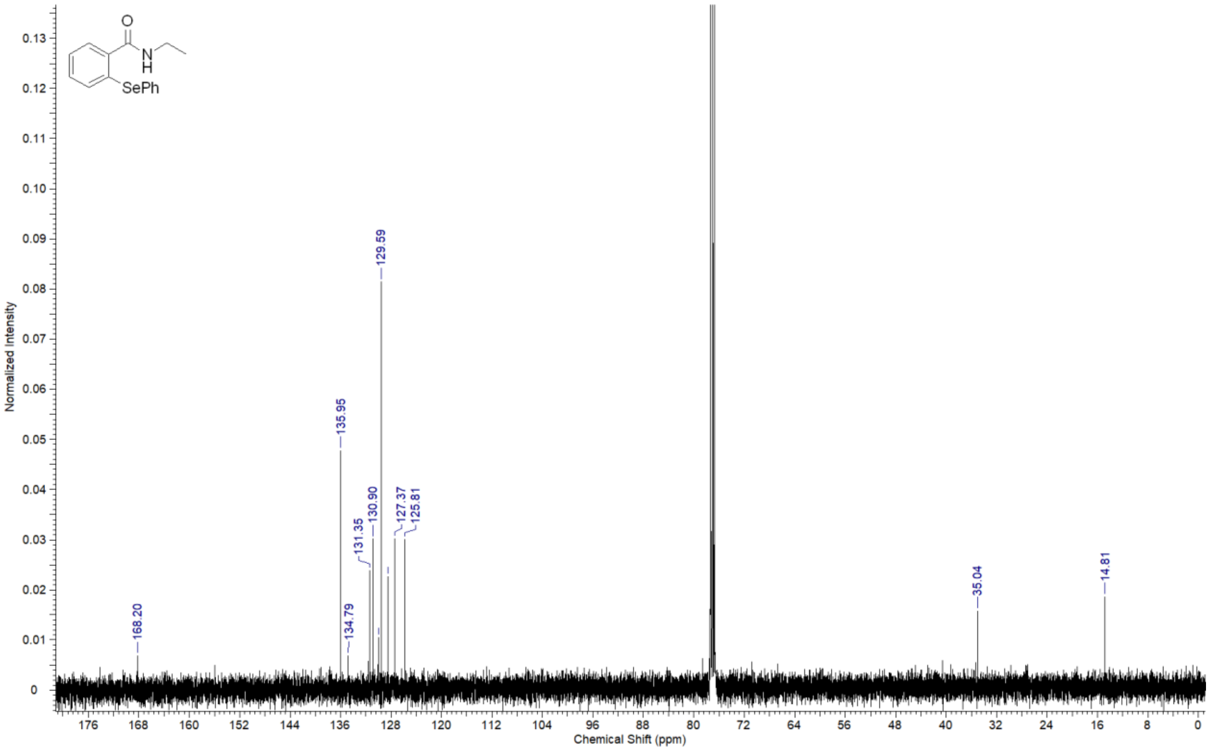

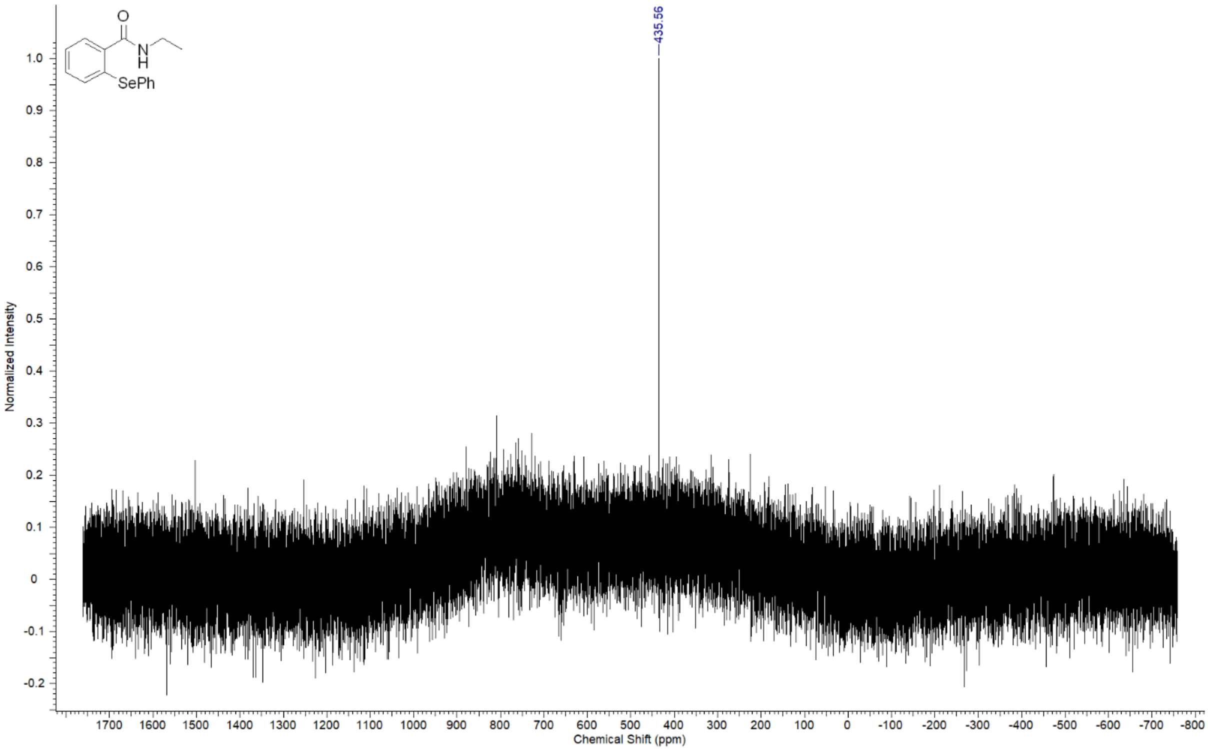

N-propyl-2-(phenylselanyl)benzamide **8b**

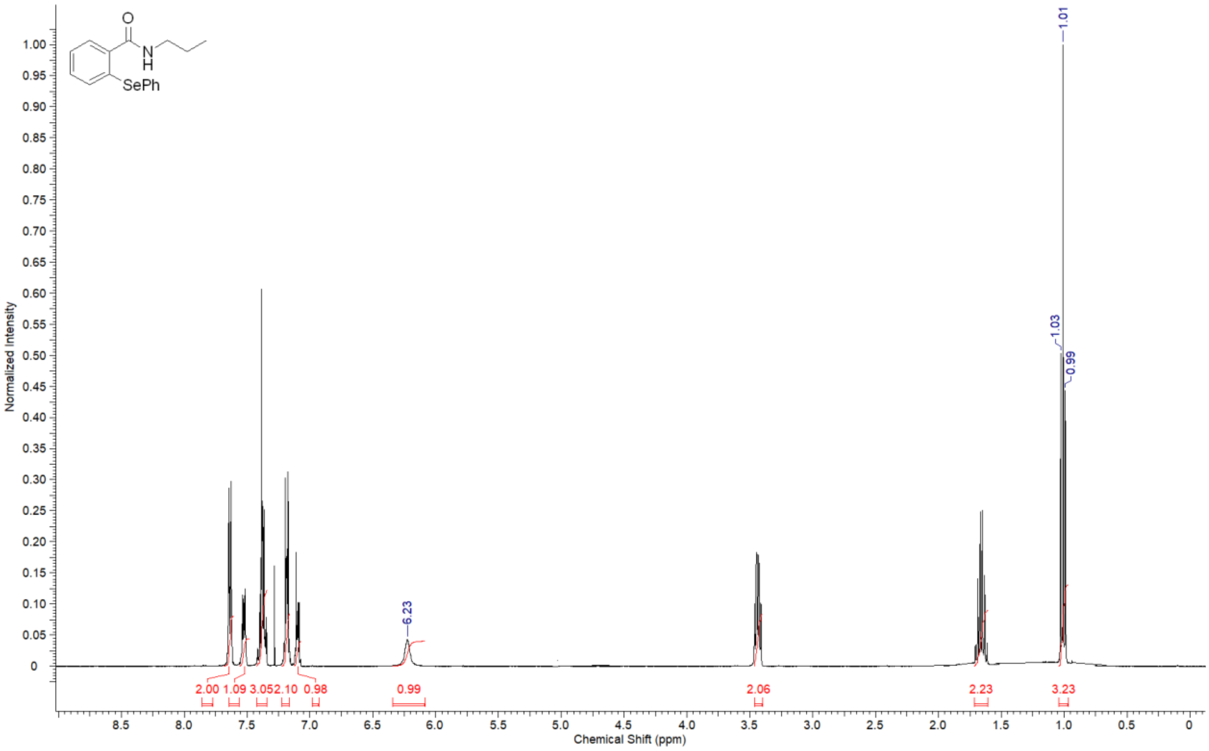

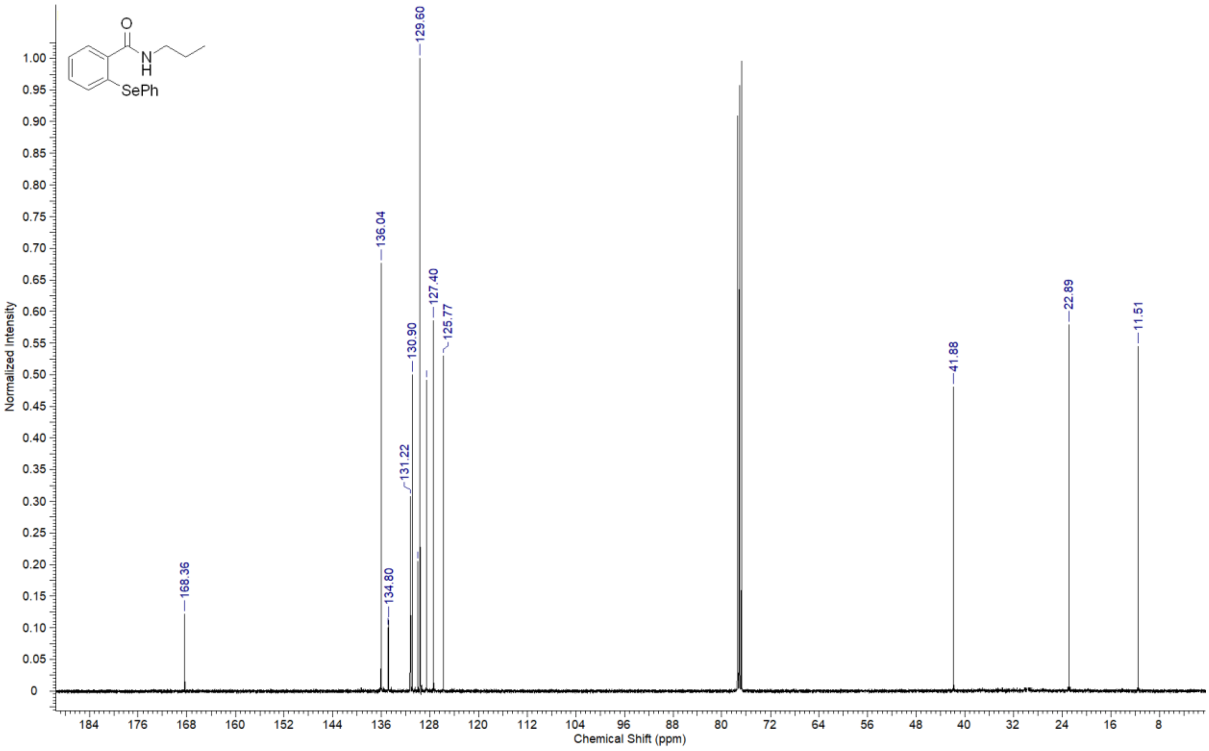

SePh
O
N
H
168.36
136.04
134.80
131.22
130.90
129.60
127.40
125.77
41.88
22.89
11.51
Normalized Intensity
Chemical Shift (ppm)

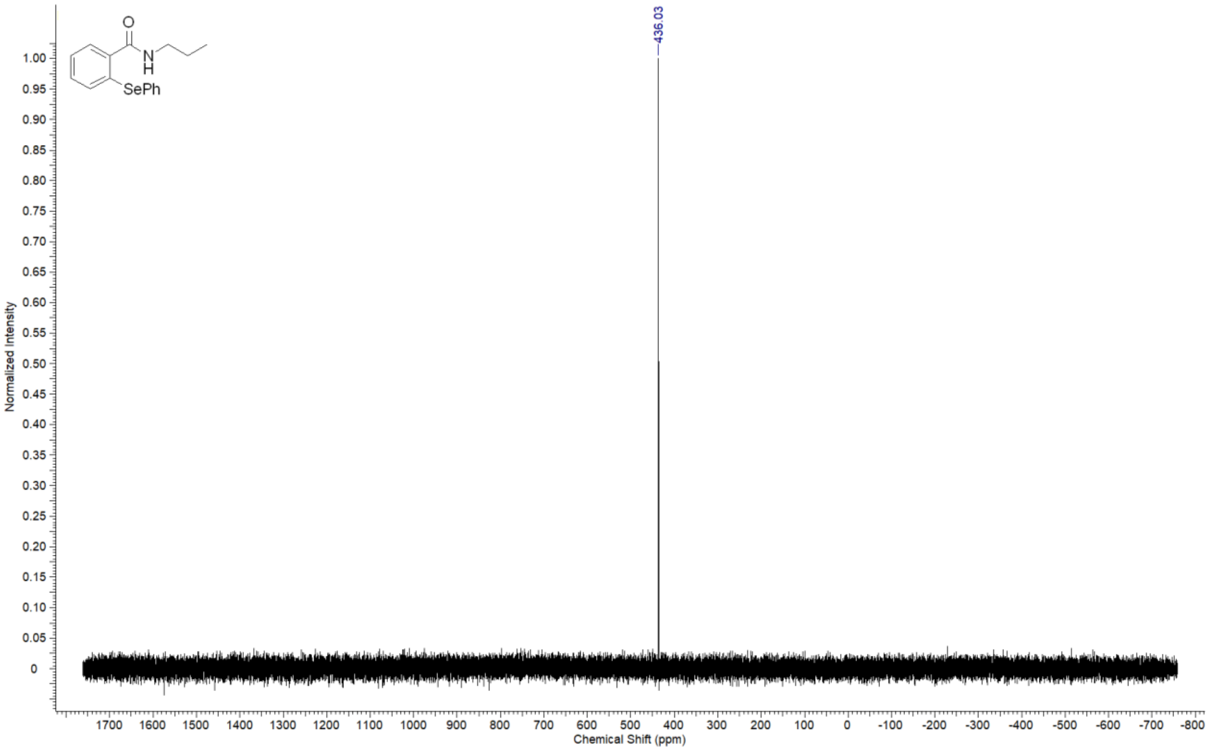

SePh
O
N
H
436.03
Normalized Intensity
Chemical Shift (ppm)

N-butyl-2-(phenylselanyl)benzamide **9b**

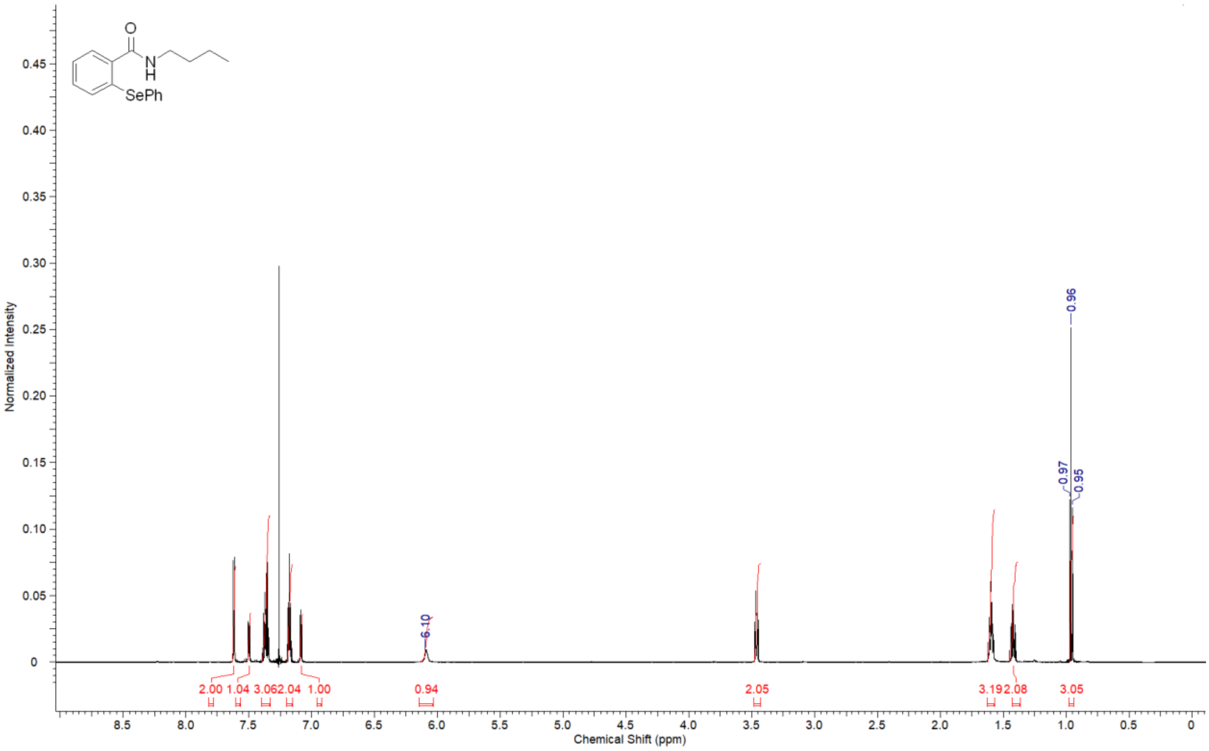

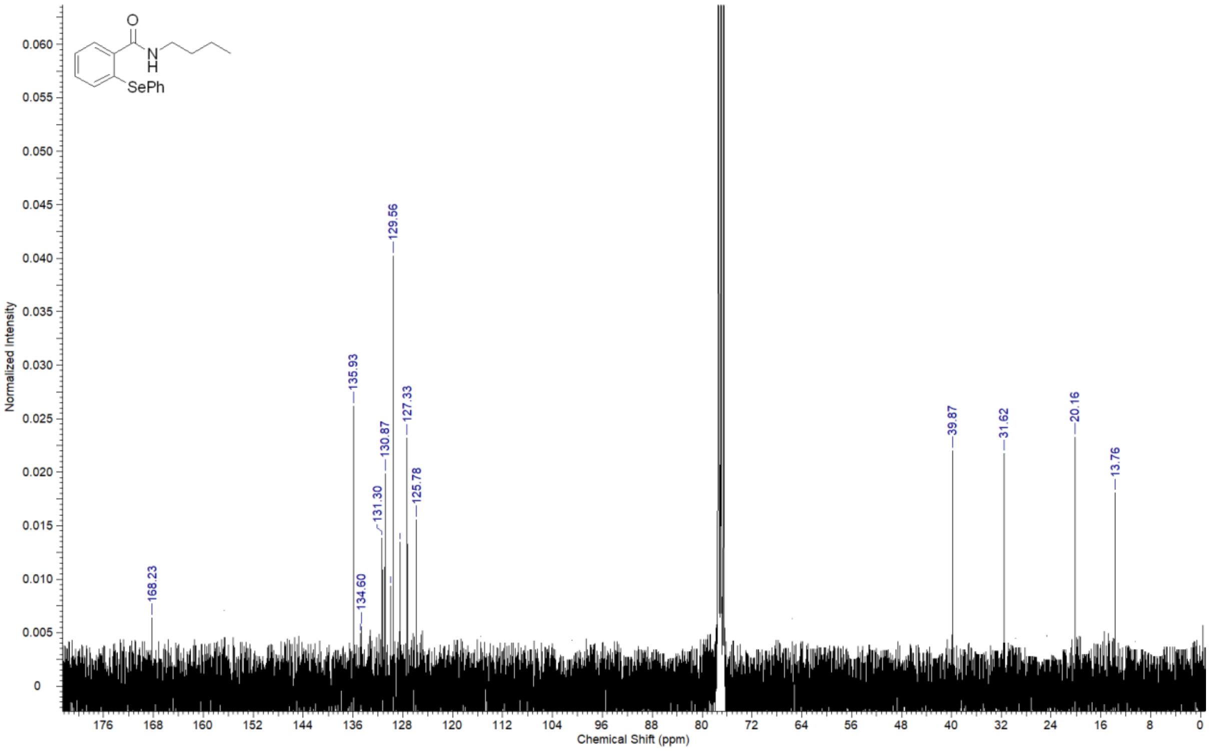

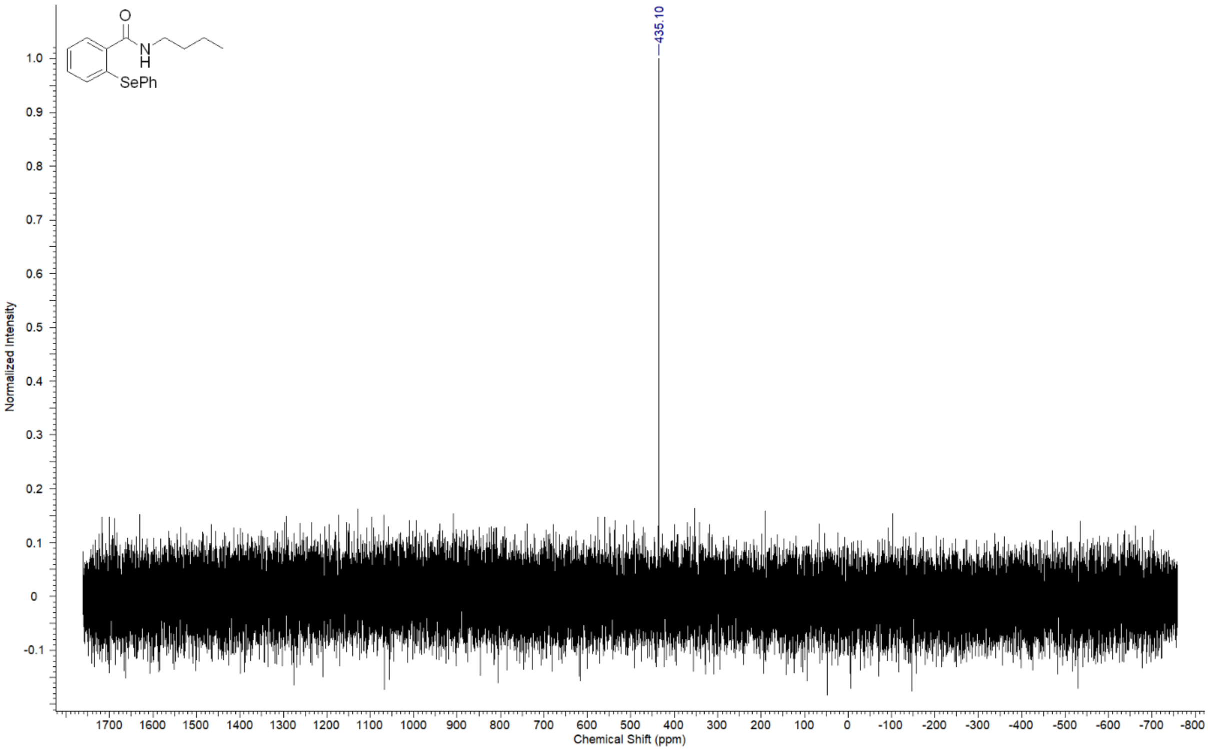

N-(3-methylbutyl)-2-(phenylselanyl)benzamide **10b**

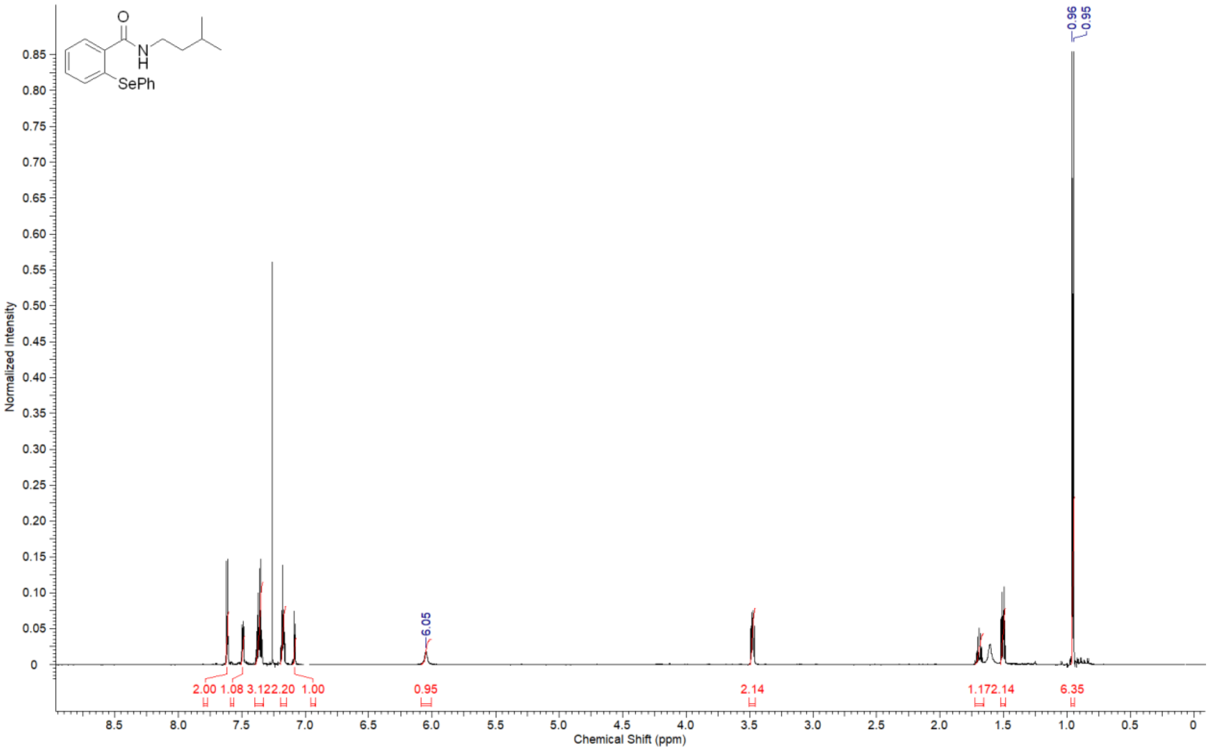

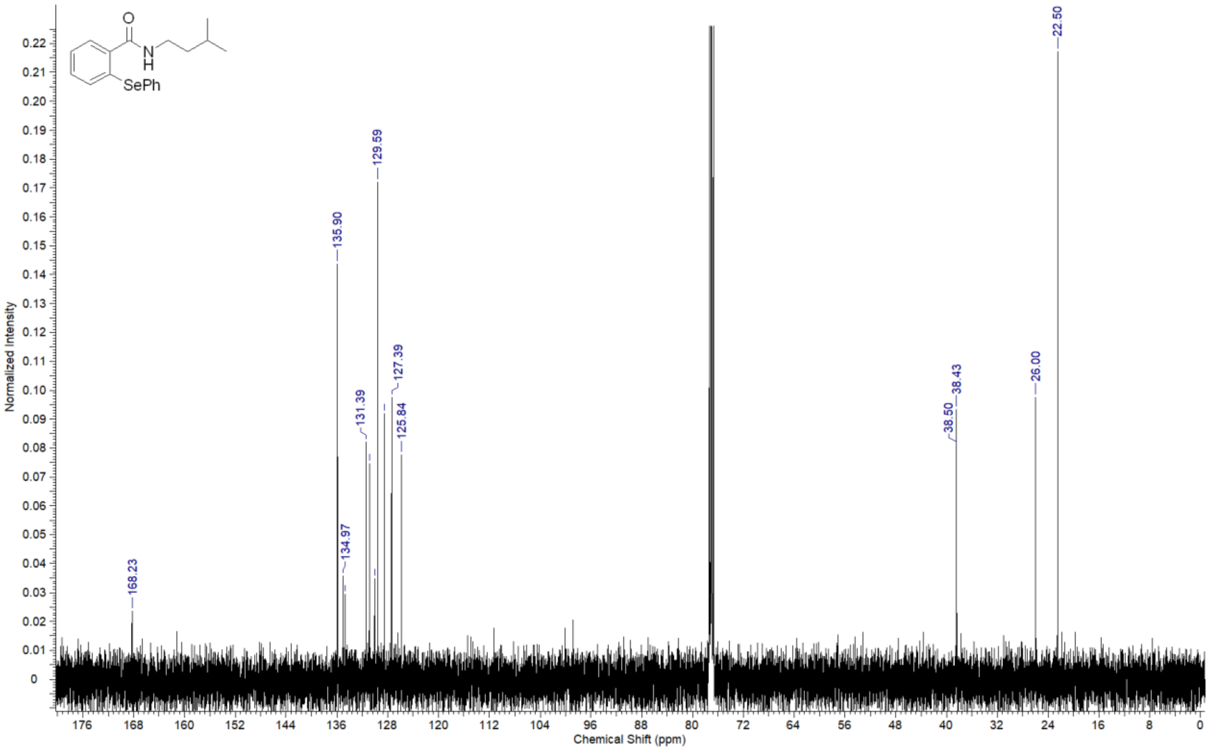

SePh
Normalized Intensity
Chemical Shift (ppm)
168.23
135.90
134.97
131.39
129.59
127.39
125.84
38.50
38.43
26.00
22.50

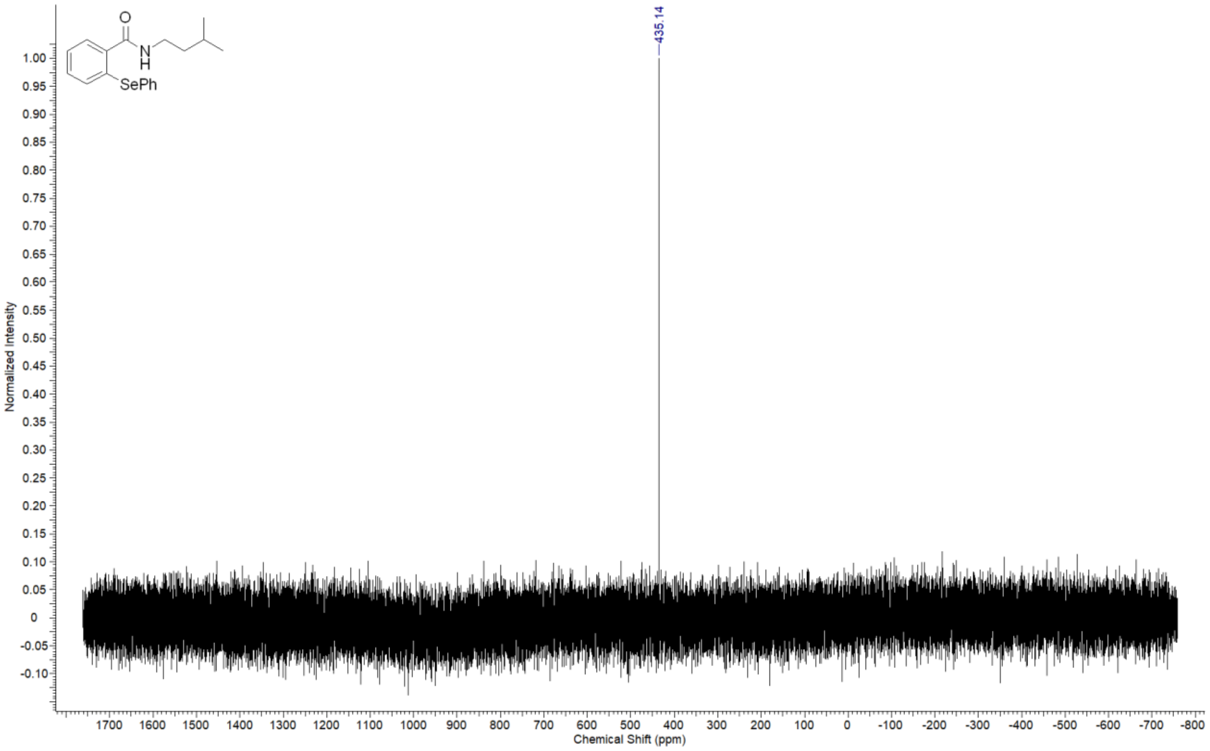

SePh
Normalized Intensity
Chemical Shift (ppm)
435.14

N-hexyl-2-(phenylselanyl)benzamide **11b**

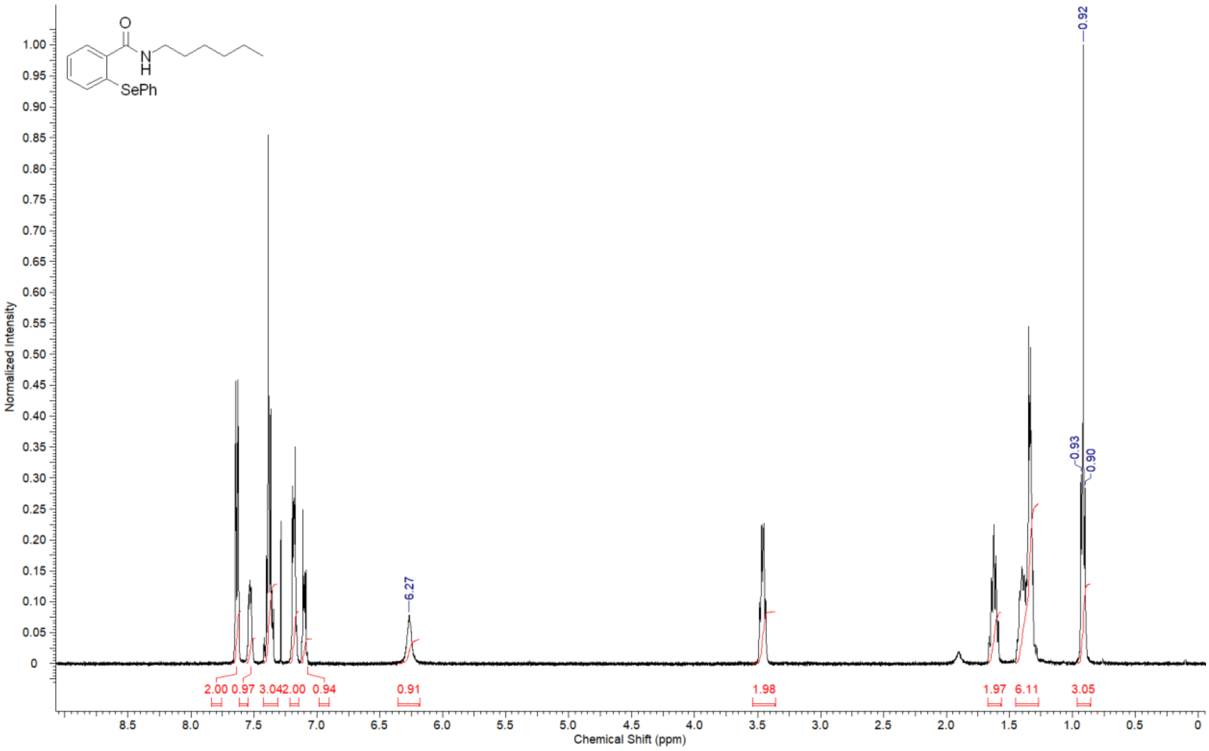

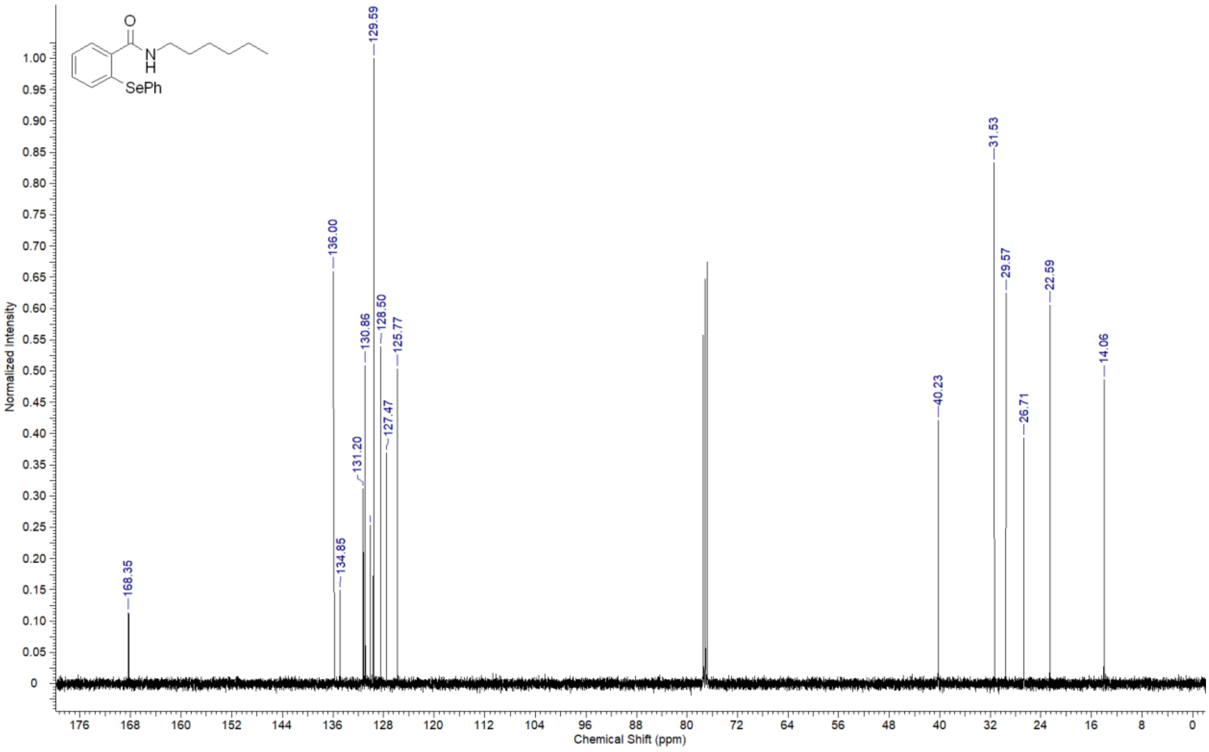

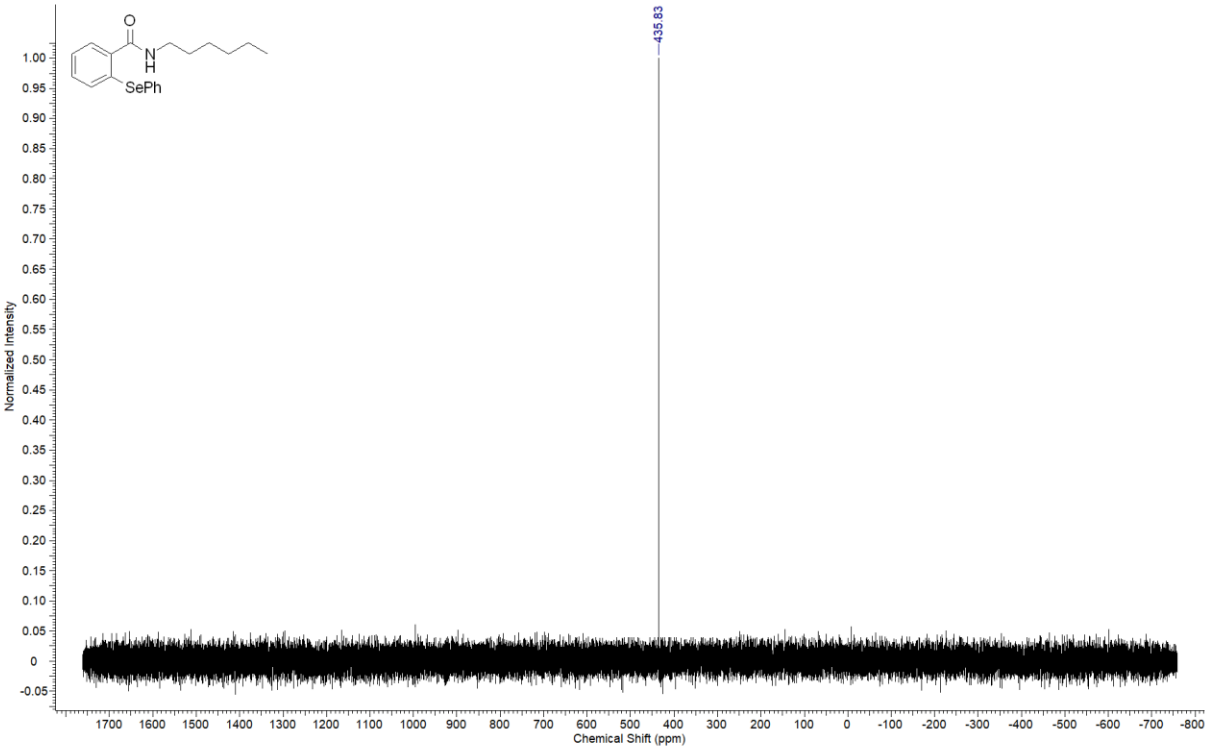

N-cyklohexyl-2-(phenylselanyl)benzamide **12b**

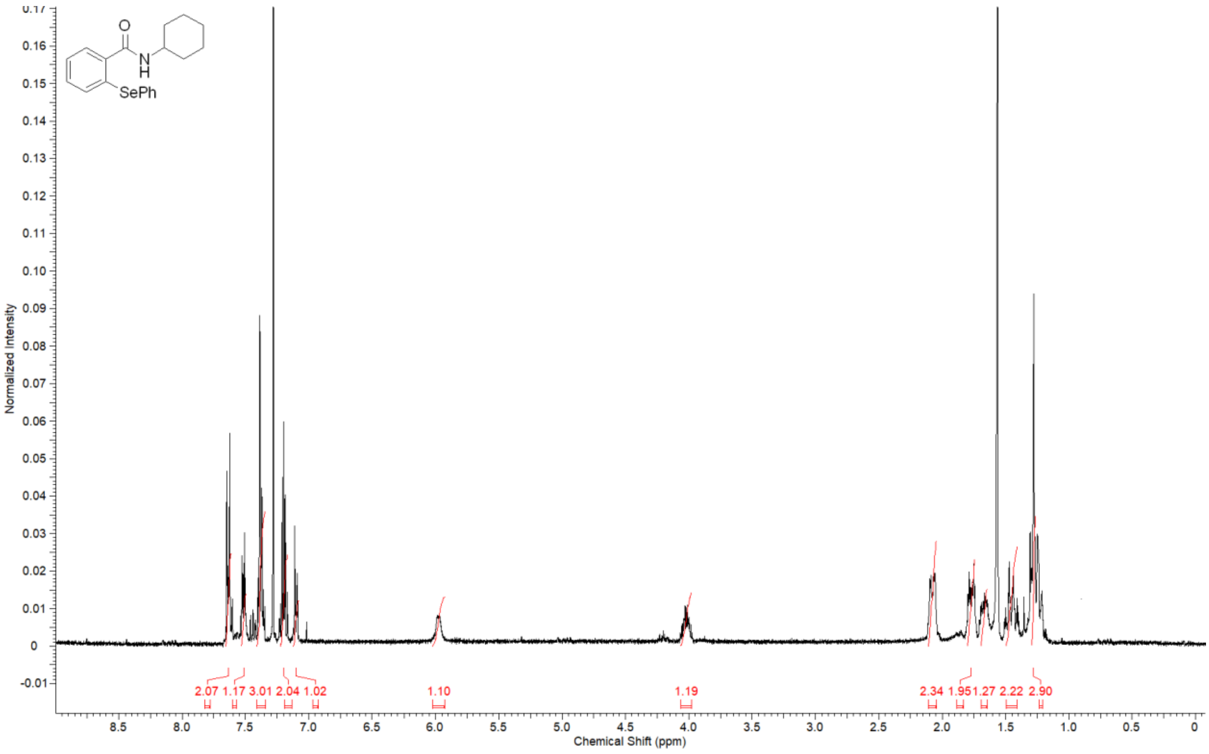

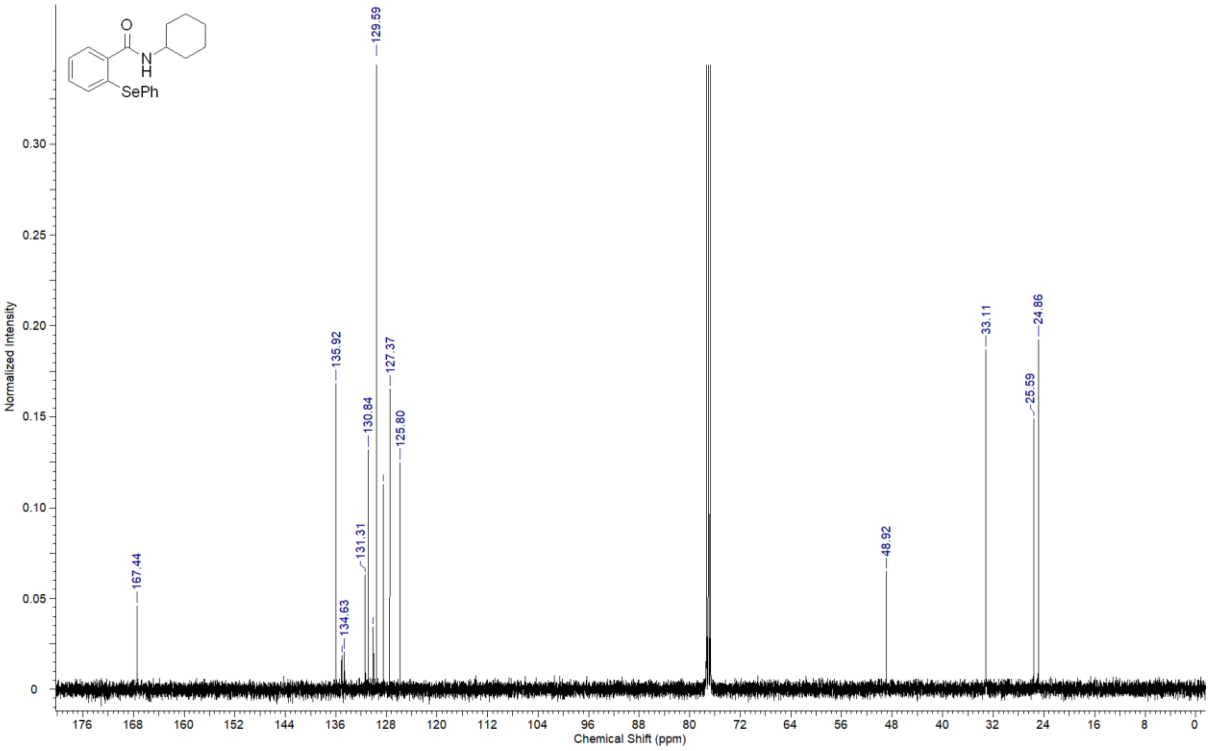
O
N
H
SePh
Normalized Intensity
0.30
0.25
0.20
0.15
0.10
0.05
0
167.44
135.92
134.63
131.31
130.84
129.59
127.37
125.80
48.92
33.11
25.59
24.86
176
168
160
152
144
136
128
120
112
104
96
88
80
72
64
56
48
40
32
24
16
8
0
Chemical Shift (ppm)

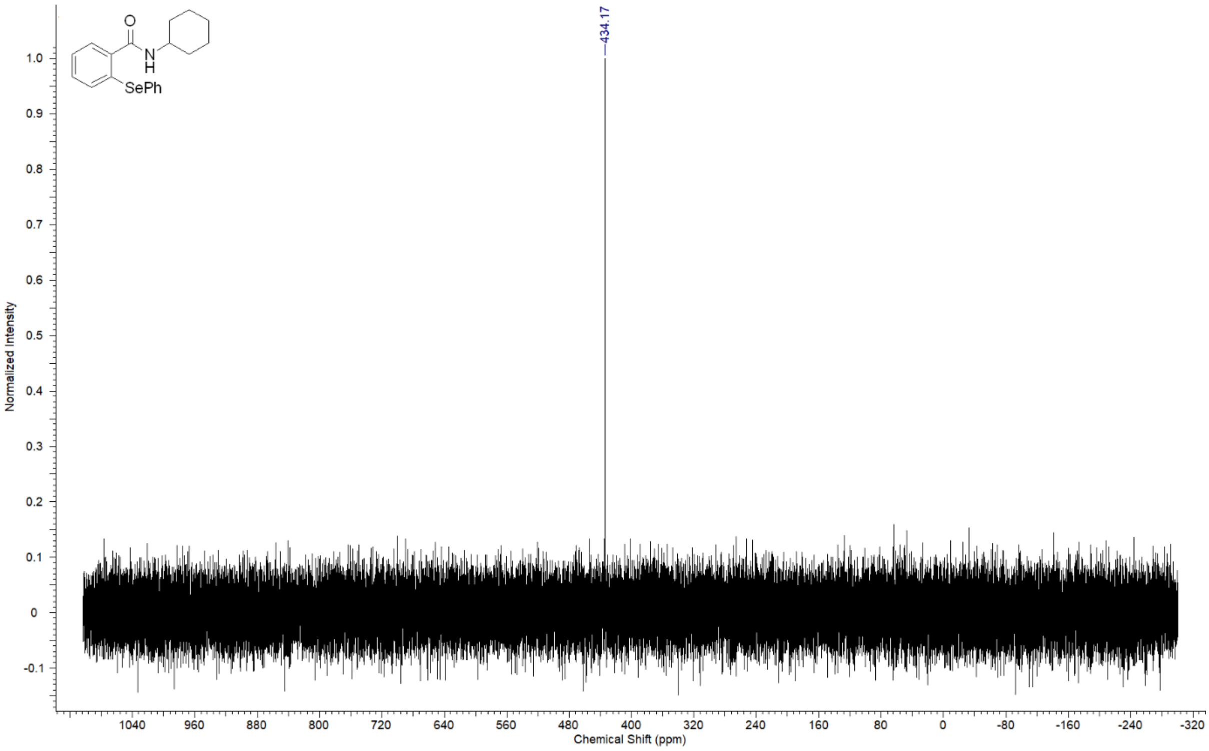
O
N
H
SePh
Normalized Intensity
1.0
0.9
0.8
0.7
0.6
0.5
0.4
0.3
0.2
0.1
0
-0.1
434.17
1040
960
880
800
720
640
560
480
400
320
240
160
80
0
-80
-160
-240
-320
Chemical Shift (ppm)

N-phenyl-2-(phenylselanyl)benzamide **13b**

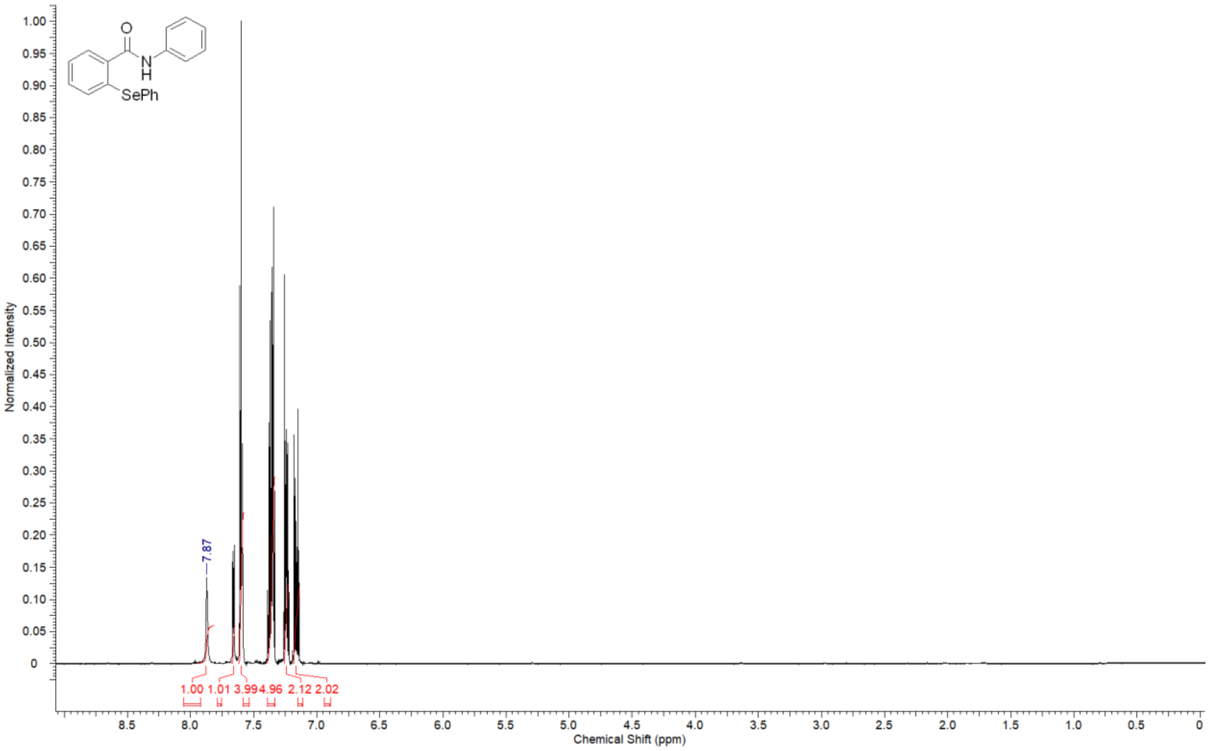

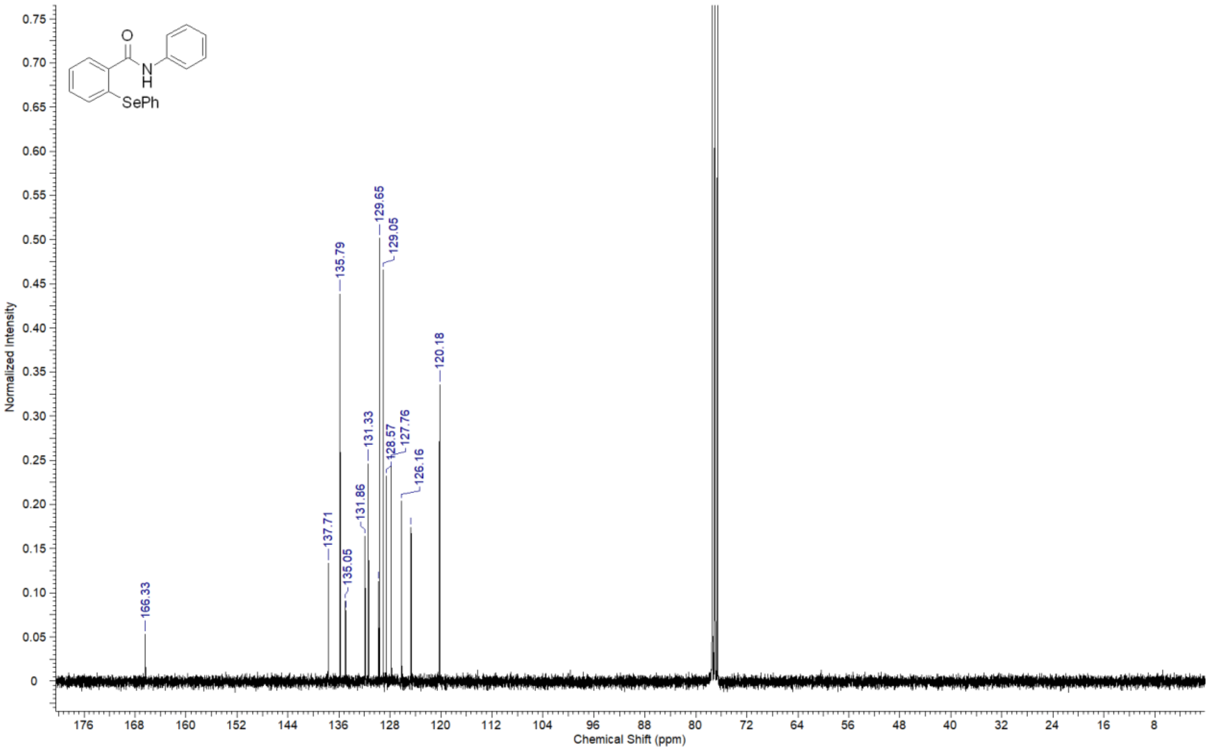

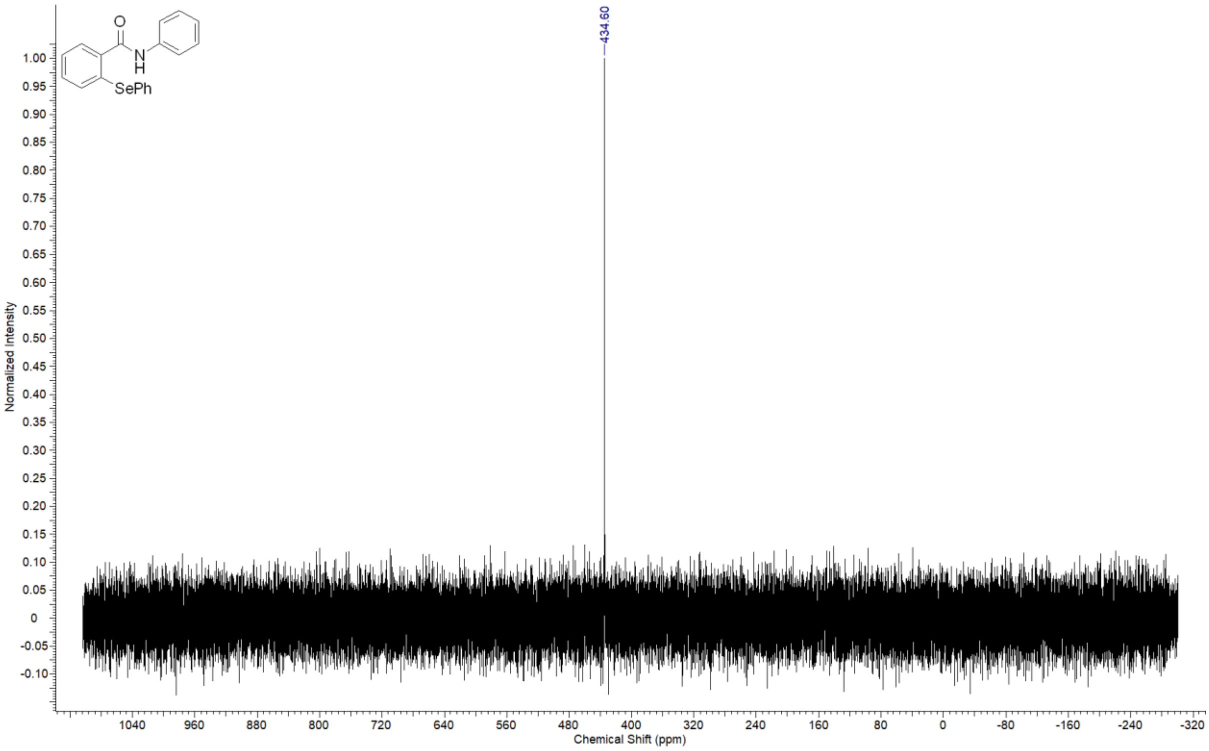

N-(*p*-chlorophenyl)-2-(phenylselanyl)benzamide **14b**

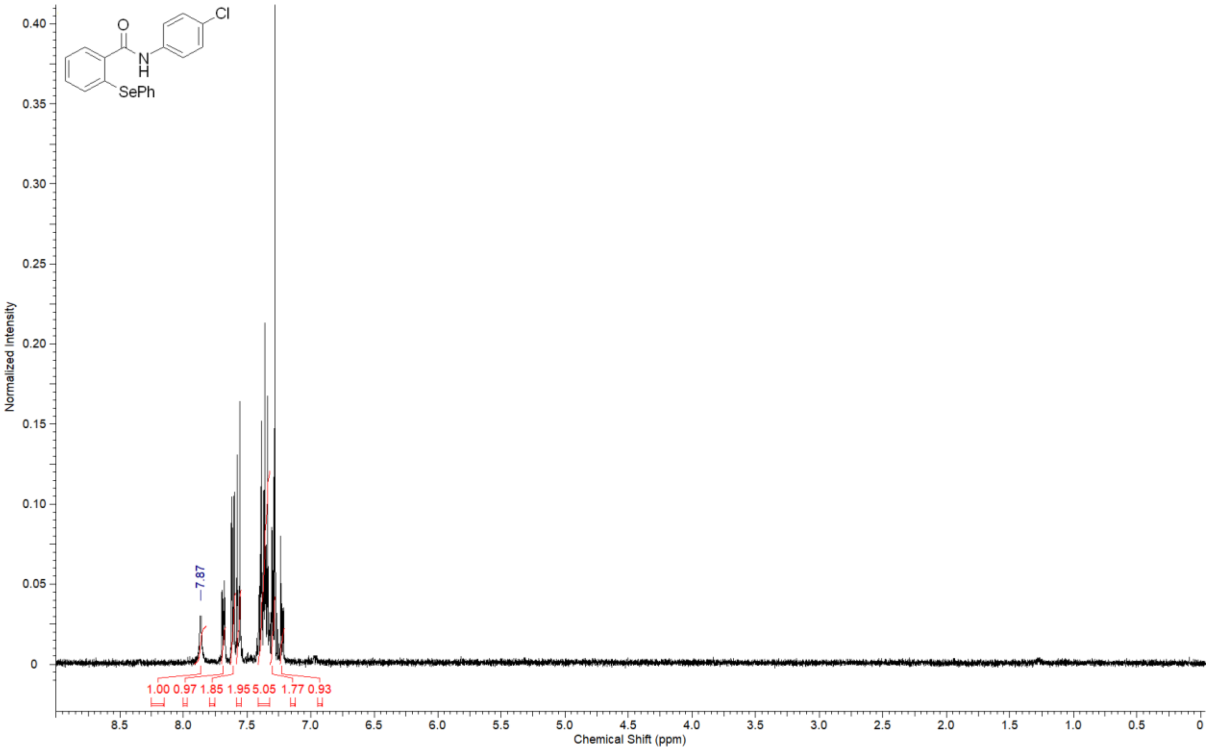

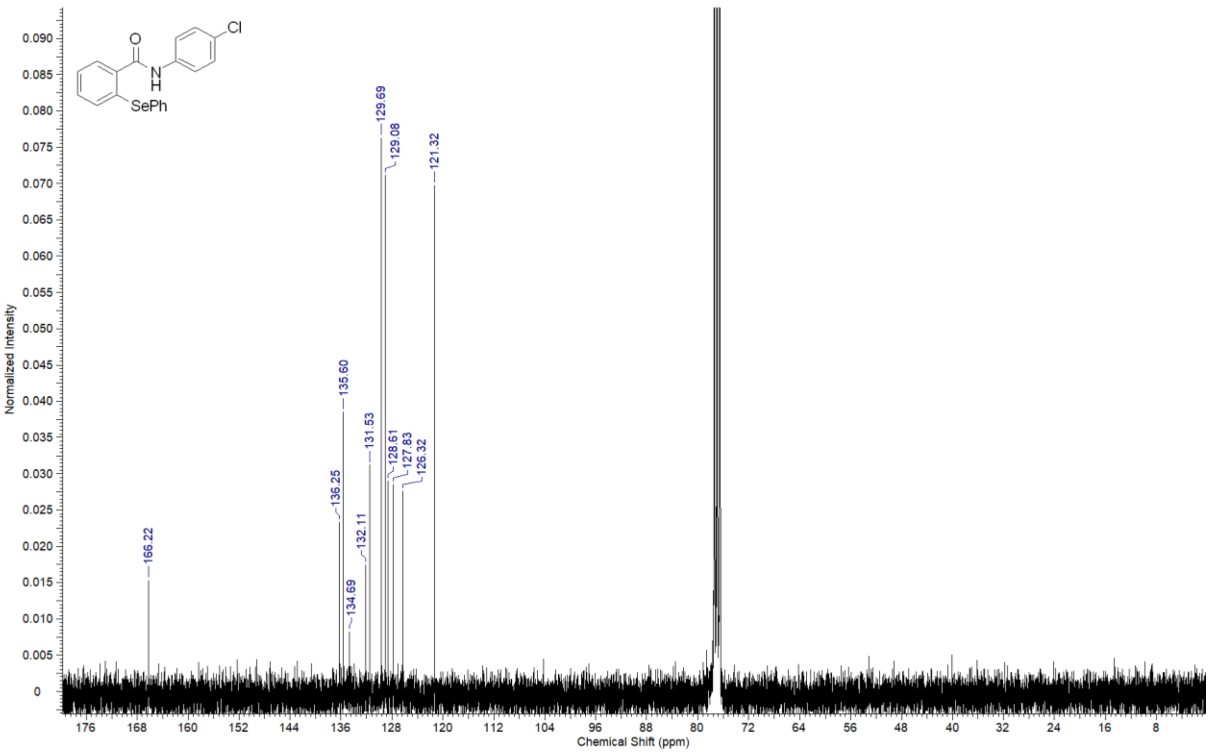
O
N
H
Cl
SePh
166.22
136.25
135.60
134.69
132.11
131.53
129.69
129.08
128.61
127.83
126.32
121.32
Normalized Intensity
Chemical Shift (ppm)

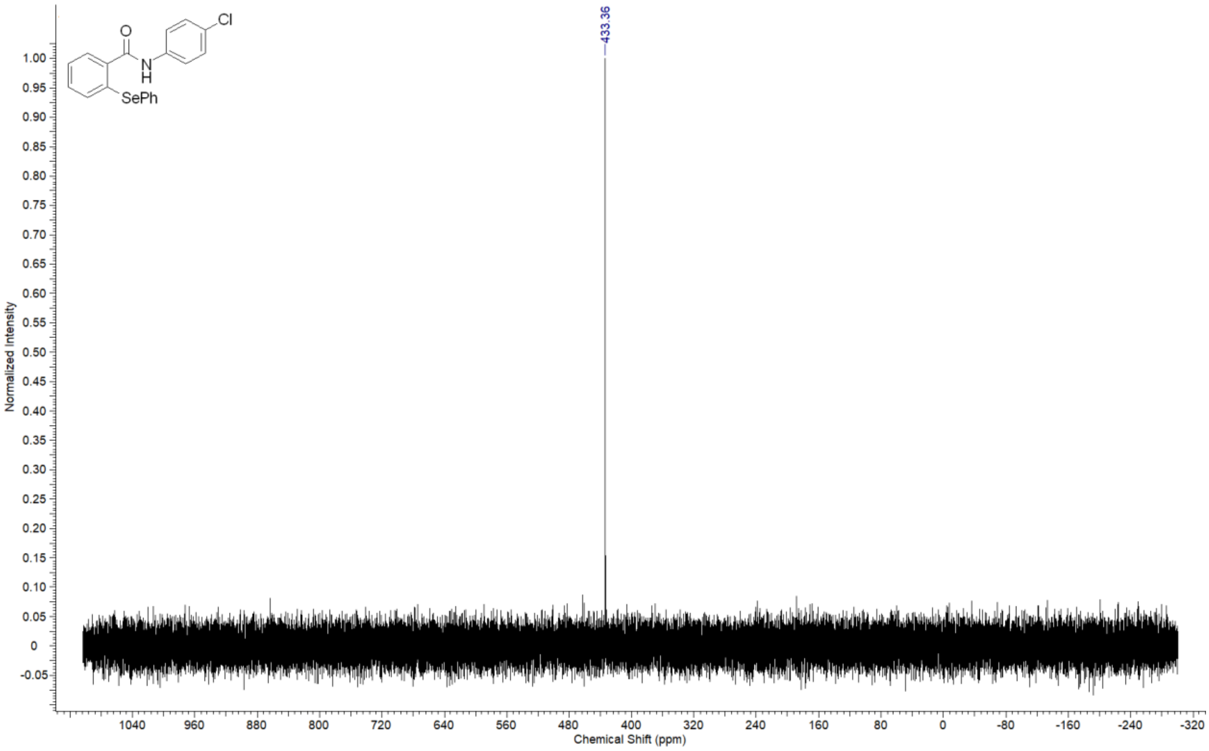
O
N
H
Cl
SePh
433.36
Normalized Intensity
Chemical Shift (ppm)

N-(*p*-bromophenyl)-2-(phenylselanyl)benzamide **15b**

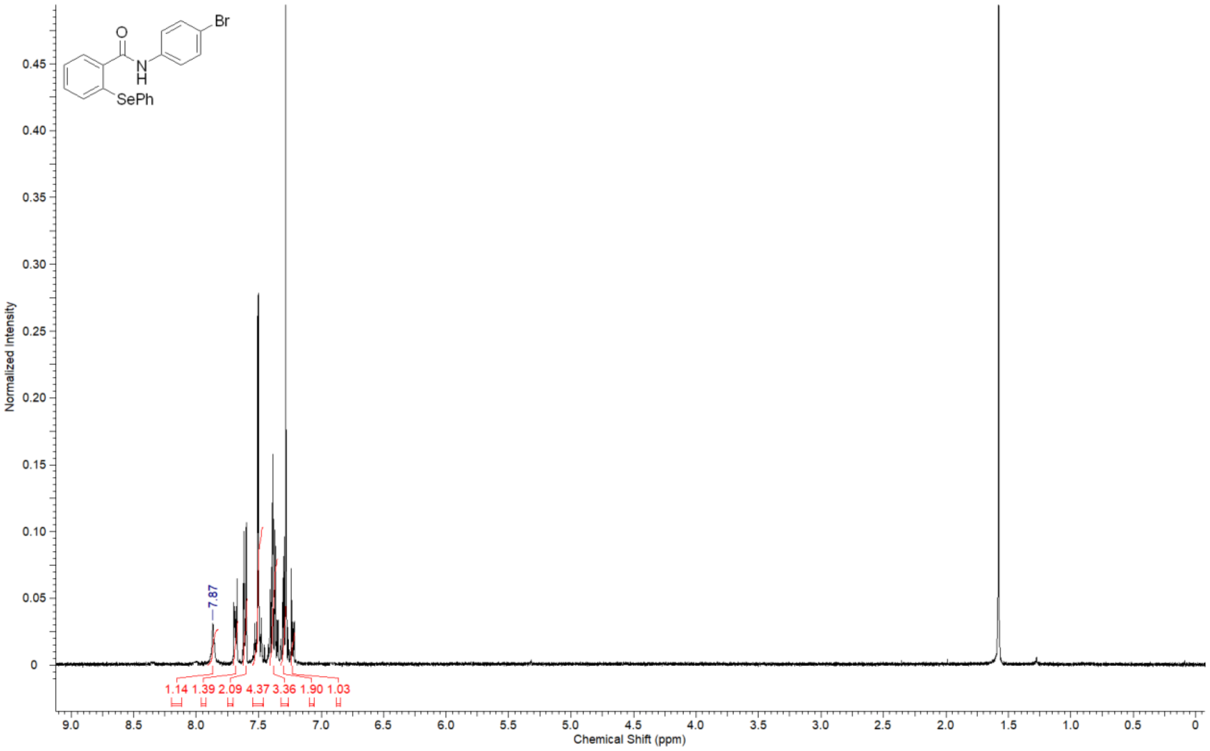

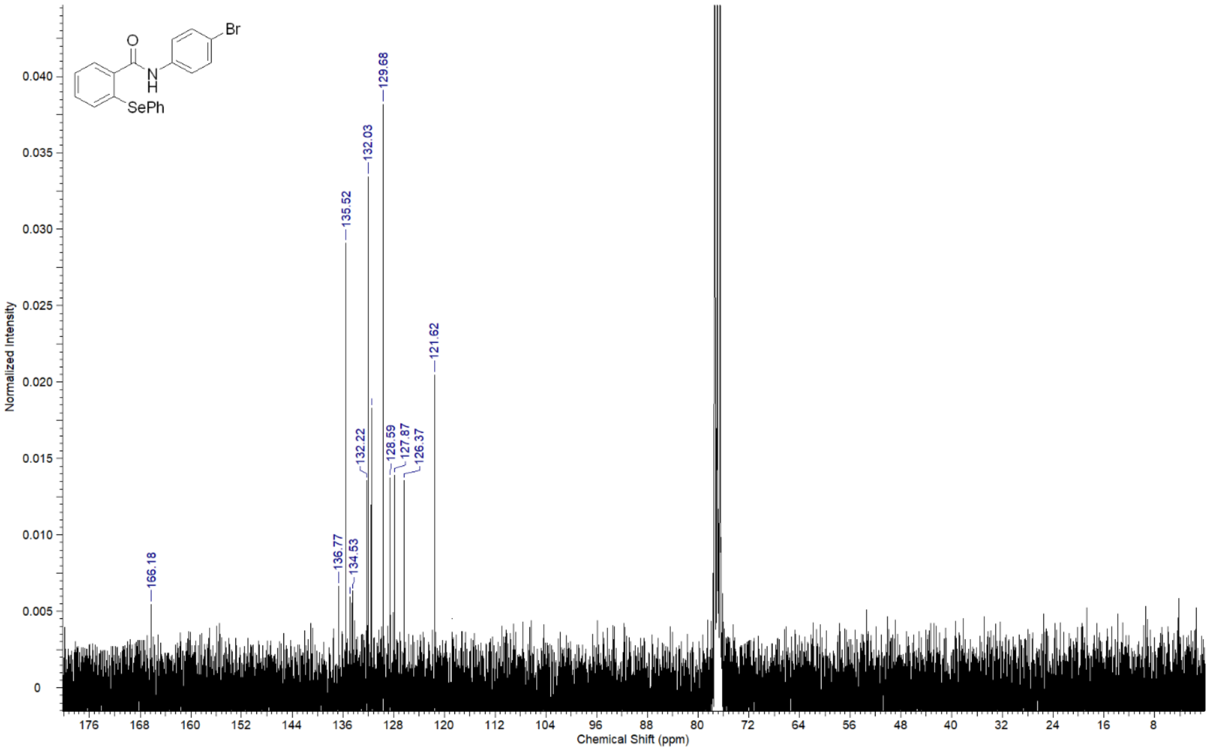

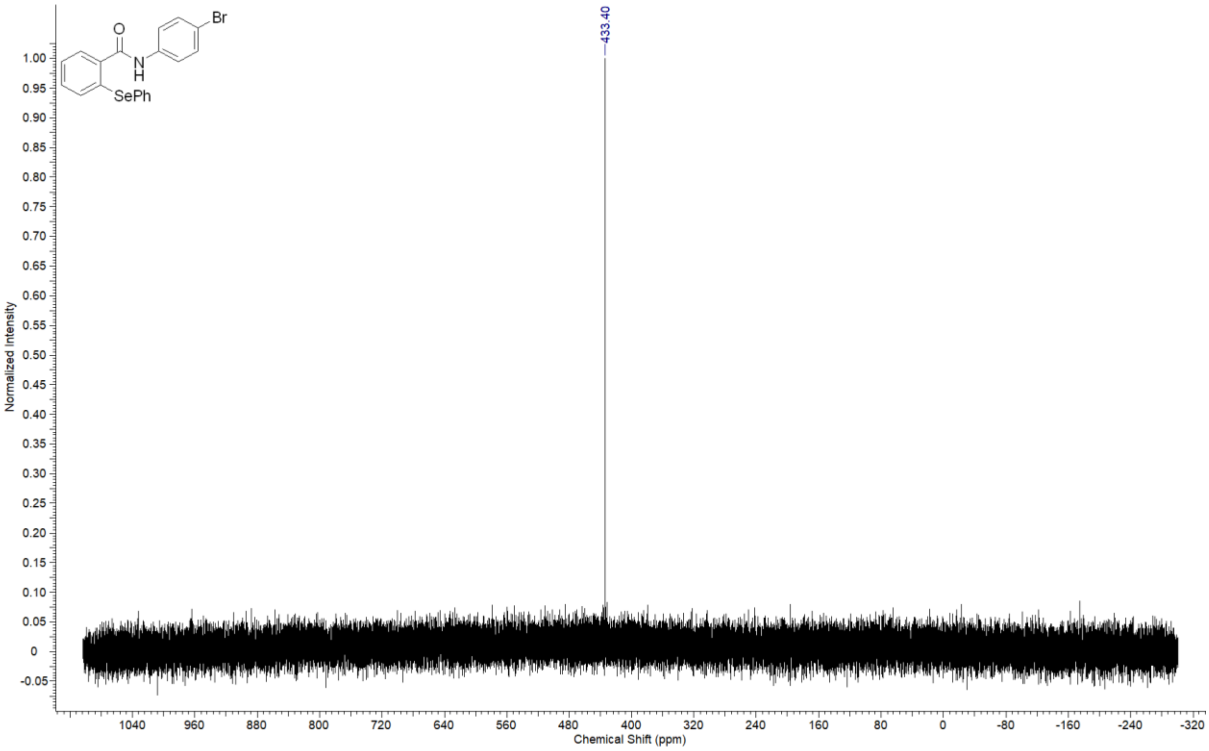

N-(*p*-iodophenyl)-2-(phenylselanyl)benzamide **16b**

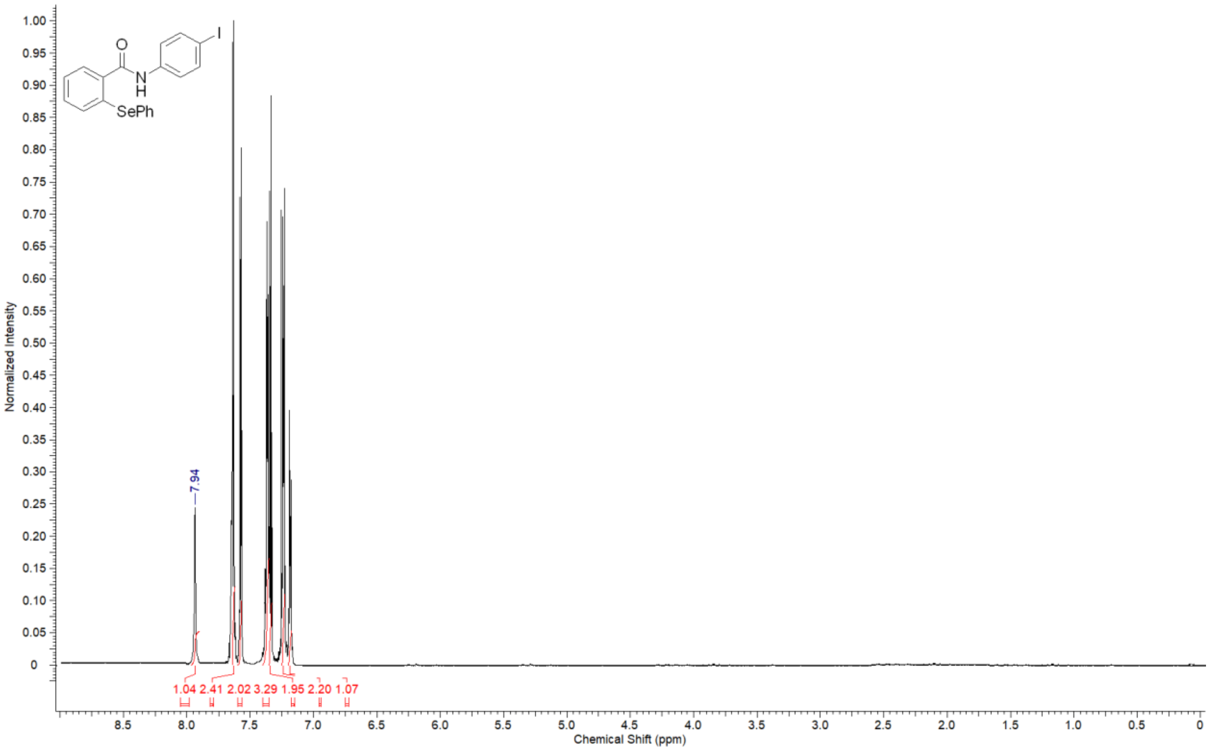

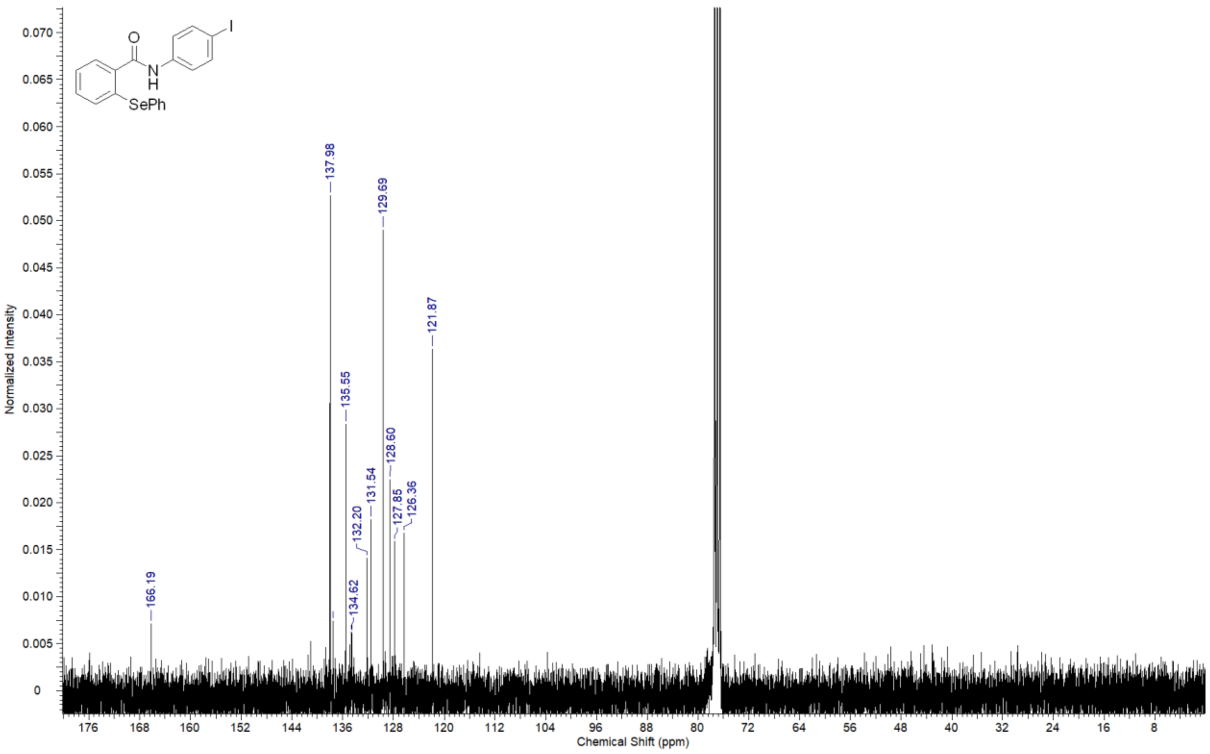
O
N
H
I
SePh
137.98
129.69
121.87
135.55
128.60
131.54
132.20
127.85
126.36
134.62
166.19
Normalized Intensity
Chemical Shift (ppm)

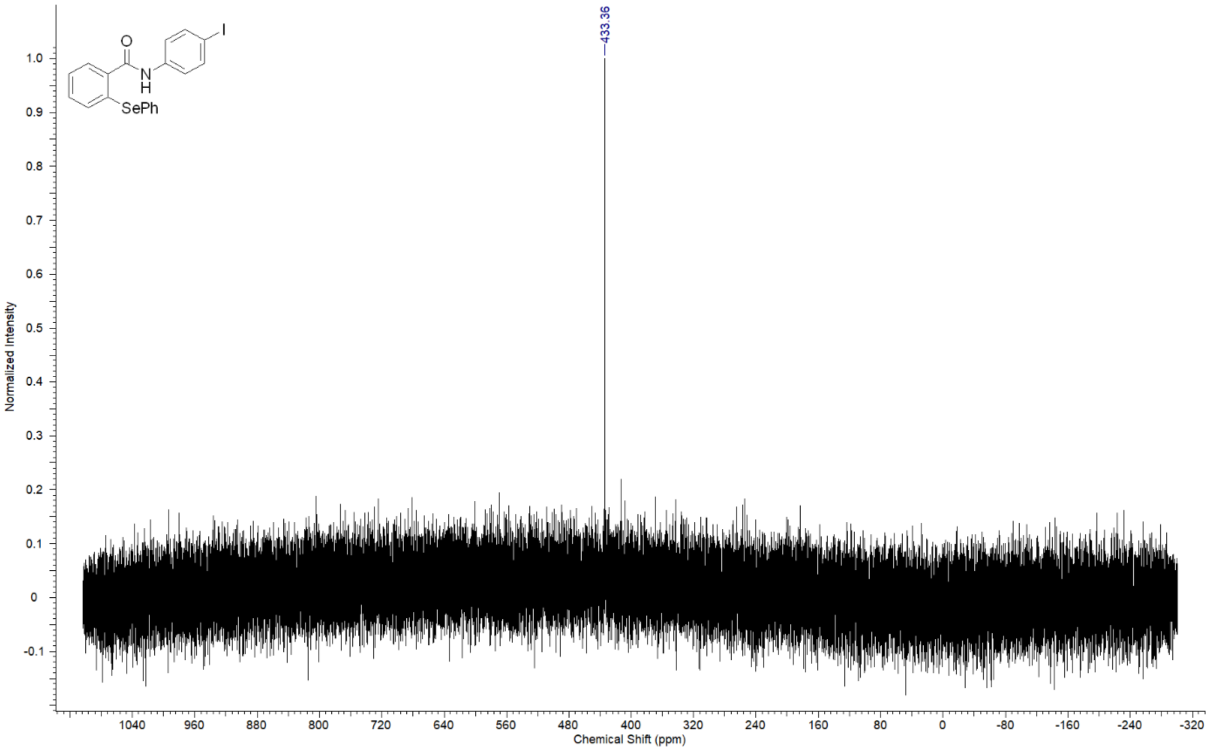
O
N
H
I
SePh
433.36
Normalized Intensity
Chemical Shift (ppm)

N-(*p*-metoxyphenyl)-2-(phenylselanyl)benzamide **17b**

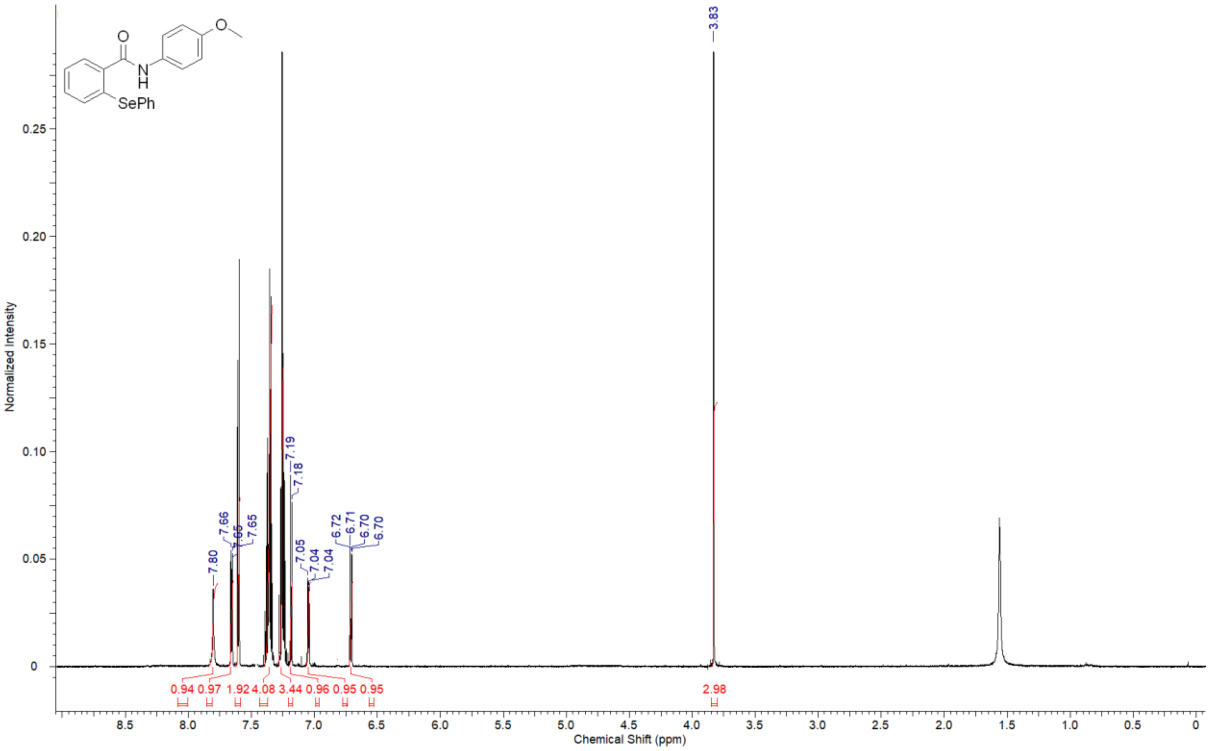

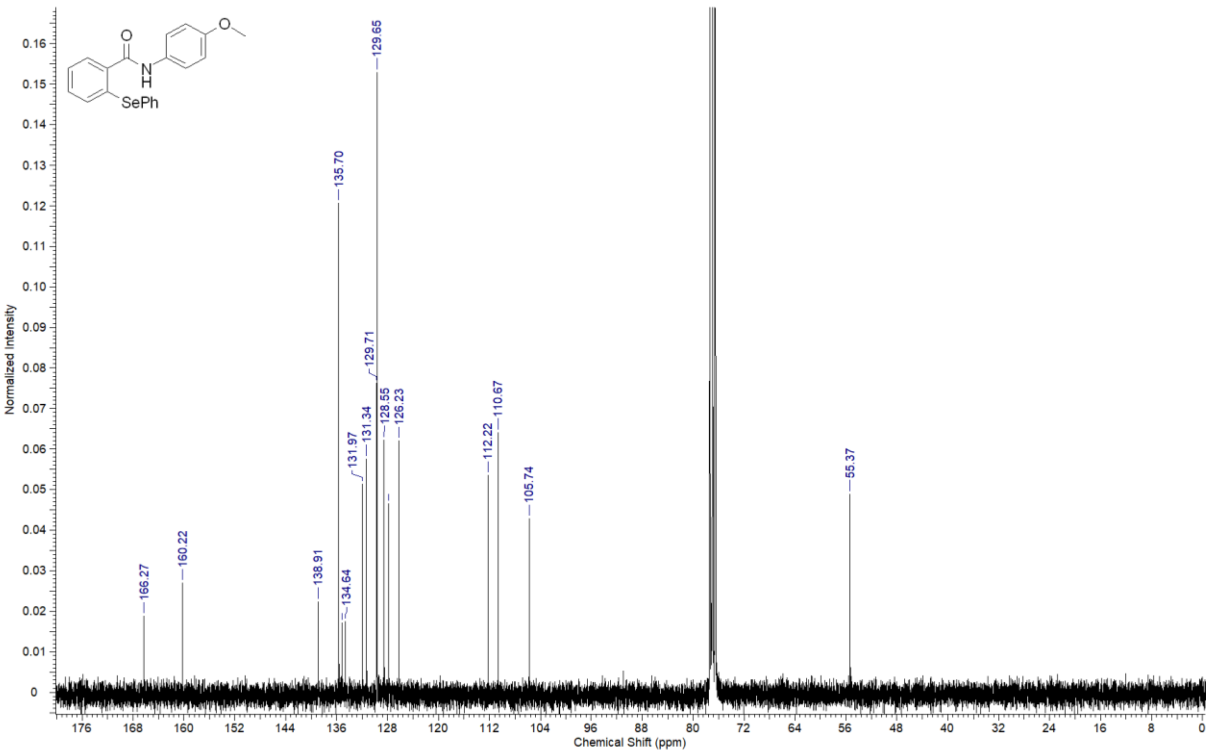

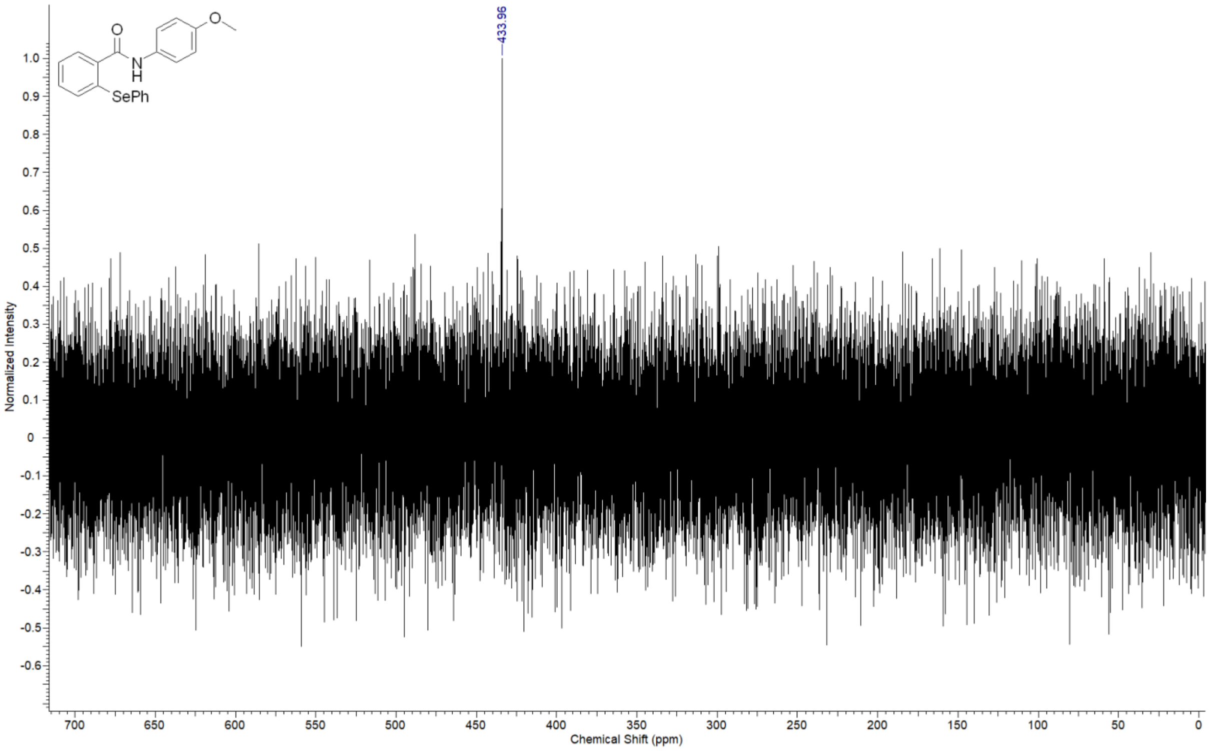

(−)-*N*-(1*R*,2*S*,5*R*)-menthyl-2-(phenylselanyl)benzamide **18b**

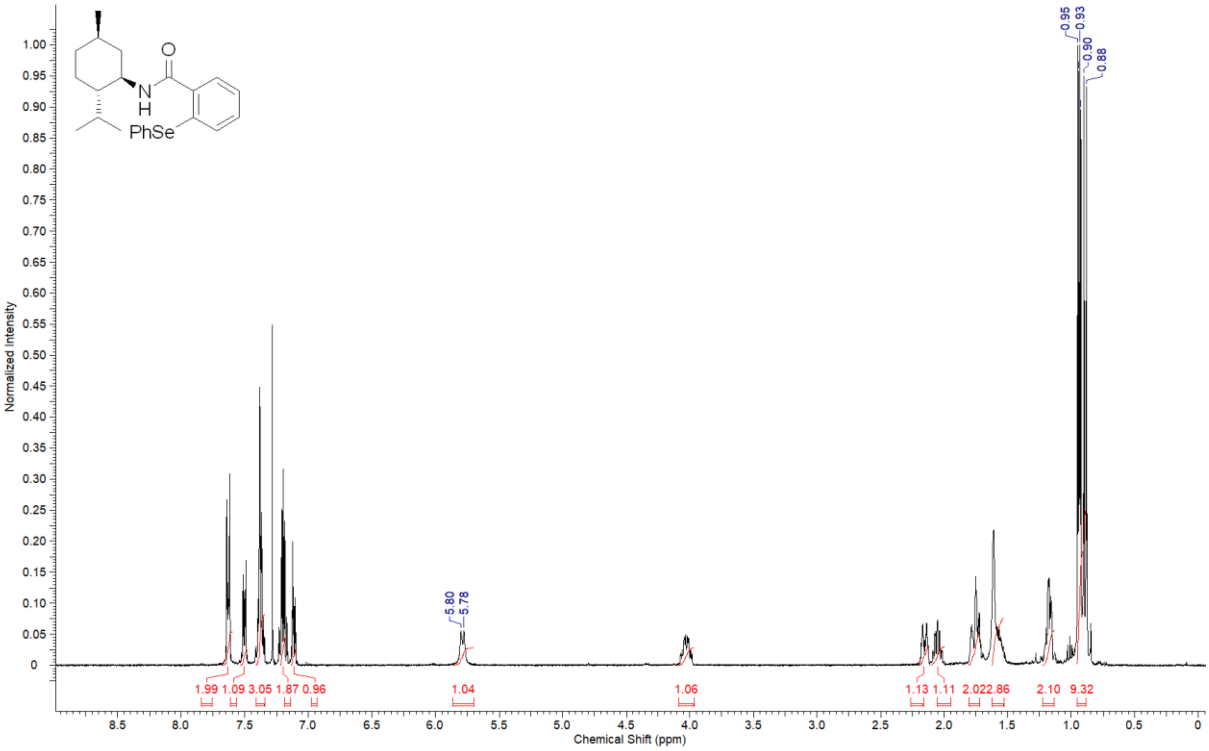

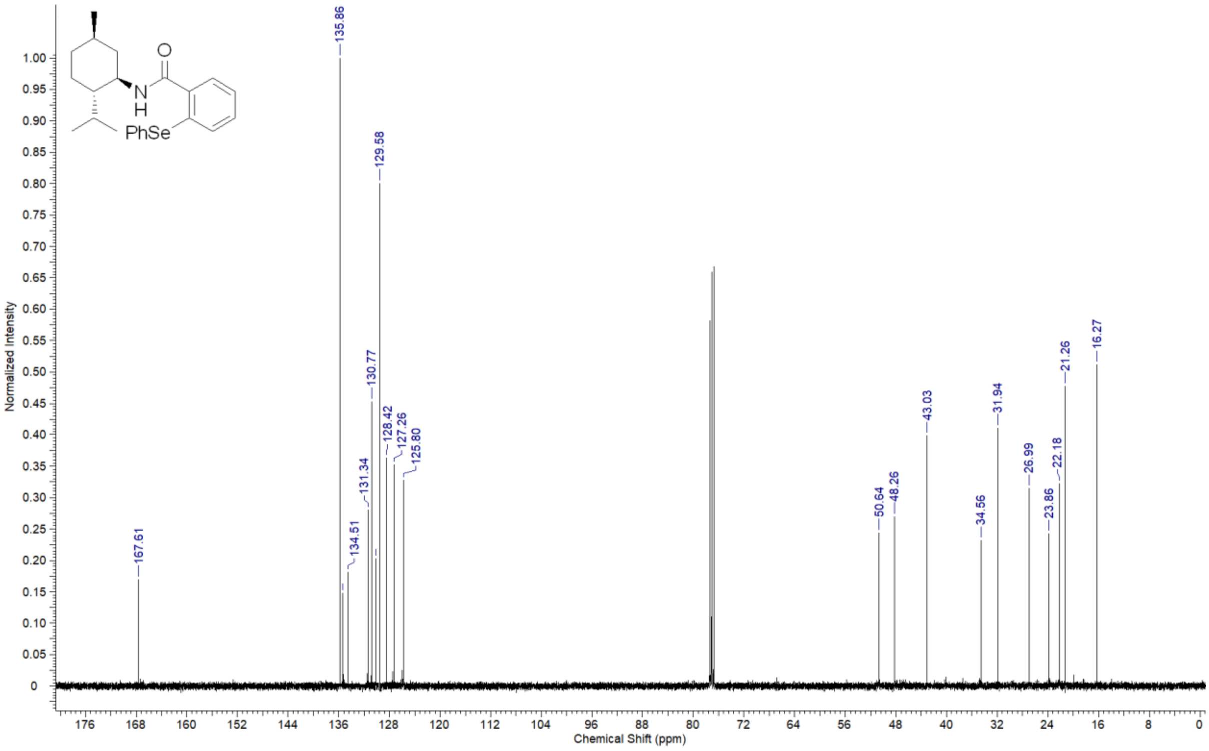

O
N
H
PhSe
Normalized Intensity
Chemical Shift (ppm)
167.61
135.86
134.51
131.34
130.77
129.58
128.42
127.26
125.80
50.64
48.26
43.03
34.56
31.94
26.99
23.86
22.18
21.26
16.27

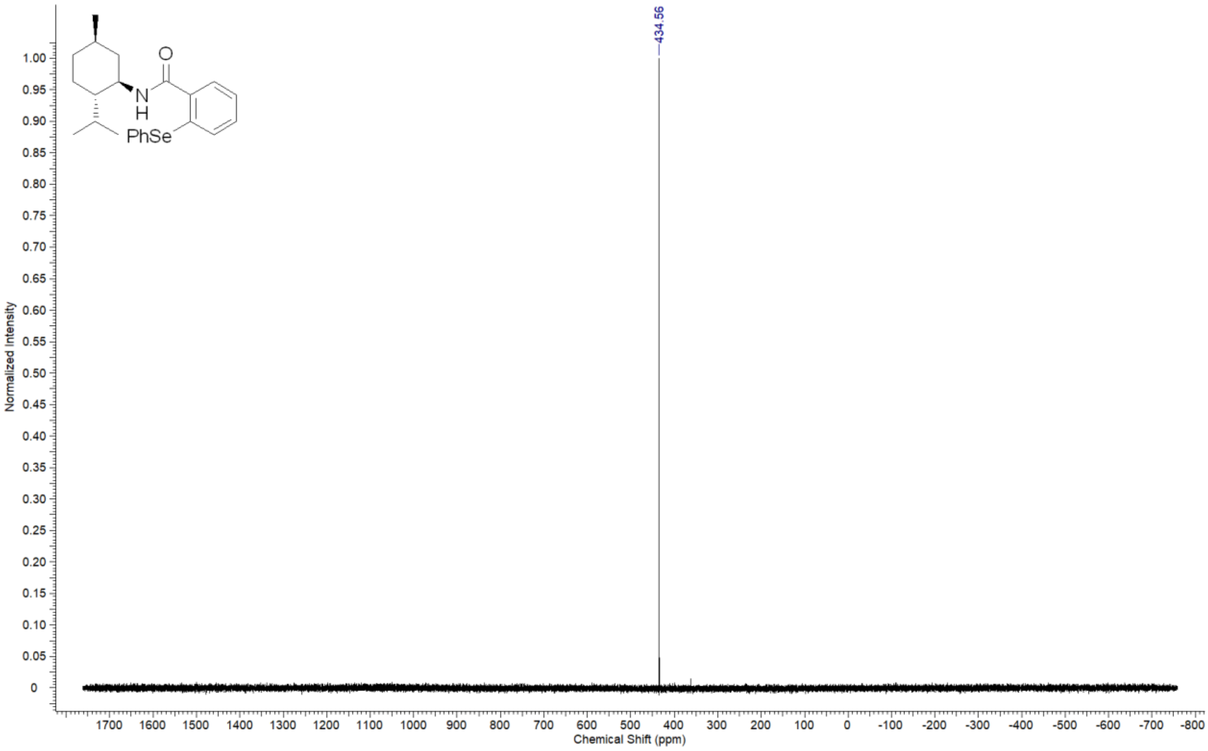

O
N
H
PhSe
Normalized Intensity
Chemical Shift (ppm)
434.56

(-)-*N*-(1*S*,2*R*,3*S*,6*R*)-(2-caranyl)-2-(phenylselanyl)benzamide **19b**

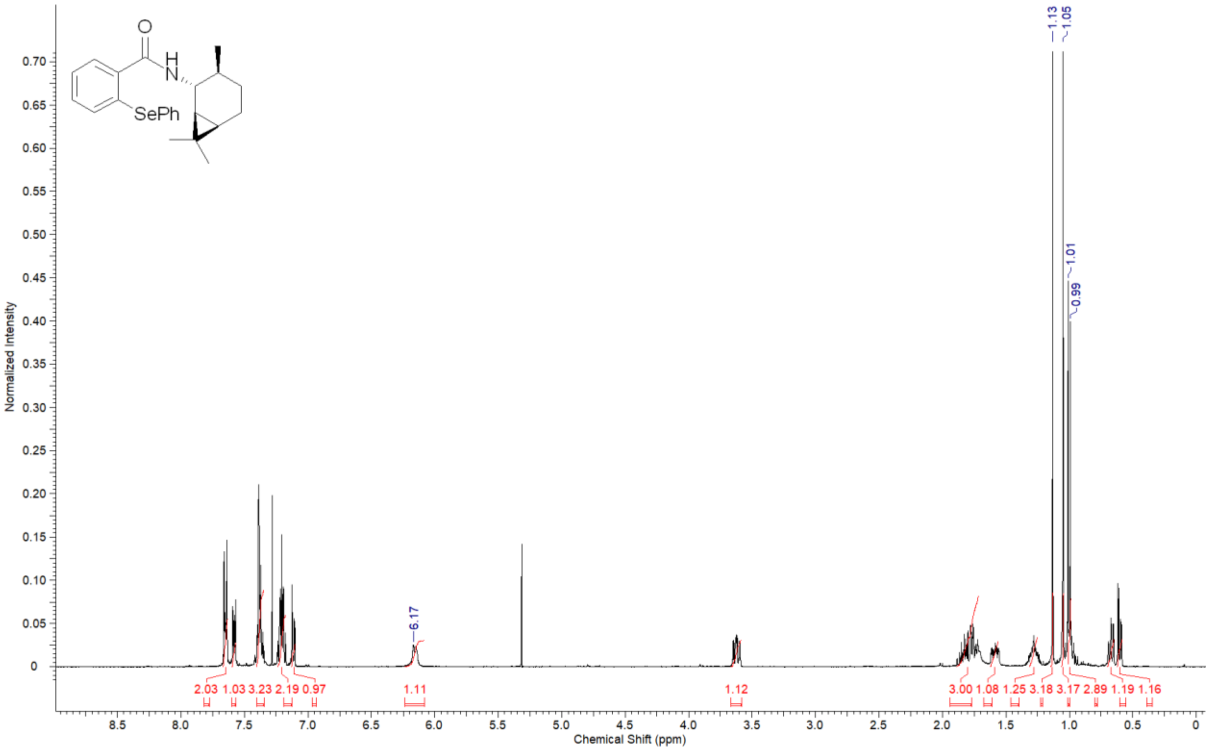

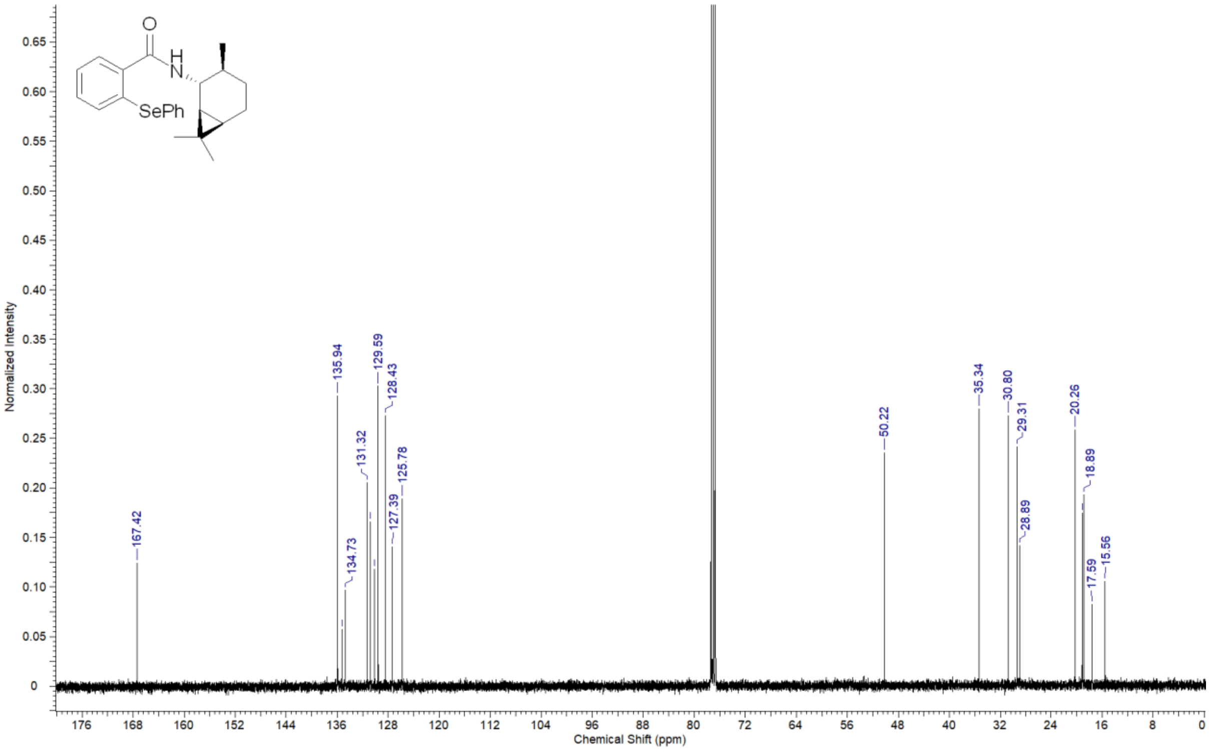

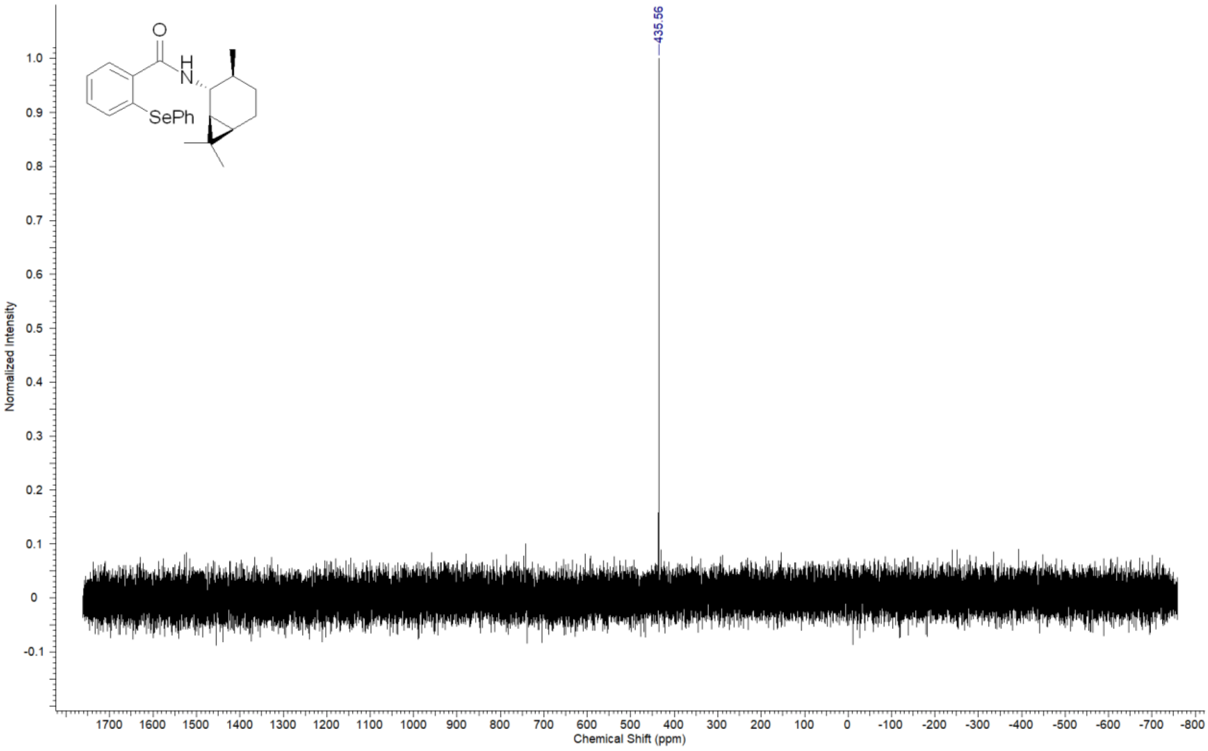

N-borynylo-2-(phenylselanyl)benzamide **20b**

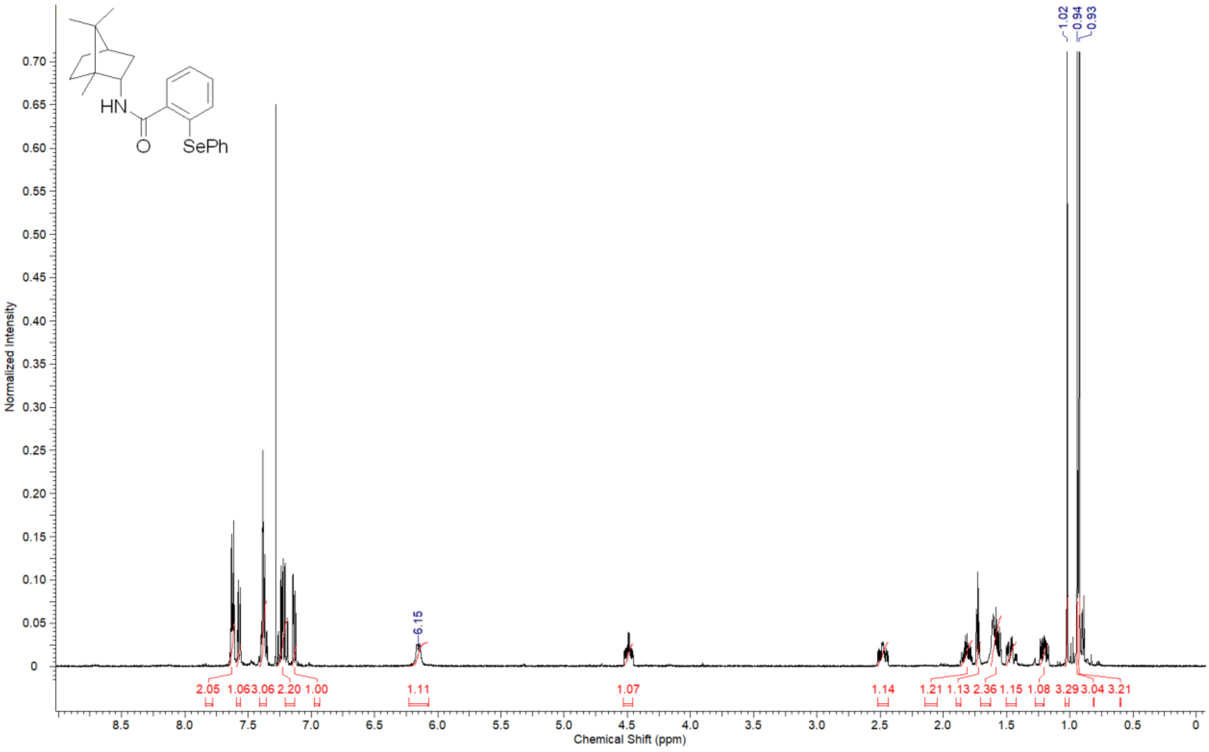

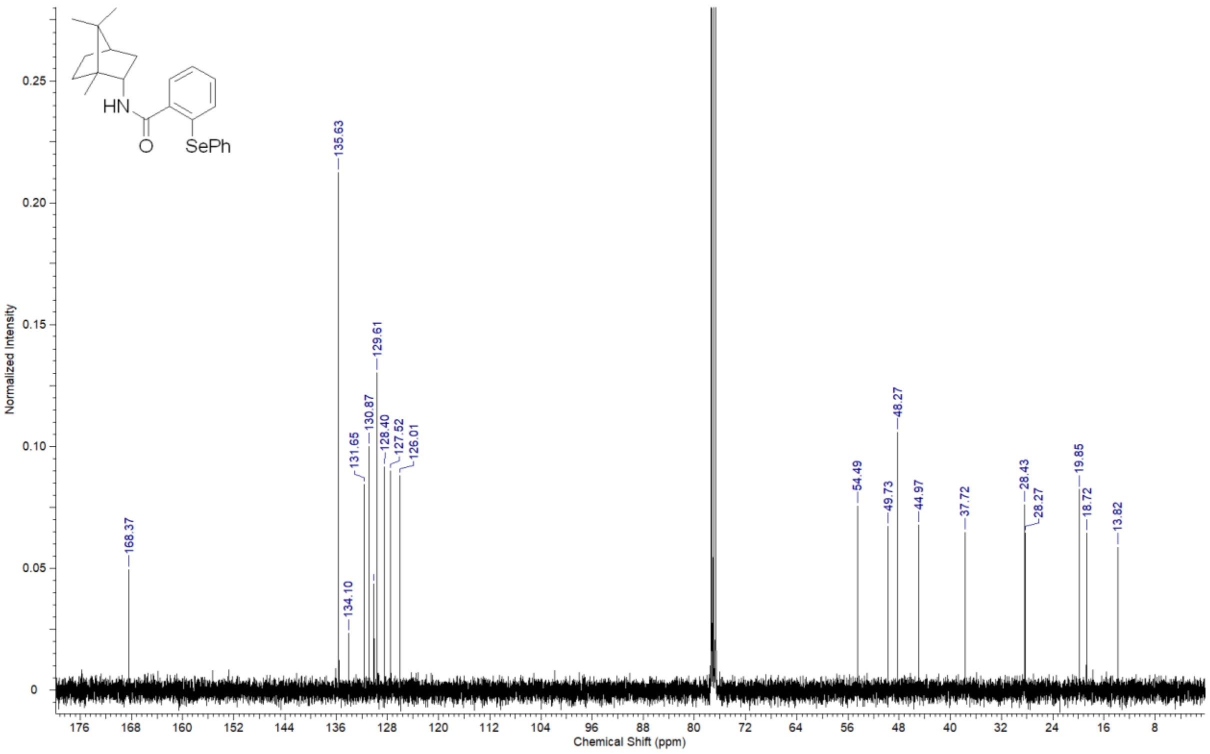
HN
O
SePh
135.63
134.10
131.65
130.87
129.61
128.40
127.52
126.01
168.37
54.49
49.73
48.27
44.97
37.72
28.43
28.27
19.85
18.72
13.82
Normalized Intensity
Chemical Shift (ppm)

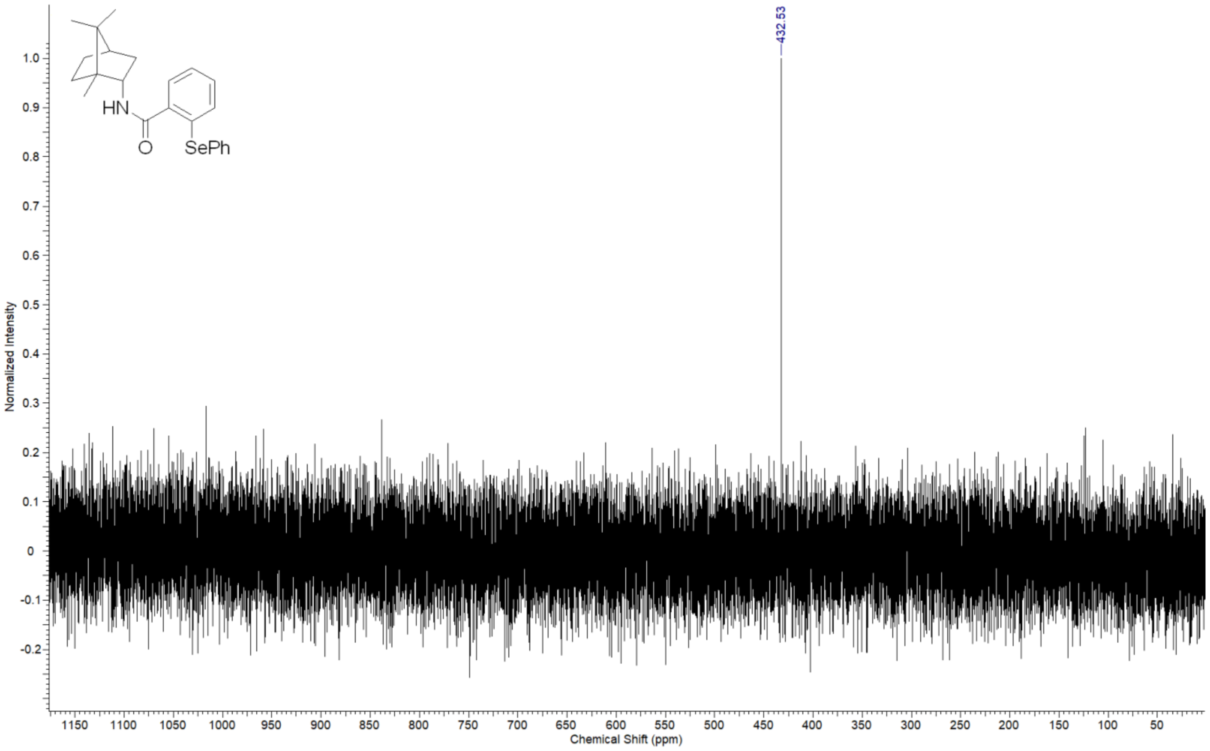
HN
O
SePh
432.53
Normalized Intensity
Chemical Shift (ppm)

(-)-*N*-(1*S*,2*R*,5*S*)-myrtanyl-2-(phenylselanyl)benzamide **21b**

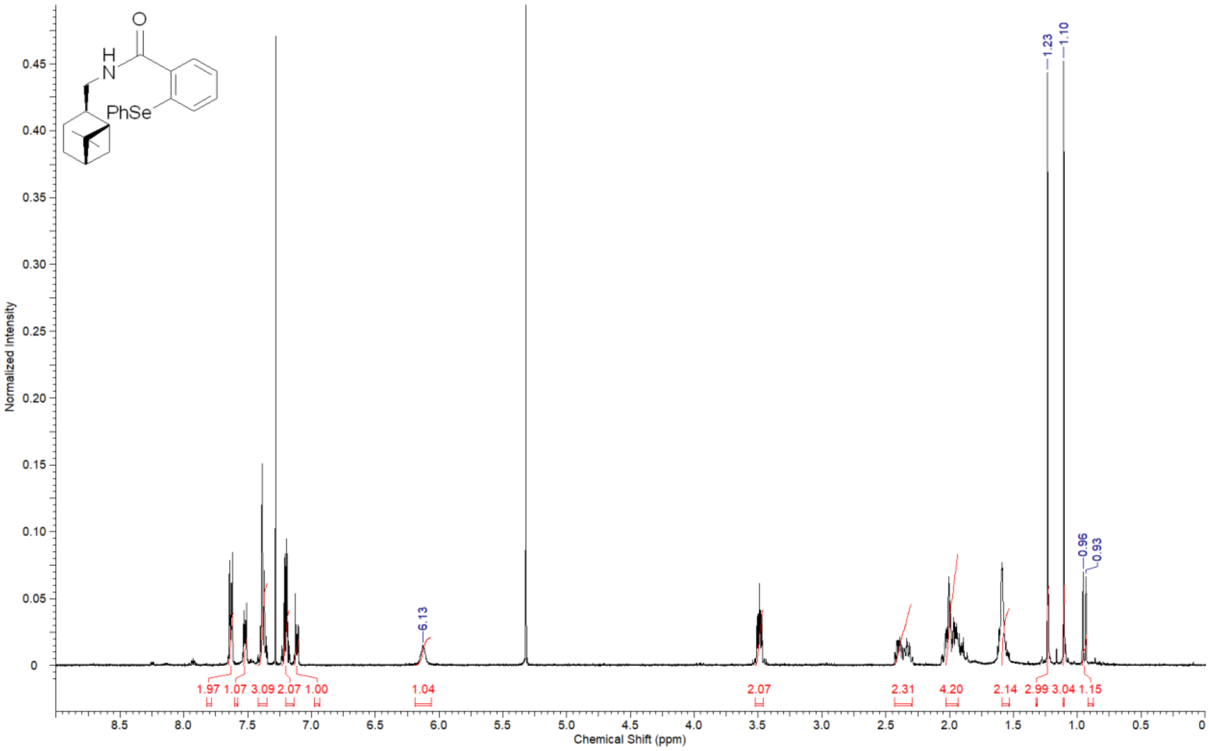

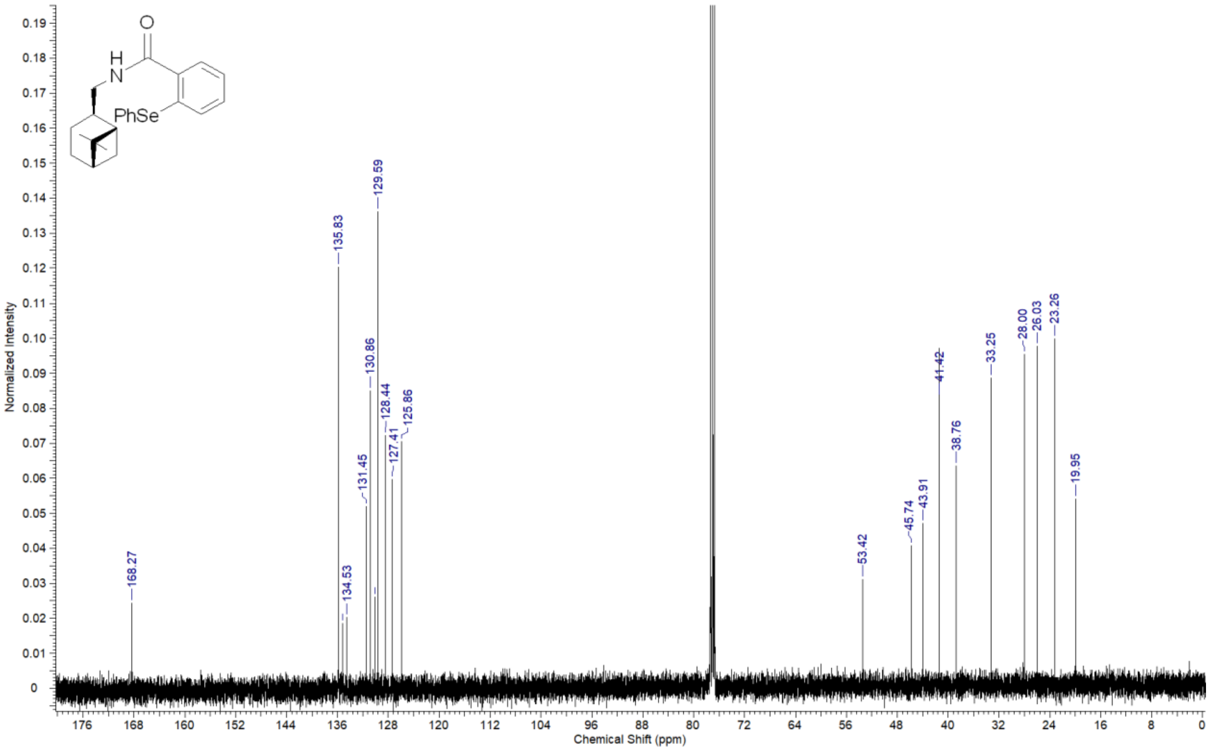

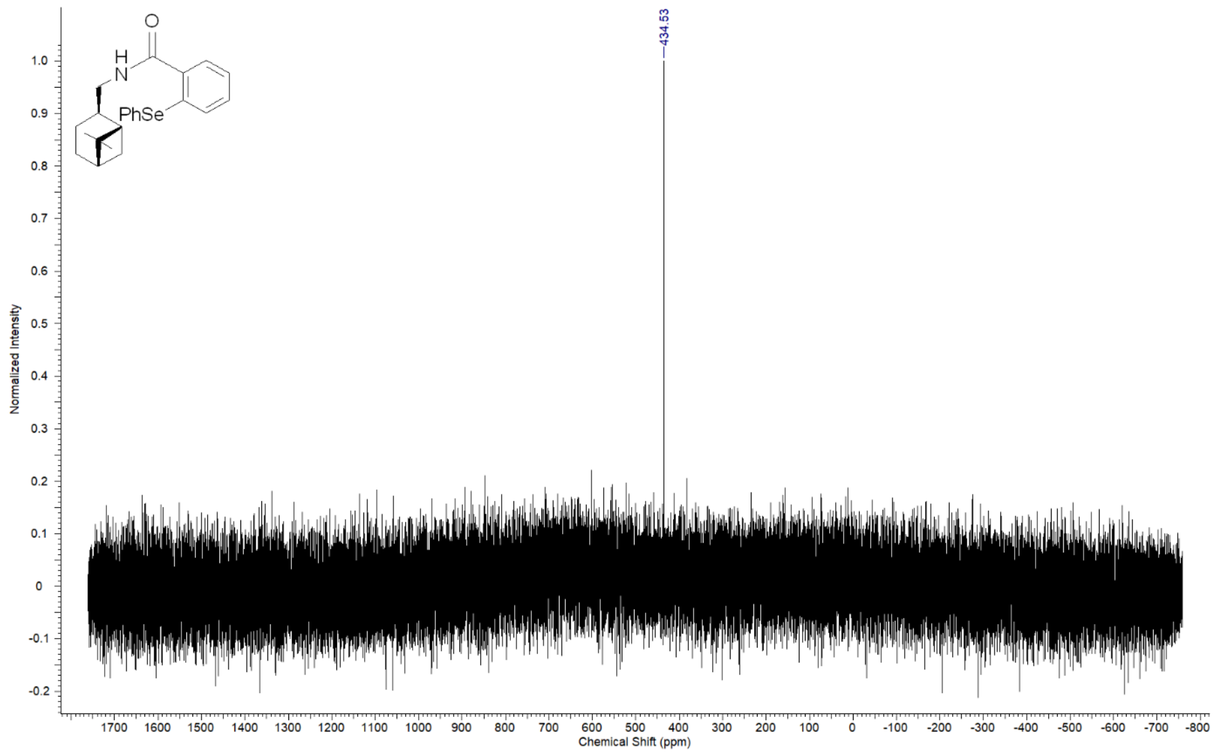

(-)-*N*-(1*R*,2*R*,3*R*,5*S*)-isopinocamphyl-2-(phenylselanyl)benzamide **22b**

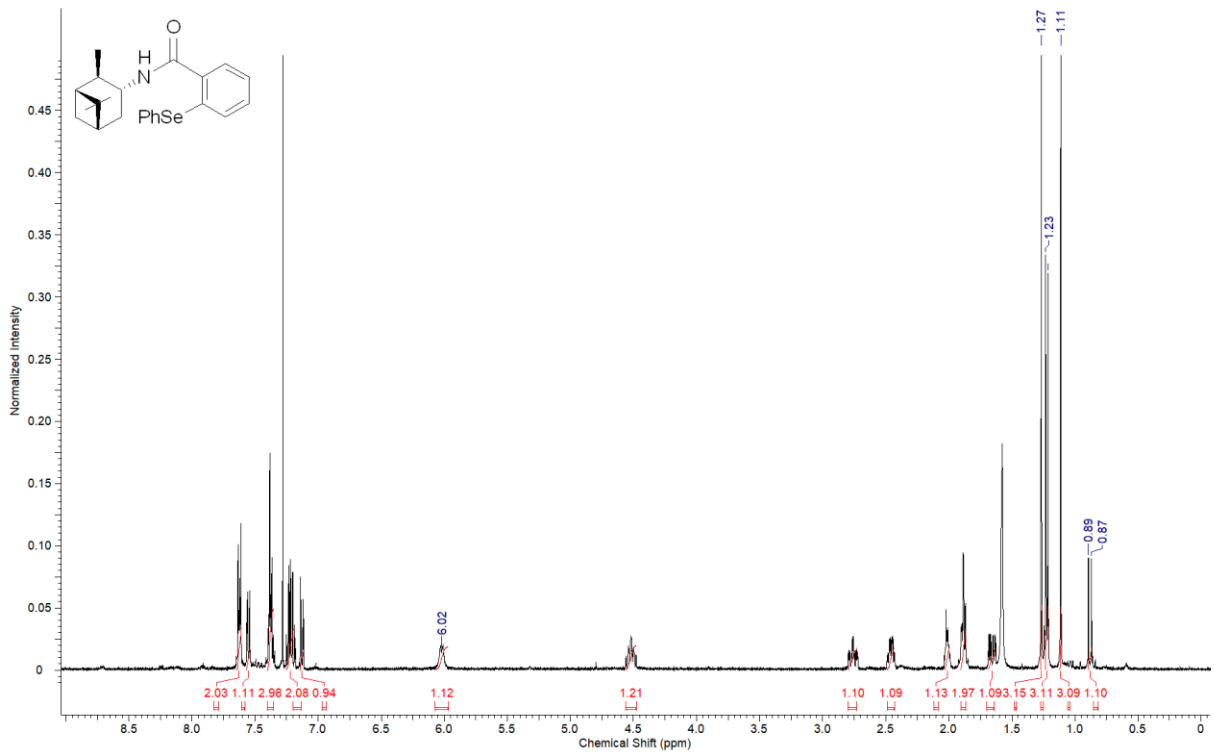

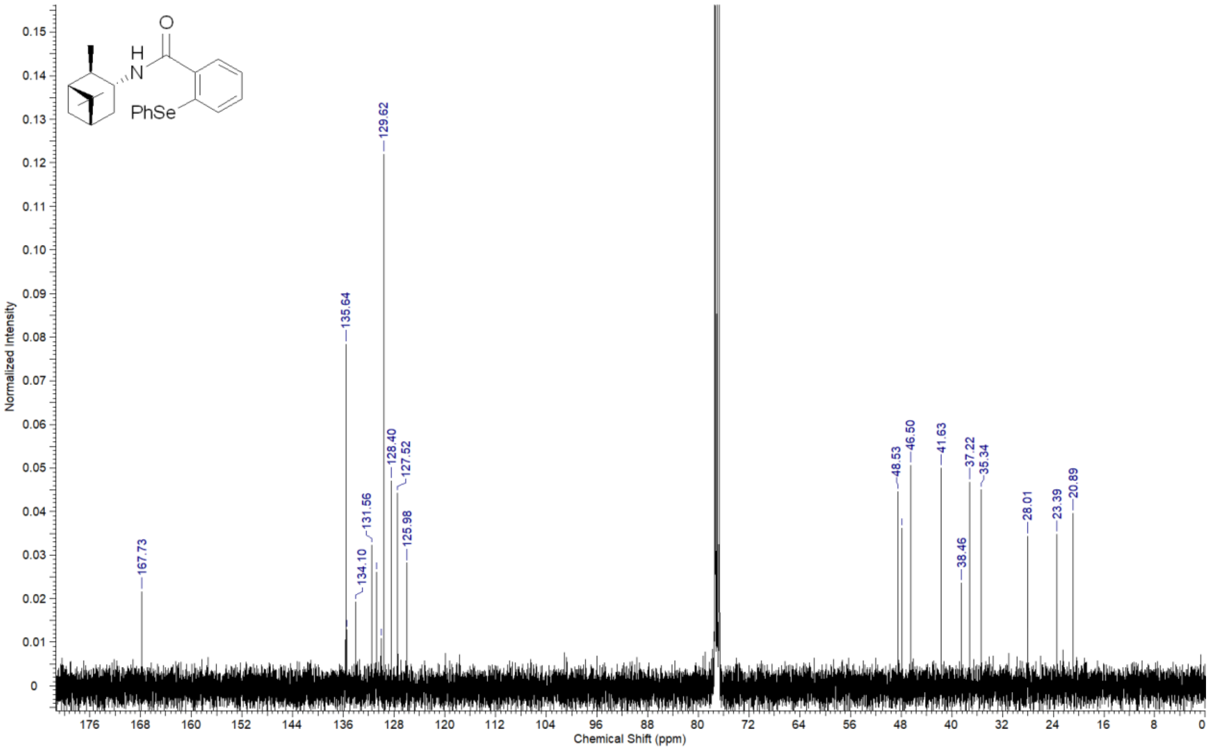

H
N
O
PhSe
167.73
135.64
134.10
131.56
129.62
128.40
127.52
125.98
48.53
46.50
41.63
38.46
37.22
35.34
28.01
23.39
20.89
Normalized Intensity
Chemical Shift (ppm)

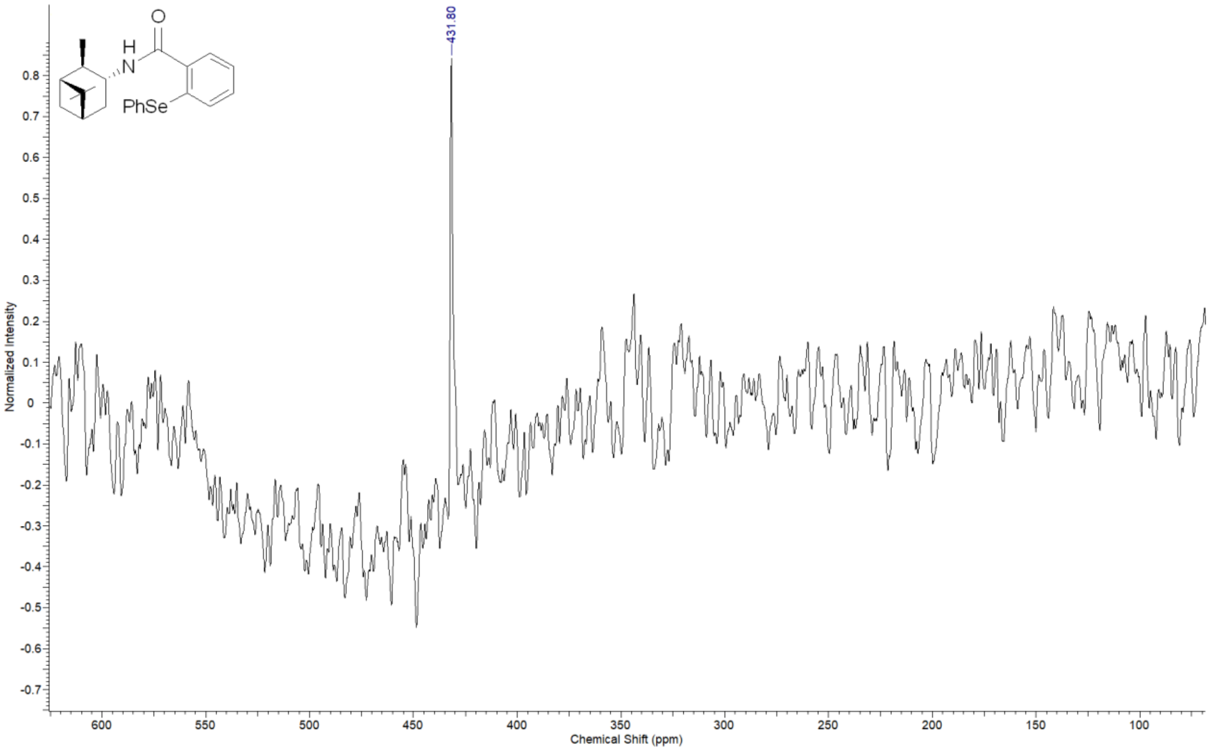

H
N
O
PhSe
431.80
Normalized Intensity
Chemical Shift (ppm)

(+)-*N*-(1*R*,2*R*,3*R*,5*S*)-isopinocamphyl-2-(phenylselanyl)benzamide **23b**

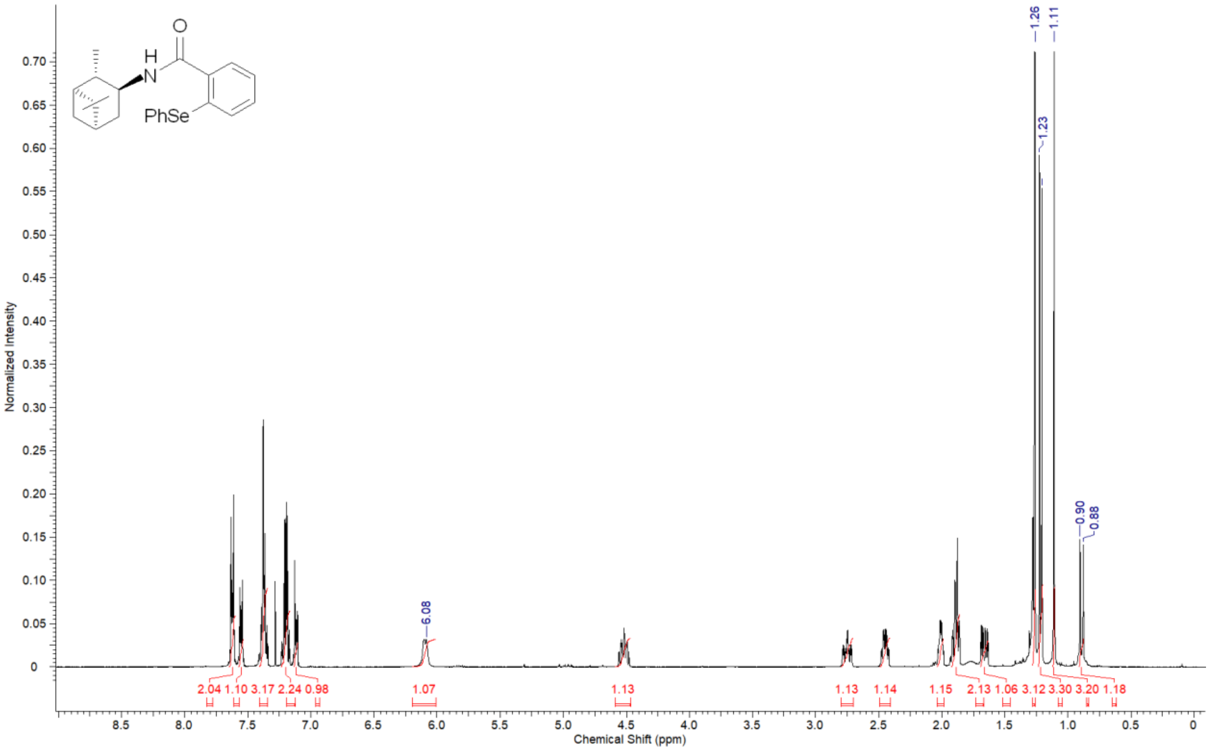

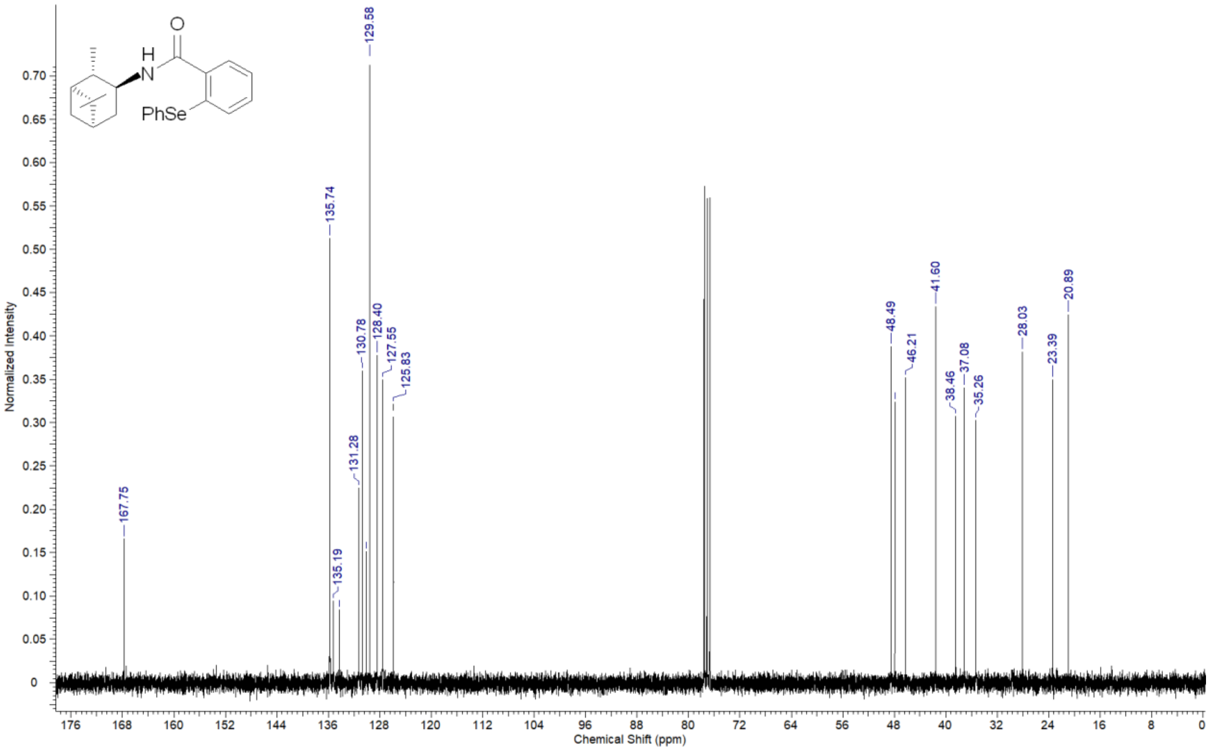

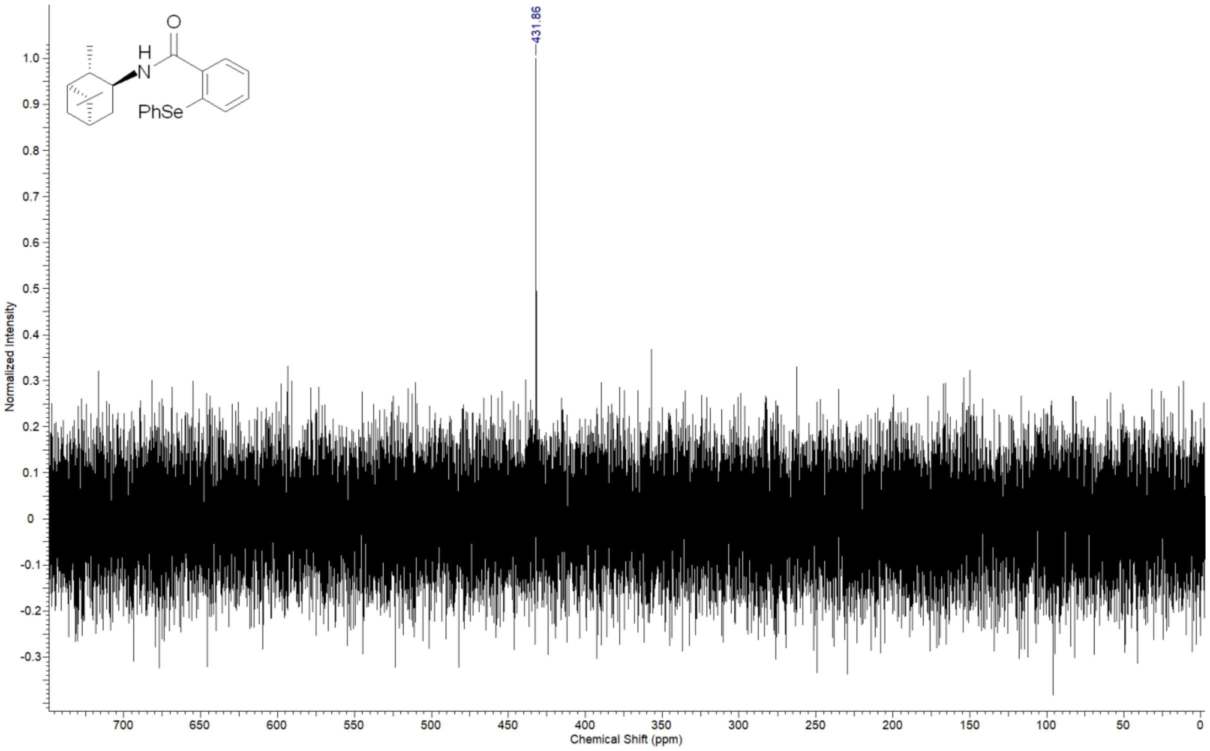

IV. References:

[1] Kumakura, F.; Mishra, B.; Priyadarsini, K.I.; Iwaoka, M. A Water-Soluble Cyclic Selenide with Enhanced Glutathione Peroxidase-Like Catalytic Activities. *Eur. J. Org. Chem*. **2010**, *3*, 440–444.

[2] Mosmann, T. Rapid colorimetric assay for cellular growth and survival: Application to proliferation and cytotoxicity assays. *J. Immunol. Methods*, **1983**, *65*, 55–63.
